# Evidence of an Antimicrobial Peptide Signature Encrypted in HECT E3 Ubiquitin Ligases

**DOI:** 10.3389/fimmu.2016.00664

**Published:** 2017-01-09

**Authors:** Ivan Lavander Candido-Ferreira, Thales Kronenberger, Raphael Santa Rosa Sayegh, Isabel de Fátima Correia Batista, Pedro Ismael da Silva Junior

**Affiliations:** ^1^Special Laboratory for Applied Toxinology (LETA), Center of Toxins, Immune-Response and Cell Signaling (CeTICS), Butantan Institute, São Paulo, São Paulo, Brazil; ^2^Biosciences Institute, University of São Paulo, São Paulo, São Paulo, Brazil; ^3^Department of Parasitology, Biomedical Sciences Institute, University of São Paulo, São Paulo, São Paulo, Brazil; ^4^Department of Biochemistry, Institute of Chemistry, University of São Paulo, São Paulo, São Paulo, Brazil; ^5^Laboratory of Biochemistry and Biophysics, Butantan Institute, São Paulo, São Paulo, Brazil

**Keywords:** HECT ligases, host defense, immune evolution, innate immunity, Nedd4, synergy, ubiquitination, ubiquitin–proteasome system

## Abstract

The ubiquitin-proteasome pathway (UPP) is a hallmark of the eukaryotic cell. In jawed vertebrates, it has been co-opted by the adaptive immune system, where proteasomal degradation produces endogenous peptides for major histocompatibility complex class I antigen presentation. However, proteolytic products are also necessary for the phylogenetically widespread innate immune system, as they often play a role as host defense peptides (HDPs), pivotal effectors against pathogens. Here, we report the identification of the arachnid HDP oligoventin, which shares homology to a core member of the UPP, E3 ubiquitin ligases. Oligoventin has broad antimicrobial activity and shows strong synergy with lysozymes. Using computational and phylogenetic approaches, we show high conservation of the oligoventin signature in HECT E3s. *In silico* simulation of HECT E3s self-proteolysis provides evidence that HDPs can be generated by fine-tuned 26S proteasomal degradation, and therefore are consistent with the hypothesis that oligoventin is a cryptic peptide released by the proteolytic processing of an Nedd4 E3 precursor protein. Finally, we compare the production of HDPs and endogenous antigens from orthologous HECT E3s by proteasomal degradation as a means of analyzing the UPP coupling to metazoan immunity. Our results highlight the functional plasticity of the UPP in innate and adaptive immune systems as a possibly recurrent mechanism to generate functionally diverse peptides.

## Introduction

The ubiquitin-proteasome pathway (UPP) is central to the eukaryotic cell, being involved virtually in every intracellular pathway, including protein posttranslational modifications, fine-tuned proteolysis, autophagy, cell cycle regulation, programed cell death, cell signaling, transcriptional regulation, gene expression, protein and mRNA turn over, cancer development, viral budding, and immune evasion by pathogens (1–10). Precise posttranslational modifications of proteins by ubiquitin or ubiquitin-like proteins involve the multistep, hierarchical transfer of ubiquitin to a substrate by ubiquitin-activating enzymes (E1), ubiquitin-conjugating enzymes (E2), and ubiquitin-protein ligases (E3) (1–10). Repeating this process generates polyubiquitin chains, which function as a signal for degradation via the 26S proteasome (1–10). In addition to the temporal, spatial, and context-specific regulation of the UPP, the large number of UPP targets is tightly regulated by the specificity-conferring components, E3s (3–7). As part of jawed vertebrates’ immune systems, E3s specify host defense signal transduction pathways, transcriptional regulation, and targeted proteolysis of cytosolic proteins for production of endogenous antigens, which are then presented to cytotoxic T cells mediated by major histocompatibility complex (MHC) class I receptors (1–10). Remarkably, bacteria have also evolved E3 ligases mimicking eukaryotic ones as an adaption to evade host defense (10–15).

In contrast to the adaptive immune system, which is restricted to jawed vertebrates and is based on humoral and cellular responses with specificity for antigens (1–10, 16), the phylogenetically widespread and more ancient innate defense relies on pattern recognition molecules conferring specificity against pathogens, complex signaling pathways leading to phagocytosis, encapsulation, and production of effector molecules with broad activity against pathogens (3, 4, 16–21). Recently, emerging roles in the innate immune system have been attributed to ubiquitin (22–29). Ubiquitin degradation produces host defense peptides (HDPs) (22–28), pivotal players in the innate defense against microbial pathogens (17–19). Additionally, more than 70 immunosuppressive peptides have been discovered originating from ubiquitin degradation *in vitro* (22, 30, 31). However, production of antimicrobial and immunomodulatory peptides is a complex, multilayered process (17–19, 22, 26, 28, 30, 31): it involves the canonical expression of transcriptionally regulated gene-encoded peptides (17–19) and fast production of cryptic peptides [that is, release of protein fragments with distinct properties from that of the original protein (19, 22, 26, 28)]. Therefore, the UPP orchestrates biologically active peptide production in both the innate and the adaptive metazoan immune systems by generating the recently discovered ubiquitin-encrypted HDPs (22–28), E3-mediated transcriptional regulation of host defense gene expression (3, 4, 6, 7, 10), and production of short fragments for MHC class I-mediated antigen presentation (3, 4, 8, 9, 16).

However, ubiquitin is highly constrained, with only three amino acids varying between yeast and human primary sequences (1–7, 22). Such constraint greatly reduces its potential to generate diverse HDPs. In contrast, E3s are larger, highly diversified, evolutionarily less conserved, and hold high affinity toward their molecular targets (1–7), which probably makes them a more enriched substrate than ubiquitin for encrypted HDPs. Surprisingly, little is known about the physiological roles that proteasomal degradation-derived fragments play, except for MHC class I antigens (3, 4, 8, 9, 16). Here, we hypothesize that similar to ubiquitin, E3 ligases harbor a defensive arsenal that can be released by fine-tuned proteolysis and thereby represent a novel scaffold for cryptic peptide discovery.

Among organisms that lack the adaptive immune system, arachnids’ innate defenses are remarkably enriched for HDPs (19–21). Identification of many classes of antimicrobial peptides that appeared early in evolution (17–21), such as glycine-rich peptides (19, 32, 33), tachyplesin-like HDPs (19, 34), defensins (19–21), lysozymes (20), and hemocyanin-derived antifungal peptides (19, 35) are consistent with their phylogenetic position at the base of extant arthropods’ phylogeny (19, 20, 34–36). Thus, investigating the arachnid innate immune peptidome is a promising approach to identify ancient host defense effectors, including cryptic HDPs.

Here, we describe the discovery and characterization of oligoventin, an arachnid HDP isolated from the eggs of the Brazilian armed spider *Phoneutria nigriventer* (Ctenidae, Araneomorphae). Based on bioinformatics analysis, we suggest that oligoventin is a cryptic peptide derived from the proteasomal degradation of E3s. Bayesian phylogenetic analysis indicates that oligoventin appeared early in evolution and its production is likely not restricted to arachnids. Moreover, oligoventin inhibits growth of yeast, Gram-positive and Gram-negative bacteria and also exhibits synergy with lysozymes against *Micrococcus luteus* A270, consistent with the proposed function as a host defense effector. Furthermore, investigating mouse and human immune epitopes derived from the proteasomal degradation of E3s uncovered eight sequences, which are indeed involved in MHC class I antigen presentation. Our results provide, to our knowledge, the first evidence that proteasomal-mediated protein degradation evolved independently to produce functional short-sized peptides in the adaptive immunity of jawed vertebrates and possibly in the innate defense of arachnids, thus highlighting a recurrent role of the UPP in the generation of functionally diverse peptides in metazoan immune systems.

## Materials and Methods

### Animals

Adult female *P. nigriventer* spiders laid eggs in captivity. Eggs were separated from silk and stored at −20°C for later use. These animals were collected under license Permanent Zoological Material no. 11024-3-IBAMA and Special Authorization for Access to Genetic Patrimony no. 001/2008.

### Microorganisms

Fungal and bacterial strains were obtained from various sources. *Escherichia coli* SBS363 and *M. luteus* A270 were from the Pasteur Institute (Paris); *Candida albicans* (MDM8) was from the Department of Microbiology from the University of São Paulo (Brazil); *E. coli* ATCC 25922, *Pseudomonas aeruginosa* ATCC 27853, *Serratia marcescens* ATCC4112, *Staphylococcus aureus* ATCC 29213, and *Staphylococcus epidermidis* ATCC 12228 were from the American Type Culture Collection (ATCC). The following human clinical yeast isolates, which can be agents of candidiasis, obtained from the Oswaldo Cruz Institute (Brazil) were also used: *Trichosporon* sp. IOC 4569, *Candida krusei* IOC 4559, *Candida glabrata* IOC 4565, *C. albicans* IOC 4558, *Candida parapsilosis* IOC 4564, *Candida tropicalis* IOC 4560, and *Candida guilliermondii* IOC 4557.

### Activity-Guided Isolation of Host Defense Effectors from *P. nigriventer* Eggs

**Figure 1:**
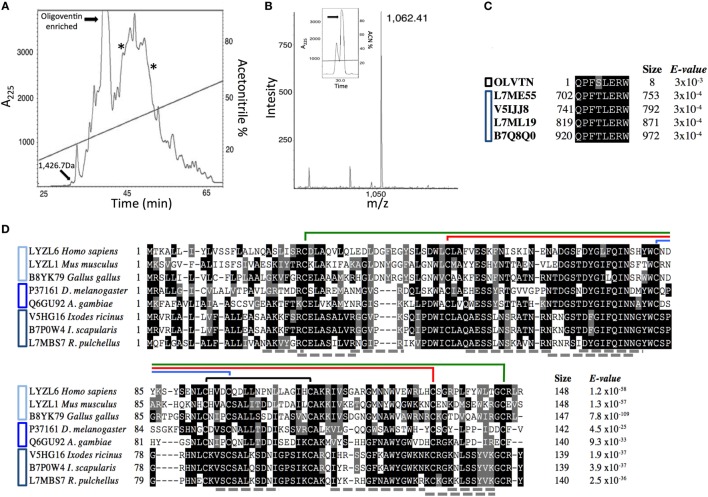
**Isolation of *Phoneutria nigriventer* host defense effectors**. **(A)** Semipreparative C_18_ reversed-phase high-performance liquid chromatography (RP-HPLC) of Sep-Pak C_18_ concentrated *P. nigriventer* non-infected eggs acidic extract. Highlighted fractions eluted between 20 and 55% acetonitrile. The fractions containing a peptide with 1.4 kDa and the oligoventin-enriched one were active against *Micrococcus luteus* A270. Fractions containing lysozymes are indicated with asterisks. These fractions were active against *Candida albicans* MDM8 and *M. luteus* A270, respectively. Absorbance was measured at 225 nm (*A*_225_). **(B)** MALDI spectra of native oligoventin. The inset shows the final purification of oligoventin. *m*/*z*, mass/charge ratio. **(C)** Multiple sequence alignment (MSA) showing evolutionary conservation of HECT domain C-terminal sequences from arachnids and oligoventin. Black and blue bars to the left of the alignment indicate oligoventin and Nedd4 proteins, respectively. **(D)** MSA showing evolutionary conservation of C-type lysozymes from vertebrates, insects, and arachnids. Shaded in black are highly conserved amino acid residues, whereas gray indicates moderately conserved residues. Connecting lines above sequences represent disulfide bridges. Structural coordinates were extracted from *Gallus gallus* C-type lysozyme (PDB entry 2LYZ). Cyan, blue, and navy blue bars to the left of the alignment indicate lysozymes from vertebrates, insects, and arachnids, respectively. Gray dotted lines represent tryptic peptides predicted by tandem MS.

Purification of antimicrobials was carried out following the strategies described elsewhere (37, 38). In brief, eggs were suspended in 20 mL of glacial acetic acid and homogenized. The insoluble material was removed by centrifugation at 16,000 *g* for 30 min. The supernatant was partially purified by applying it in two Sep-Pak C_18_ (Light tC_18_—Water Associates) cartridges connected in series equilibrated in 0.05% trifluoroacetic acid, and protein-concentrated fractions were eluted in three steps using 5, 40, and 80% of acetonitrile in acidified water. Only the protein-concentrated fraction eluted in 40% acetonitrile was directly used for HPLC purifications. Fractionation was carried out using a reversed-phase high-performance liquid chromatography (RP-HPLC) semipreparative C_18_ column (Jupiter, 10 × 250 mm), equilibrated in 2% acetonitrile, and 0.05% trifluoroacetic acid. Elution was successfully performed with a linear 2–60% gradient of solution B [0.10% (v/v) trifluoroacetic acid in acetonitrile] in acidified water {solution A [0.05% (v/v) trifluoroacetic acid in water]} run for 60 min at a flow rate of 1.5 mL/min. Effluent absorbance was monitored at 225 nm. Fractions with antimicrobial activity were further purified using an analytical Jupiter C_18_ column (250 mm × 4.6 mm) at a flow rate of 1.0 mL/min in 60 min with distinct gradients: from 33.5 to 43.5% of acetonitrile in acidified water for fraction containing the HDP of 1.4 kDa (Figure S1A in Supplementary Material), 22.5 to 32.5% for the oligoventin-enriched fraction (Figure 1B), 27.5 to 37.5% for the anti-*M. luteus* lysozyme (Figure S1C in Supplementary Material), and 34.5 to 44.5% for the anti-*C. albicans* lysozyme (Figure S1D in Supplementary Material). A symmetrical peak on the HPLC system, amino acid sequencing, and mass spectrometry analysis ascertained the purity of the peptide or protein. Fractions were lyophilized in a SpeedVac Concentrator.

### Growth Inhibition Assays

During the purification procedure, antimicrobial activities were detected or monitored by liquid growth inhibition assays as described in Ref. (38–40), using the Gram-negative bacteria *E. coli* SBS363 and Gram-positive bacteria *M. luteus* A270 that were cultured in poor broth nutrient medium (PB: 1.0 g peptone in 100 mL of water containing 86 mM NaCl at pH 7.4; 217 mOsM), and the yeast strain *C. albicans* MDM8, cultured in poor dextrose broth (1/2 PDB: 1.2 g potato dextrose in 100 mL of H_2_O at pH 5.0; 79 mOsM) used at half strength. Determination of antimicrobial activity was performed using fivefold micro titer broth dilution assay in 96-well sterile plates at a final volume of 100 mL. Mid-log phase culture was diluted to a final concentration of 1 × 10^5^ colony forming units/mL. Dried fractions were dissolved in 200 µL of ultrapure water and 20 µL applied in to each well and added to 80 µL of the bacterium/yeast dilution. A total of 100 µL of sterile water and PB or PDB were used as quality controls. Tetracycline and/or amphotericin B were also used as controls for growth inhibition. The microtiter plates were incubated for 18 h at 30°C; growth inhibition was determined by measuring absorbance at 595 nm.

Minimal inhibitory concentrations (MICs) were determined using the purified peptide against Gram-negative, Gram-positive, fungal, and yeast strains. MIC determination was performed using a fivefold microtiter broth dilution assay of stock solution, and serial dilution in 96-well sterile plates at a final volume of 100 µL where 20 µL of stock solution was applied in to each well at serial dilution twofold microtiter broth dilution and added to 80 µL of the bacterium/yeast dilution. MIC is defined as the minimal concentration of peptide that caused 100% growth inhibition (33–35, 38, 39). In an attempt to test how rich broth medium affects oligoventin activity, growth inhibition assays were carried out using RPMI-1640 (Sigma-Aldrich) with MOPS 0.165 mol/L (RPMI without bicarbonate 10.4 g/L; MOPS [3-(*n*-morpholino)propanesulfonic acid] 34.53 g/L) at pHs 7.0 and 5.0 against *C. albicans* MDM8.

Synergy was measured by checkerboard titration assays using a fivefold microtiter broth dilution assay of stock solution and serial dilution in 96-well sterile plates at a final volume of 100 µL. Oligoventin was diluted along the rows of a microtiter tray and the avian lysozyme was diluted along the columns. A total of 80 µL of *M. luteus* was added to each well, diluted to a final concentration of 1 × 10^5^ colony forming units/mL. The fractional inhibitory concentration (FIC) was determined after 18 h of incubation of the plates at 30°C. Synergy was defined as an FIC index of 0.5 or less as it represents at least fourfold decrease in the MIC of each compound (41), calculated according to the following formula: FIC index = [A]/MIC_A_ + [B]/MIC_B_, where [A] was the concentration of drug A in a well that represented the lowest inhibitory concentration in its row, MIC_A_ was the MIC of drug A alone, [B] was the concentration of drug B in a well that represented the lowest inhibitory concentration in its row, and MIC_B_ was the MIC of drug B alone. Growth inhibition was determined by measuring absorbance at 595 nm. Bioassays were done in triplicate.

### Oligoventin Toxicity to Erythrocytes

The hemolytic activity of oligoventin was tested in duplicate using human erythrocytes. A 2.5% (v/v) suspension of erythrocytes washed in PBS was incubated with oligoventin ranging from 0.4 to 188.8 µM in a 96-well plate for 3 h with intermittent shaking. The absorbance in the supernatant was measured at 415 nm. Hemolysis caused by PBS and 1% (v/v) Triton X-100 were used as 0 and 100% controls, respectively.

### Molecular Mass Characterization and Sequence Determination

The fractions enriched for peptides were spotted (0.5 µL) onto the sample slide, dried on the bench, and crystallized with 0.5 µL of matrix solution [5 mg/mL (w/v) CHCA (α-cyano-4-hydroxycinnamic acid), in 50% acetonitrile and 0.1% TFA] (Sigma). The samples were analyzed on an Ettan MALDI-ToF/Pro spectrometer (Amersham Biosciences) operating in reflectron and positive ion mode. To determine the amino acid sequence of peptides, Edman degradation was performed in a PPSq 21 Automated Protein Sequencer (Shimadzu Co., Japan). Lysozymes were analyzed by SDS polyacrylamide gel electrophoresis (12.5% SDS-PAGE). In-gel lysozymes were destained, dehydrated in 100% acetronitrile for 10 min, and lyophilized. Freeze-dried purified protein was dissolved (1 mg/mL) in denaturant buffer [6 M GdmCl (guanidinium chloride), 0.25 M Tris/HCl, and 1 mM EDTA, pH 8.5]. A total of 20 µL of 2-mercaptoethanol (Sigma) was added to the mixture, followed by vortex-mixing and incubating at 37°C for 2 h. After incubation, 100 µL of 4-vinylpyridine was added to the solution, followed by incubation at room temperature (26°C) for 2 h. The reduction and alkylation of the protein were confirmed by mass spectrometry. Reduced and alkylated proteins were digested with trypsin (Boehringer Mannheim) and tryptic peptides were analyzed by tandem mass spectrometry (MS/MS) in a Q-TOF Ultima API (Micromass) spectrometer operating in positive ion mode. In the mass spectrometer, doubly charged ions of sufficient abundance were selected for MS/MS fragmentation. MS/MS peak list files were submitted to an in-house version of MASCOT server (Matrix Science, USA) and screened against the Uniprot database. Representative resulting spectra and corresponding tables are provided in Table S1 in Supplementary Material.

### Arachnid Genome Screening

BLAST searches were done against the *Ixodes scapularis* IscaW1 genome (42), a well-annotated arachnid genome resource, using oligoventin sequence as the query. Peptide–protein matching was adjusted with the following stringent settings: word size: 2; filter off; *e*-value 20,000; composition-based statistic off; PAM30 scoring matrix.

### HECT Domain Homology Modeling and Surface Mapping of Conserved Residues

The 3D model of the HECT domain was generated using the online server HHPred (43) for template identification and Modeller 9v15 (44) for the model construction. PDB entry 2ONI was used as template (71% similarity). The quality of the final structure was accessed using MolProbity (45) showing just one residue out of the Ramachandran allowed region (Ala 61) and 99.02% of the residues placed on the favorable region. Rate of amino acid evolution among Nedd4 HECT E3s was calculated from 50 homologs using maximum-likelihood phylogenetic analysis and mapped onto the protein structure using default parameters with Consurf (46).

### Computational Simulations of Proteasomal Degradation

Human proteasome cleavage predictions were simulated with a stringent 0.7 threshold for four sequences (Table S3 in Supplementary Material) using the neural network algorithm Netchop3.1 online server “http://www.cbs.dtu.dk/services/NetChop/.” We used the C-term 3.0 network, which is trained on 1,260 naturally occurring MHC class I ligands (8, 47).

### Phylogenetic Reconstruction of Eukaryotic Nedd4 Diversification

Bayesian phylogenetic inference was carried out with modifications from Ref. (48). Orthologous sequences containing the HECT domain (PF00632) were collected from a large number of eukaryotes. A complete list of Uniprot identifiers was used for the acquisition of 4,392 HECT-containing protein sequences. Redundancy in our dataset was minimized by employing a clustering methodology using the CD-HIT software (49) with a threshold of >90%. One representative protein from each cluster was retrieved for further analysis. This yielded 318 protein sequences relative to 370 eukaryotic species (Table S2 in Supplementary Material), which were aligned with T-coffee (50). The final alignment was manually edited in GeneDoc (51) resulting in 2,470 sites. The choice of the best-fit model of evolution was performed with ProtTest3 (52) using the Akaike Information Criterion, which led us to choose the WAG model. The phylogenetic reconstruction was inferred by the Bayesian inference method implemented in the Beast v1.7.0 software (53, 54). The starting tree was randomly generated, and the proportion of invariable sites and g-distributed rate variation across sites were estimated. The substitution rate categories were set in four categories, and we modeled the molecular clock accordingly to relaxed clock model available (55). The clades were supported by posterior probabilities obtained by Bayesian analysis. For Bayesian method generations, the burn-in was determined in Tracer (54) through log-likelihood scores, and data were summarized in TreeAnnotator (54) after trees that were out of the convergence area had been discarded. A total of 10,000,000 trees were generated, from which 25,000 were burned out of the final tree. The visualization and the final tree edition were performed using FigTree v1.3.1 “http://tree.bio.ed.ac.uk/software/figtree/.” Finally, proteins belonging to each phylogenetic cluster were dissected for revealing the oligoventin-orthologous sequence, from which sequence logos belonging to each clade were generated in Weblogo (56).

### Epitope Comparison

The Immune Epitope Database (57) was screened using Nedd4 E3 HECT proteins as queries and yielded nine MHC ligands.

## Results

### Identification of Host Defense Effectors

We combined solid-phase purification with assay-guided RP-HPLC runs to isolate host defense effectors from an acidic extract from *P. nigriventer* non-infected eggs. Four fractions with antimicrobial activity were found (Figure 1A). Mass analysis indicates that they were enriched for innate immune defense effectors, namely, HDPs and lysozymes (Figure S1 in Supplementary Material). To isolate these host defense effectors, analytical RP-HPLC runs coupled with bioassays yielded two fractions with molecules ranging in size from 0.8 to 1.7 kDa (Figures S1A–C in Supplementary Material), which were active against the Gram-positive bacteria *M. luteus* A270, and two lysozyme-like molecules ranging in size from 14 to 16 kDa (Figures S1D–F in Supplementary Material). A peptide with a mass of 1.4 kDa (Figure S1A in Supplementary Material) was purified to homogeneity, and Edman degradation showed that this peptide was N-terminally blocked. Further investigation by tandem mass spectrometry is needed to sequence this putative HDP. The fraction enriched for oligoventin (Figure 1A) was further purified. N-terminal sequencing by Edman degradation revealed an oligopeptide with eight residues: QPFSLERW, which we named oligoventin. Matrix-assisted laser desorption/ionization—time of flight mass spectrometry (MALDI-ToF-MS) analysis of the resulting fraction shows that the oligoventin molecular weight (MW) observed 1,061.4 Da (M + H^+^ = 1,062.4, Figure 1B) corresponds to the MW calculated (1,061.5 Da).

Fractions enriched for lysozymes (Figures S1D–F in Supplementary Material) were active against the Gram-positive bacteria *M. luteus* A270 and the yeast *C. albicans* MDM8, respectively, and were further fractionated by C_18_ RP-HPLC. Reduction, alkylation, and trypsinization of these fractions followed by comparison of the resulting tryptic peptides by LC-ESI-MS/MS suggest that both of these antimicrobial factors are C-type lysozymes (Table S1 in Supplementary Material). Oligoventin and the putative HDP with 1.4 kDa show similar relative abundances. Altogether, these four antimicrobials represent less than 4% of the total protein content (Figure S2 in Supplementary Material).

### Oligoventin Antimicrobial Activity and Synergy with Lysozymes

Oligoventin presents antimicrobial activity and MICs in the micromolar range (Table 1), markedly against Gram-positive bacteria: *M. luteus* A270 and the multi-resistant *S. aureus* ATCC 29213 and *S. epidermidis* ATCC 12228 strains. It is also active against the yeast *C. albicans* MDM8 and the Gram-negative bacteria *S. marcescens* ATCC 4112. However, it has antimicrobial activity in concentrations higher than those of rondonin (35), a cryptic peptide derived from the oxygen-carrier protein hemocyanin (19, 35). Oligoventin is active at concentrations ranging from 47 to 188.9 µM, while rondonin is active from 16.5 to 33.5 µM, except for the *Tricosporon* sp. IOC 4569 fungi strain, which is 2.1 µM (35). In contrast, oligoventin inhibits growth of Gram-positive, Gram-negative, and yeast strains, thus having a broader spectrum of antimicrobial activity compared to that of rondonin.

**Table 1:** **Minimal inhibitory concentrations (MICs) of HDPs from arachnids**.

Microorganisms	MIC (μM)	
	Rondonin (35)	Oligoventin
**Gram-positive bacteria**		
*Micrococcus luteus* A270	ND	47.2–94.5
*Staphylococcus aureus* ATCC 29213	ND	94.5–188.9
*Staphylococcus epidermidis* ATCC 12228	ND	94.5–188.9
**Gram-negative bacteria**		
*Escherichia coli* SBS363	NT	ND
*Escherichia coli* ATCC 25922	NT	ND
*Pseudomonas aeruginosa* ATCC 27853	NT	ND
*Serratia marcescens* ATCC 4112	NT	23.53–47.2
**Fungi**		
*Trichosporon* sp. IOC 4569	1.1–2.1	ND
**Yeasts**		
*Candida albicans* MDM8	16.75–33.5	94.5–188.9
*Candida krusei* IOC 4559	16.75–33.5	ND
*Candida glabrata* IOC 4565	8.37–16.5	ND
*Candida albicans* IOC 4558	8.37–16.5	ND
*Candida parapsilosis* IOC 4564	16.75–33.5	ND
*Candida tropicalis* IOC 4560	8.37–16.5	ND
*Candida guilliermondii* IOC 4557	16.75–33.5	ND

*The highest concentration tested was 189 µM. ND, activity was not detected in the range of concentrations tested. NT, antimicrobial activity was not tested or previously not reported (35). Rondonin is an hemocyanin-encrypted antifungal HDP from the tarantula *Acanthoscurria rondoniae* (19, 35)*.

*MICs are expressed as the interval of two concentrations, where the first is the highest concentration tested at which microorganisms from each strain grew and the second is the lowest concentration tested that caused 100% growth inhibition (33–35, 38, 39)*.

C-type lysozymes, classical players of innate immunity, are ubiquitous and constitutively expressed in leukocytes, providing an immediate defensive barrier to invading pathogens (20, 41, 58). Because context-specific co-expression of lysozymes with other host defense elements such as HDPs suggests synergy between these factors (41, 59), we investigated whether oligoventin has a mutually potentiating effect on the *G. gallus* C-lysozyme, as C-type lysozymes from arachnids are highly similar to avian lysozymes (Figure 1D). We determined the MIC of avian C-lysozyme as 0.01–0.02 µM against *M. luteus* A270. When both factors were tested together against this strain, we found a 0.37 FIC index, indicating strong synergy (41) between these molecules (Table S2 in Supplementary Material). MIC of oligoventin is potentiated 8-fold by lysozymes, exerting its effect at a concentration as low as 11.81 µM, whereas oligoventin potentiates lysozymes by decreasing its MIC 3.5-fold, causing growth inhibition at 0.006 µM. Moreover, oligoventin exhibits 4.3% hemolytic activity against human erythrocytes at 188.9 µM and 2% at 94.5 µM (Figure S3 in Supplementary Material). However, both oligoventin and lysozymes did not display antimicrobial activity against *C. albicans* MDM8 when cultured in RPMI-1640 (Sigma-Aldrich) at pH 5.0 or 7.0, even when oligoventin concentration was increased twofold relative to its MIC (376 µM). Similarly, although we determined lysozymes as having a MIC of 1.3–2.6 µM against *C. albicans* MDM8 in poor dextrose broth medium, this classical HDP also lacked antimicrobial activity in RPMI-1640, even when lysozyme concentration was increased more than 50-fold relative to its MIC (data not shown).

### E3 Ubiquitin Ligases As Oligoventin Precursor Proteins

To determine the oligoventin precursor protein, we BLAST screened the blacklegged tick *I. scapularis* genome IscaW1.4 from Vectorbase (42), a high-quality genomic resource for arachnids. Results showed high homology (88%) between oligoventin and the C-terminal HECT (Homologous to the E6-AP Carboxyl Terminus) domain sequence between residues 920–927 of Nedd4 (neural precursor cell expressed, developmentally downregulated 4-like) E3 ubiquitin ligases (Figure 1C; Figures S4 and S5 in Supplementary Material). Consistently, BLAST screening the Uniprot database with adjustments for peptide–protein matching revealed similar results for orthologous Nedd4 from two arachnids (*Ixodes ricinus*, Uniprot ID: V5IJJ8, and *Rhipicephalus pulchellus*, Uniprot IDs: L7ML19, L7ME55) and one crustacean (*Daphnia pulex*, Uniprot ID: E9GKW9). These results suggest oligoventin is a cryptic peptide released by the proteolysis of a *P. nigriventer* Nedd4 protein ortholog. Alternative hypotheses of oligoventin precursor proteins are indicated in Table S3 in Supplementary Material.

**Figure 2:**
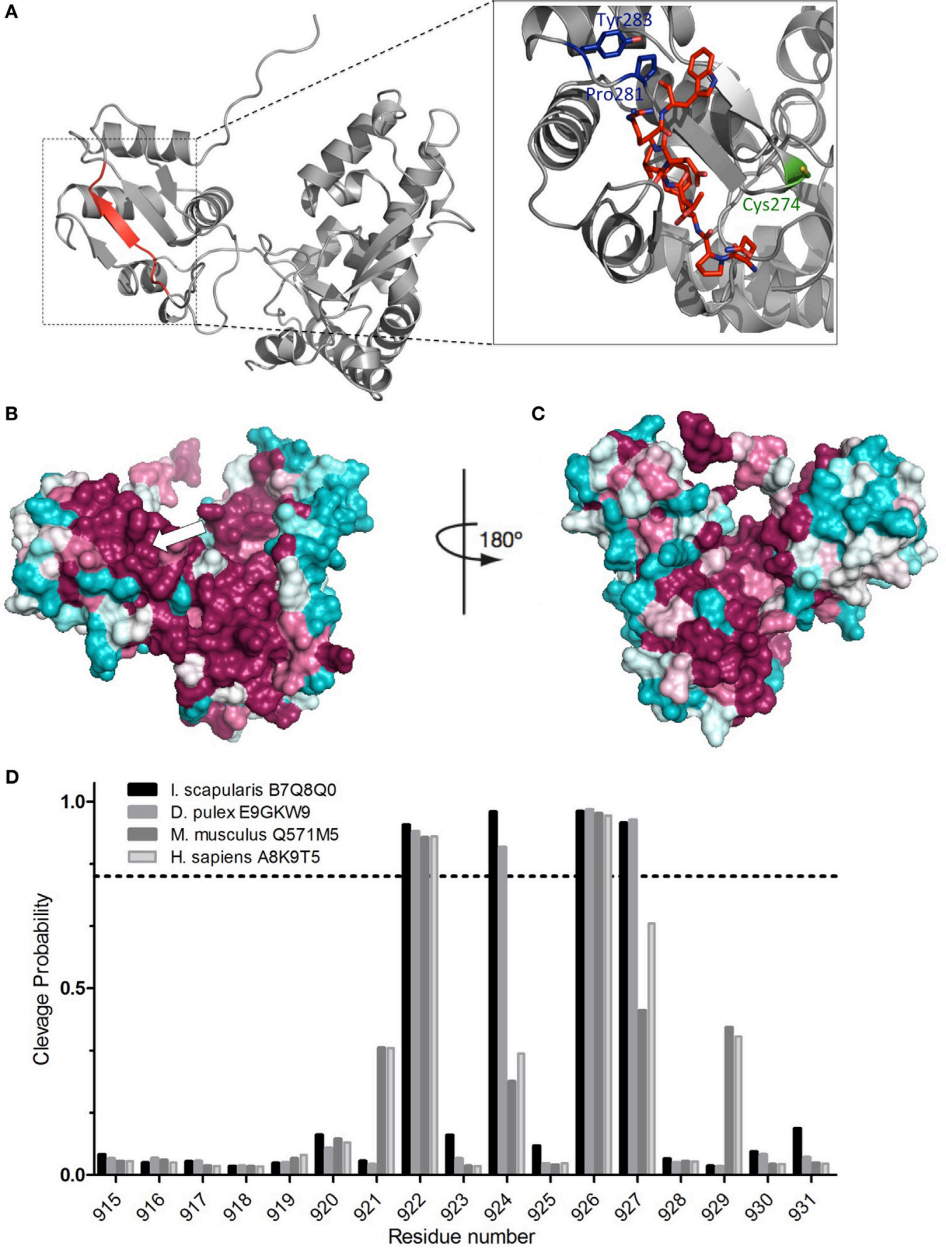
**Conservation of the oligoventin-encrypted site and *in silico* proteasomal degradation of E3s**. **(A)** Homology modeling of the HECT domain from the arachnid B7Q8Q0 Nedd4 E3 ubiquitin ligase. Oligoventin is encrypted in a site between residues 920 and 927 (colored in red), which folds as a β-sheet. The catalytic site (Cys 274) and the PY motif (Pro281, Tyr 285) are colored in green and blue, respectively. **(B,C)** Surface mapping of conserved sites onto the arachnid Nedd4 HECT domain three-dimensional model. The arrow indicates the oligoventin-encrypted site. Teal represents the most variable residues and burgundy the most highly conserved. Model figures were generated using Pymol. **(D)** Proteasomal cleavage sites predicted for the HECT domain of four E3 sequences from human, mouse, the blacklegged tick *Ixodes scapularis*, and the crustacean *Daphnia pulex*. The dashed line shows the 0.7 threshold.

3D molecular modeling of the blacklegged tick Nedd4 E3 (Uniprot ID: B7Q5Q0) HECT domain (Figure 2A) shows the oligoventin-homologous epitope folding as a β-sheet and its close position to the catalytic cysteine site and the PY motif. PY motifs are internal regulatory motifs that are recognized by WW domains. Notice that the internal PY motif from HECT domains is different from the PPxY motifs found in substrates targeted also by the WW domains of catalytic HECT E3 ligases [reviewed in Ref. (60)] and herein we refer only to the regulatory PY motif from HECT domains.

Surface mapping projection of orthologous sequences onto the 3D model shows high-sequence conservation in the oligoventin-homologous, the catalytic site, and the regulatory PY motif C-terminal sequences (Figures 2B,C). Such high-sequence conservation suggests that the oligoventin-encrypted site is functionally important across different taxa.

### *In Silico* Proteolytic Processing of E3 Ubiquitin Ligases by the 26S Proteasome

We used bioinformatics approaches to verify if oligoventin generation could occur by HECT E3s proteasome-mediated proteolysis. A small dataset (Figure S5 in Supplementary Material) consisting of only the C-terminal Nedd4 HECT domain of ubiquitin ligase sequences for two invertebrates (the arachnid *I. scapularis* and the crustacean *D. pulex*) and two vertebrates (mouse and human) were used as input in NetChop3.1, a neural network algorithm trained to predict 26S proteasomal cleavage sites both for constitutive and immunoproteasomes (8, 47). This approach is appropriate because tissue-specific proteasomes, namely, constitutive, immune-, or thymus-specific proteasomes, are structurally rearranged in ways that combine different regulatory and catalytic domains, thus yielding different products (3–6, 8, 9, 47, 61). Therefore, simulating several proteasomes in a single prediction method magnifies the possibility of accurately mapping multiple cleavage sites onto a template protein (8, 47). Figure 2D shows the site positioned within the amino acid residues 920–927 in which oligoventin shows marked homology (Figure 1C) is enriched for cleavage sites in all sequences evaluated. In contrast, the flanking residues lack cleavage sites (Figure S6 and Table S4 in Supplementary Material). These results suggest high conservation of cleavage sites within metazoan E3s. Therefore, our approach indicates that Nedd4 26S proteasome-mediated proteolysis might explain oligoventin production from an E3 ubiquitin ligase precursor protein.

### Phylogenetic Analysis Reveals an Ancient HDP Signature in Metazoan Nedd4s

**Figure 3:**
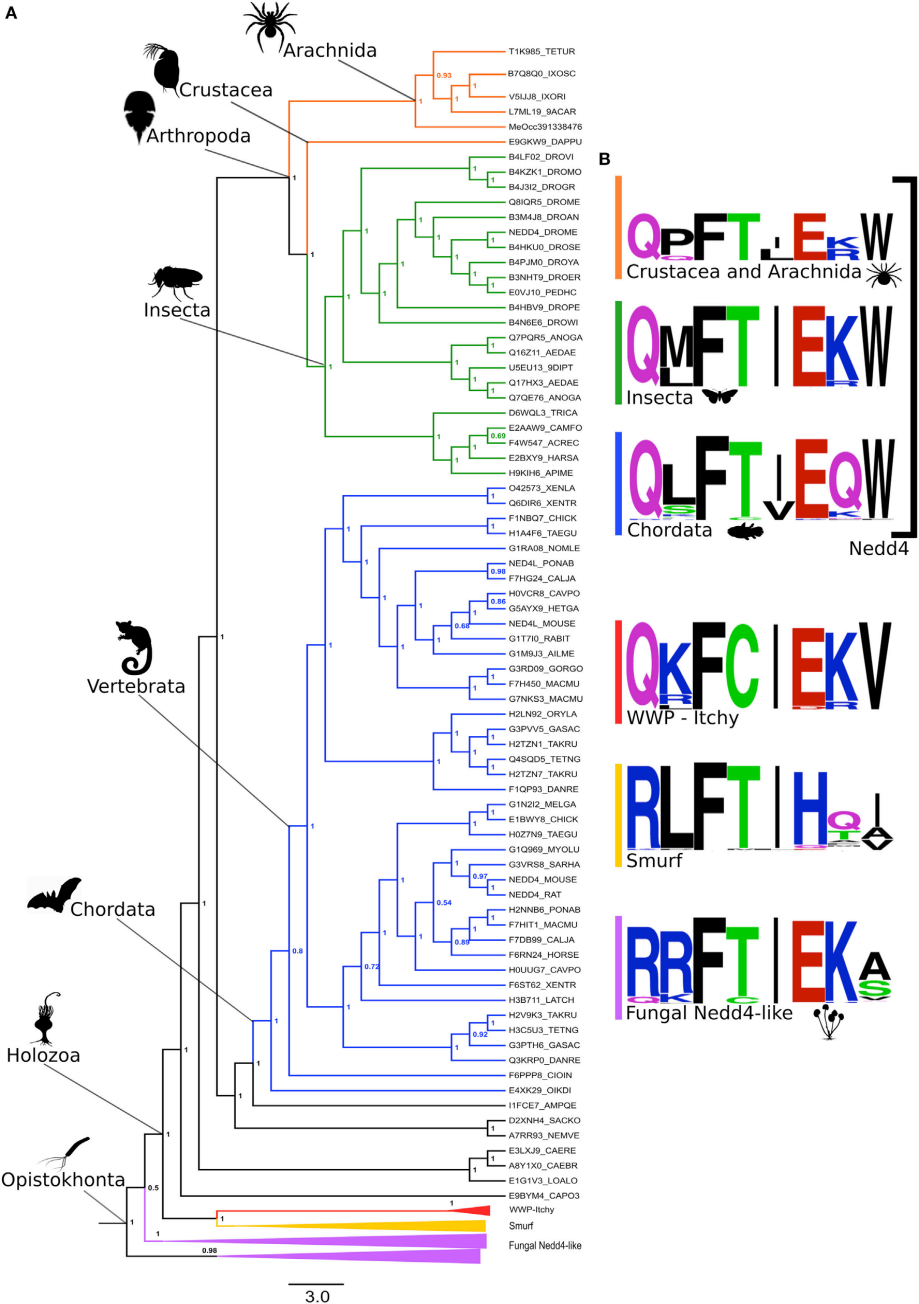
**Oligoventin signature conserved within metazoans suggests Nedd4 E3 ubiquitin ligases as the precursor protein candidate of oligoventin**. **(A)** Bayesian phylogenetic inference of 318 HECT-containing E3s. **(B)** Oligoventin-orthologous sequence signatures for major protein subfamilies comprised of Nedd4, WWP-Itchy, Smurf, and fungal Nedd4 HECT E3 ligases groups. Silhouettes from organisms are from Phylopic (http://phylopic.org/).

In spite of the HECT domain deep conservation (Figures 2B,C), HECT E3s are pervasive within eukaryotic genomes, comprising more than 33 protein families highly diversified in animals, which have undergone wide architectural rearrangement (1–7). Therefore, our structural conservation data do not inform to what extent the different HECT-containing E3 families may contribute to oligoventin production. To address this issue, we conducted a comprehensive Bayesian phylogenetic analysis on 318 orthologous sequences recovered from all major eukaryotic clades (Table S5 in Supplementary Material), which yielded a tree topology (Figure 3A) consistent with previous findings (2). Our results indicate that Nedd4 proteins are enriched for the oligoventin motif, but not WWP, Itchy, Smurf, and fungal Nedd4 HECT ligases. Figure 3B illustrates sequence logos for each corresponding encrypted site within sampled orthologous sequences. We found that Nedd4s are enriched for a motif composed of Q(P/M/L)F(S/T)(L/I)E(R/K/Q)W. In arthropods positioned near the base of extant ecdysozoans, namely, crustaceans and arachnids, the motif is more pronounced, with two conservative amino acid substitutions, one at the fourth position (S/T) and another at the seventh (K/R). The oligoventin sequence signature is also encrypted to some extent in Nedd4 from insects and vertebrates, with a single non-conservative change at the seventh position in chordates.

### Possible Convergent Evolution of the UPP in Metazoan Immune Systems

**Figure 4:**
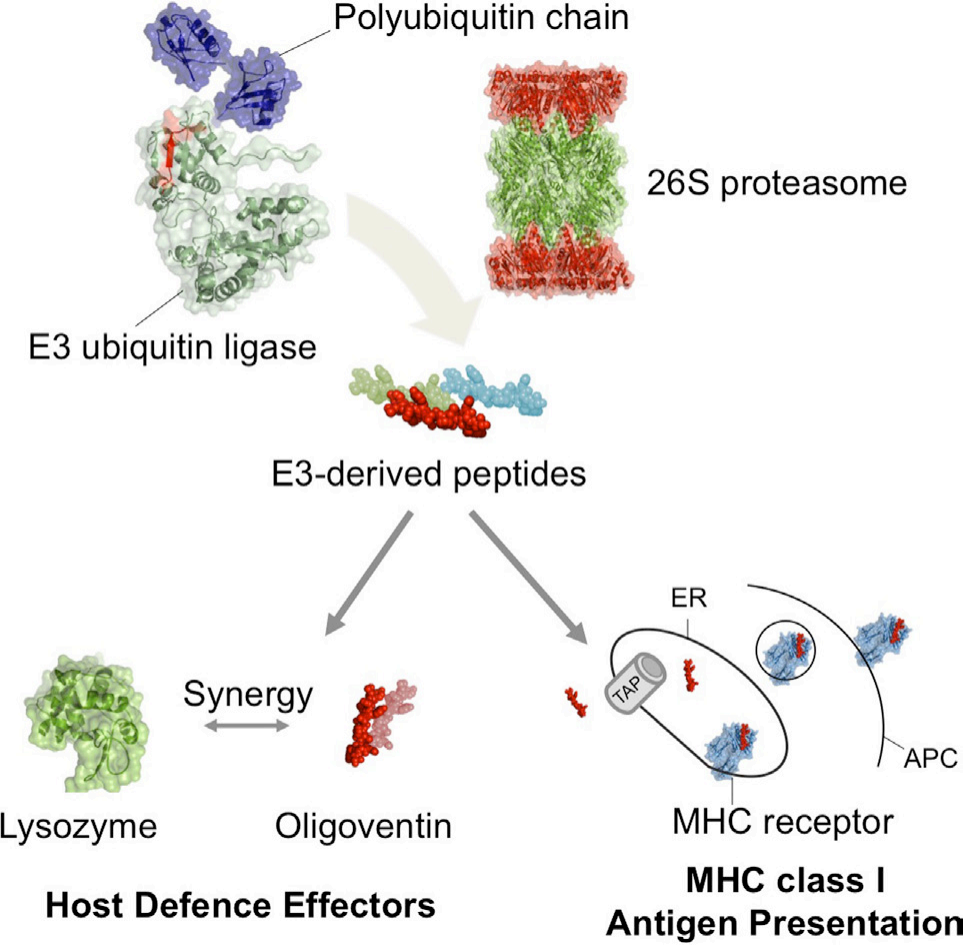
**Self-proteolysis of ubiquitin E3 ligases as a mechanism mediating production of functionally diverse peptides in metazoan host defense**. Our results suggest that putative ubiquitinated E3s undergo proteasome-mediated proteolysis, yielding E3-derived HDPs. In arachnids, the HDP oligoventin is predicted to be produced from the degradation of an Nedd4 E3 and may act as a host defense effector in combination with lysozymes. In jawed vertebrates, the proteasomal degradation of E3s produces antigens, which are then transported by ATP-binding cassette proteins (TAPs) and assembled in the endoplasmic reticulum (ER) with MHC class I receptors for presentation in antigen-presenting cells (APCs). Therefore, E3s degradation mediated by the ubiquitin–proteasome system might have been independently repurposed multiple times during metazoan evolution to play roles in the immune system as functionally diverse as endogenous antigens and host defense effectors.

Because all nucleated cells from jawed vertebrates present their own antigens derived from cytosolic proteins to cytotoxic T cells through MHC class I (3, 4, 8, 9, 16, 47, 57), we investigated if human and mouse Nedd4 proteins (7, 60) were involved in antigen presentation as a means of comparing the contribution of HECT E3s to the adaptive and innate immune systems. Screening the Immune Epitope Database (57) for Nedd4-derived antigens yielded nine peptides, ranging in size from 8 to 16 residues (MWs between 0.9 and 1.2 kDa), indeed involved in antigen presentation as revealed by MHC ligand assays (Table 2). These results strongly support the hypothesis of convergent evolution of the UPP function in immunity. Therefore, it seems that the UPP was co-opted multiple times in immune systems. Whereas in mammals, Nedd4 E3s play a role in the adaptive immune system as a core member of the UPP (2, 7, 9, 16, 47, 57, 60, 62) and as precursors for MHC class I antigens in mammals (e.g., Table 2), it is possible that a function for E3s has also evolved in the innate defense as precursors of HDPs in a pathway likely dependent on the ubiquitin–proteasome system, at least in arachnids (Figure 4).

**Table 2:** **Endogenous antigens derived from E3 ligases from the Nedd4 family involved in MHC-mediated antigens presentation**.

Epitope ID	Peptide	MW	Antigen name	Species	Method	MHC allele name	MHC Class
191812	SGLCNEDHL	986.4	E3 ubiquitin-protein ligase 4-like isoform 1	*Mus musculus*	Cellular MHC/mass spectrometry	H2-Db	I
214505	LPFEKSQL	960.5	NEDD4-like E3 ubiquitin-protein ligase WWP2 isoform 3	*Homo sapiens*	Cellular MHC/mass spectrometry	HLA-B*08:01	I
225136	YFDEKELEL	1,184.5	NEDD4-like E3 ubiquitin-protein ligase WWP2	*H. sapiens*	Cellular MHC/mass spectrometry	HLA-C*04:01	I
241145	ARAPAPYKR	1,184.5	E3 ubiquitin-protein ligase HECW2	*H. sapiens*	Mass spectrometry	HLA-B*27:05	I
241758	GGSARTATAASEQSPG	1,028.5	NEDD4-like E3 ubiquitin-protein ligase WWP2	*M. musculus*	Mass spectrometry	HLA-DQ8	II
422506	KSRPIIKRF	1,446.6	E3 ubiquitin-protein ligase HECW1	*H. sapiens*	Cellular MHC/mass spectrometry	HLA-B*57:01	I
422718	SGLCNEDHL + OX(C_4_)	1,143.7	E3 ubiquitin-protein ligase NEDD4-like isoform 1	*M. musculus*	Cellular MHC/mass spectrometry	H2-class I	I
424855	GFLRLKMAY	986.4	E3 ubiquitin-protein ligase NEDD4-like isoform 6	*H. sapiens*	Cellular MHC/mass spectrometry	HLA-A*29:02	I
429266	ASDPYVKLSLY	1,097.5	E3 ubiquitin-protein ligase NEDD4-like	*H. sapiens*	Cellular MHC/mass spectrometry	HLA-A*01:01	I

*MW, molecular weight*.

*Antigens were retrieved from the Immune Epitope Database (57)*.

## Discussion

Our analysis suggests that a novel player in the ancient yet diverse innate immune system from arachnids (17, 19–21, 34, 35), oligoventin, shares homology to the catalytic HECT domain of metazoan Nedd4 E3 ubiquitin ligases. Computational dissection of hundreds of HECT E3 ubiquitin ligases indicates that production of oligoventin-like HDPs might be limited to Nedd4 orthologs, as the oligoventin signature is encrypted in metazoan Nedd4s, but not in Nedd4s from fungi nor in the closely related metazoan WWP/Itch, Smurf and HECW HECT-containing E3s, which might represent paralogs of this family of ligases. However, it is also possible that other classes of E3s can be involved in releasing additional HDPs. In fact, the results summarized in Table 2 show that other HECT-containing E3s also produce functional peptides, such as HECW1 and HECW2; therefore, it is likely that other E3s can mediate the production of functional peptides, consistent with the low numbers of HECT-containing ligases in arachnid genomes (63). Indeed, while Nedd4 or Nedd4-2-deficient mice show a variety of phenotypes including embryonic and neonatal lethality (60), recent studies provided evidence that other classes of E3s (RING-containing) play a role in arachnid host defense, at least against the bacterial pathogen *Anaplasma phagocytophilum* (64–66), as suggested by silencing the E3 ligase XIAP in *I. scapularis* ticks (64). However, the underlying mechanism of the role of E3 in tick host defense remains elusive (64–66), and it would be interesting to test whether XIAPs are precursors of HDPs or are involved in the regulation of innate immune pathways.

The proposed homology between an antimicrobial peptide and the catalytic HECT domain of an E3 ubiquitin ligase immediately suggests a mechanism of oligoventin production by E3s self-proteolysis. E3 ubiquitin ligases are involved in the last step of ubiquitination, flagging substrates with ubiquitin for proteasomal degradation (1–8). Thus, E3s are the components that confer specificity to the UPP (3–6). E3s containing the HECT domain first form an intermediate thioester bond between the catalytic cysteine and ubiquitin before transferring this moiety to a lysine residue in the target substrate (4–6). In fact, the intermediate thioesther formation with ubiquitin is critical to HECT E3s in *cis* self-ubiquitination activity (5, 6). Hence, a parsimonious mechanism of generating the HDP oligoventin would be HECT E3s self-ubiquitination coupled with proteasomal degradation. Indeed, our *in silico* simulation of the proteolysis of E3 ligases is consistent with the idea that oligoventin is generated as a product of the proteasomal degradation of E3s. It might be, therefore, straightforward to recruit the UPP to generate diverse HDPs from targeted cytosolic proteins, as it is for production of MHC class I antigens (3, 4, 8, 9, 16, 62).

Nevertheless, we cannot rule out the possibility that oligoventin can be generated by non-self-ubiquitination of oligoventin precursors coupled with proteasomal degradation [that is, E3s in *trans* ubiquitination (5, 6)], or ubiquitin- or proteasomal-independent proteolysis [e.g., by selective macroautophagy (26–29)]. In fact, Nedd4 proteins preferentially conjugate the K63 linkage ubiquitin chain on substrate proteins, which alters the signaling properties or trafficking pattern of these modified proteins, instead of the K48 linkage ubiquitin chain that usually directs ubiquitinated proteins to proteasomal degradation (64, 67). Therefore, it is more likely that oligoventin precursors are ubiquitinated by other E3s conjugating K48 linkages. Furthermore, it can be argued that alternative mechanisms can explain production of short HDPs such as oligoventin. For example, long non-coding RNAs that produce short-sized biologically active peptides (68, 69) or proteasomal-independent production of encrypted HDPs (26–28) might underlie the generation of oligoventin. However, the biochemical, phylogenetic, and computational evidence presented here supports a model in which E3s release oligoventin by proteasomal proteolysis.

Oligoventin production by proteasomal degradation suggests that this peptide can be stored in intracellular compartments, similar to MHC class I antigens (8, 9, 47, 70) and then be directed to the extracellular space where it might play its functional role. Indeed, previous studies suggest that spider hemocytes (immune cells similar to mammalian macrophages) preferentially export antimicrobial effectors, such as acanthoscurrins and gomesins (33, 34), through exocytosis (71), contrasting with vertebrate macrophages, which preferentially display phagocytic activity against pathogens (71, 72). Therefore, the model in which oligoventin is derived from E3s proteasomal-mediated proteolysis could provide an extraordinary example of convergent evolution in which highly conserved orthologous pathways (e.g., TAP-dependent) direct proteasomal products to their extracellular site, despite functional divergence of those products. However, as some ubiquitin-encrypted HDPs are produced in autophagosomes (26–28), it is also possible that oligoventin can be produced by proteasomal-independent selective degradation of cytosolic Nedd4 proteins during macroautophagy of invading pathogens.

Together with the discovery of oligoventin, the presence of two lysozymes and a putative HDP of 1.4 kDa reveals hallmarks of an ancient immune defense system (17–21). Although oligoventin alone shows a relatively weak antimicrobial activity (Table 1), it has potent synergy with lysozymes, thus providing evidence of putative E3-derived peptides playing an important role in modulating the innate defenses of *P. nigriventer*. Moreover, because some arachnids lack inducible production of HDPs (17, 19–21, 34, 71), it is possible that oligoventin is constitutively expressed during early development, as it was discovered in non-infected eggs. Alternatively, antimicrobial factors such as HDPs, lysozymes, and antibodies are either maternally deposited or upregulated by parental imprinting within several taxa, including, but not limited to, cnidarians (73), insects (74, 75), amphibians (76), and amniotes (77), such as birds, rats, and humans, then it is possible that oligoventin has a maternal origin and could play a role in regulating early microbial colonization during development.

Future research can benefit from MALDI imaging mass spectrometry (78, 79) to investigate E3-mediated production of HDPs *in vivo*. Combining MALDI imaging with the recently developed ubiquitin variants (80), which systematically modulate HECT E3 ligase activity, as well as proteasome inhibitors (81), will be useful to probe the mechanism of HDP production proposed herein. Furthermore, the i5k initiative (82) aims to sequence 63 arachnid genomes, including *P. nigriventer* itself and two closely related species from the Ctenidae family: *Phoneutria fera* and *Cupiennius salei* (83). Therefore, we expect that the community-based efforts to annotate their genomes will provide the sequence data needed to test if oligoventin indeed maps to ctenid HECT E3 ligases.

Oligoventin synergy with C-type lysozymes suggests this HDP is a lysozyme-partner effector. When combined with lysozymes, oligoventin inhibits clinically isolated Gram-positive bacteria growth *in vitro* at concentrations as low as 11.8 µM. Thus, further research should test whether oligoventin is expressed in other tissues in addition to the eggs, which would indicate that it engages in constitutive innate immunity together with lysozymes at different developmental stages. Furthermore, the fact that synergy between antimicrobials might reduce the cost of defense (41, 59, 84) indicates that arachnids might be able to defend themselves against a wide range of pathogens from a relatively limited repertoire of host defense effectors. Synergy between two distinct classes of antimicrobials is usually explained by different modes of action between the effector molecules (41, 59, 84). Hence, lysozyme-induced bacterial peptideglycan disruption (41, 58) might facilitate oligoventin binding to its target, possibly explaining their synergy. Because oligoventin is a neutrally charged antimicrobial peptide, varying its net charge from −1 to 0.8 in pHs 10.0 and 4.0, respectively, we suggest that it is unlikely that it binds directly to anionic membranes, although it could bind to membrane receptors. However, neutrally charged peptides usually act by binding to intracellular targets such as catalytic enzymes and nucleic acids, in contrast to cationic antimicrobial peptides directly disrupting membranes (40, 85, 86). Therefore, future studies aiming to understand oligoventin’s mode of action might reveal the underlying mechanism of synergy of oligoventin with lysozymes.

Despite oligoventin’s small size, antimicrobial activity against clinical strains, synergy with lysozymes, and lack of hemolytic activity, its discovery will likely be of limited interest to drug development as many short-sized peptides such as gomesin (34) and rondonin (35), among others (84–89), outcompete oligoventin’s attractiveness as a blueprint for next-generation antimicrobial drugs. However, the discovery of oligoventin highlights the potential to screen E3s for novel peptide-based drug discovery (90). At present, the identification of oligoventin provides two main insights: first, the discovery of E3-derived peptides as a possibly new class of biologically active peptides. Second, it sheds new light on comparative immunology by illustrating a remarkable case of independent evolution of UPP function in animal host defense. Therefore, the most relevant result of our study is the evidence suggesting that E3 degradation might have been independently repurposed leading to the production of MHC class I antigens at least in humans and mouse and possibly HDPs in arachnids, respectively. Our findings indicate that the UPP was independently coupled to different immune pathways during the evolution of metazoans as a possibly convergent adaptation of metazoan immunity to produce functionally diverse peptides.

In conclusion, our data support the prediction that Nedd4s play a role in a new innate immune-related cellular pathway dependent on the UPP. The evidence presented suggests an emergent function of HECT E3s as novel precursors of HDPs in the ancient arachnid innate immune system. If confirmed, it will highlight the functional plasticity of the UPP and expand the currently known function of E3s (3, 4, 6, 7, 60, 62, 91). Thus, our results are consistent with the hypothesis that the UPP has been independently co-opted several times during evolution and gained multiple immune-related functions. Further experimentation is therefore necessary to robustly test the postulated role of Nedd4 proteins in immunity suggested by the data presented here and also to further test the precise molecular origin of oligoventin.

## Data Accessibility

The datasets supporting this article have been uploaded as part of the electronic supplementary material. The accession number for oligoventin is B3EWR9.

## Author Contributions

ILC-F, RSRS, and PIdSJ designed experiments; ILC-F, TK, RSRS, IdFCB, and PIdSJ carried out experiments; ILC-F and TK carried out bioinformatics analysis; RSRS and PIdSJ gave conceptual advice; ILC-F and PIdSJ wrote the manuscript with input from all the authors.

## Conflict of Interest Statement

The authors declare that the research was conducted in the absence of any commercial or financial relationships that could be construed as a potential conflict of interest.
