# Supplementary material for: Evidence of an Antimicrobial Peptide Signature Encrypted in HECT E3 Ubiquitin Ligases
Source: Front Immunol. 2017 Jan 9;7:664. doi: 10.3389/fimmu.2016.00664 (PMC5220581; doi:10.3389/fimmu.2016.00664)
Supplement: Supplementary file 1 [file Data_Sheet_1.doc]

**Electronic Supplementary Material**

**Evidence of an antimicrobial peptide signature encrypted in HECT E3 ubiquitin ligases**

^1,2,*^Candido-Ferreira, Ivan Lavander; ^3,4^Kronenberger, Thales; ^1,4,5^Sayegh, Raphael Santa Rosa; ^6^Batista, Isabel de Fátima Correia; ^1,*^da Silva Júnior, Pedro Ismael

1 – Laboratory of Protein Chemistry, Center of Toxins, Immune-Response and Cell Signaling, Butantan Institute, Av. Vital Brazil, 1500 - CEP 05503-900 - São Paulo, SP, Brazil.

2 – Biosciences Institute, University of São Paulo, Travessa do Matão, 14 - Butantã, 05508-090 - São Paulo, SP, Brazil.

3 – Department of Parasitology, Biomedical Science Institute, University of São Paulo, Av. Prof. Lineu Prestes, 1374 - CEP: 05508-000 - São Paulo, SP, Brazil

4 - Equal Contribution

5- Department of Biochemistry, Institute of Chemistry, University of São Paulo, Av. Prof. Lineu Prestes, 748 - 05508-000 - São Paulo - SP, Brazil.

6 – Laboratory of Biochemistry and Biophysics, Butantan Institute, Av Vital Brazil, 1500, CEP 05503-900 - São Paulo - SP, Brazil.

*to whom correspondence should be sent: [ivan.lavander.ferreira@usp.br](mailto:ivan.lavander.ferreira@usp.br), [pisjr@butantan.gov.br](mailto:pisjr@butantan.gov.br)

**
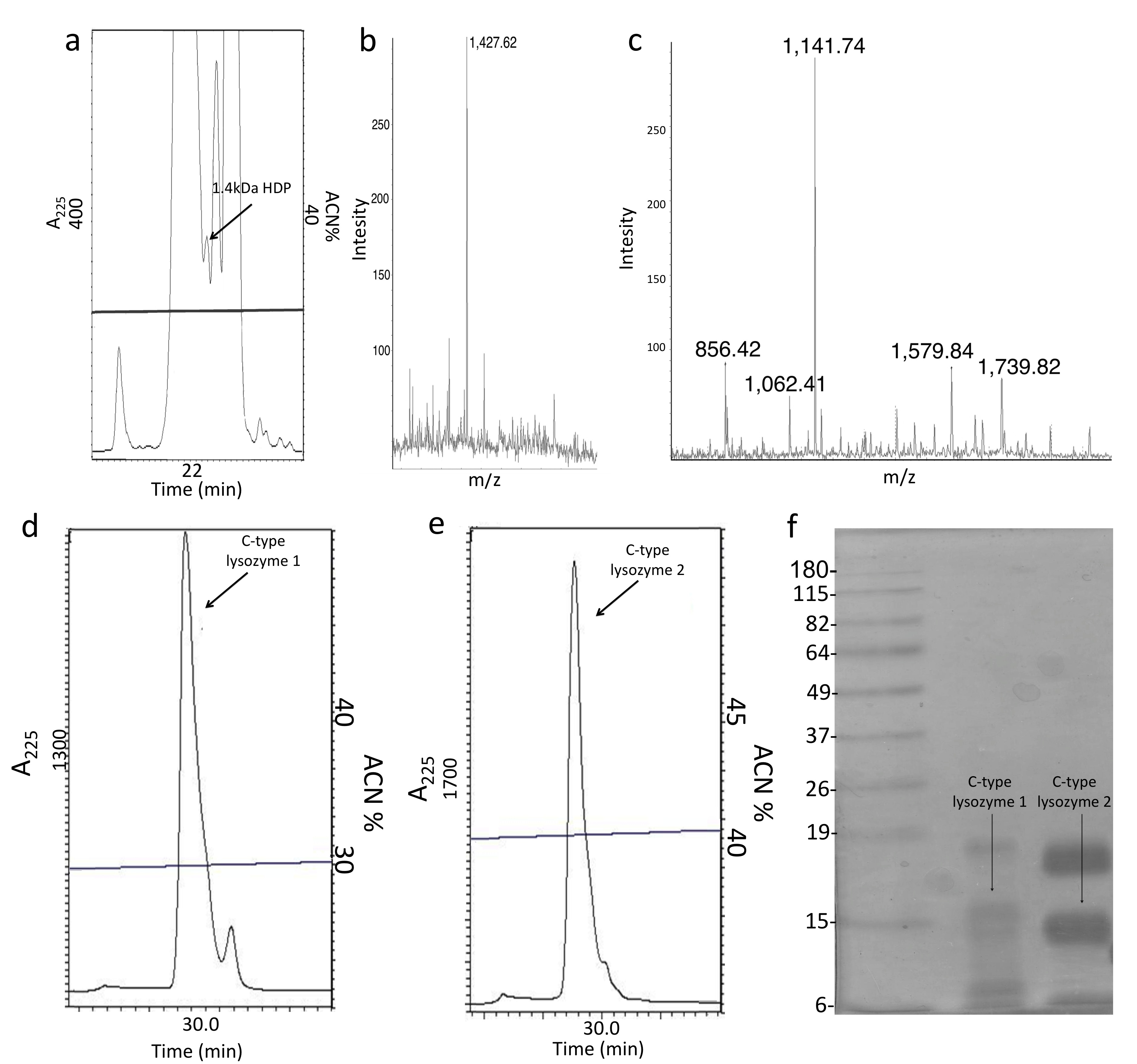
**

**Supplementary Figure S1**. Identification of *P. nigriventer* host defence effectors. (**a-b**) HPLC purification to homogeneity and MALDI spectra of a putative HDP with m/z 1,427.6. (**c**) MALDI spectra analysis of oligoventin-enriched fraction reveals co-eluted peptides ranging in size from 0.8kDa to 1.7kDa. (**d-e**) HPLC runs of isolated lysozymes. Absorbance was measured at 225 nm (A_225_). m/z, mass/charge ratio. (**f**) SDS-PAGE (12.5 % SDS-polyacrilamide gel) profile of *P. nigriventer* lysozymes. Molecular mass (in kDa) markers are indicated on the left. Proteins were stained with Coomassie blue.


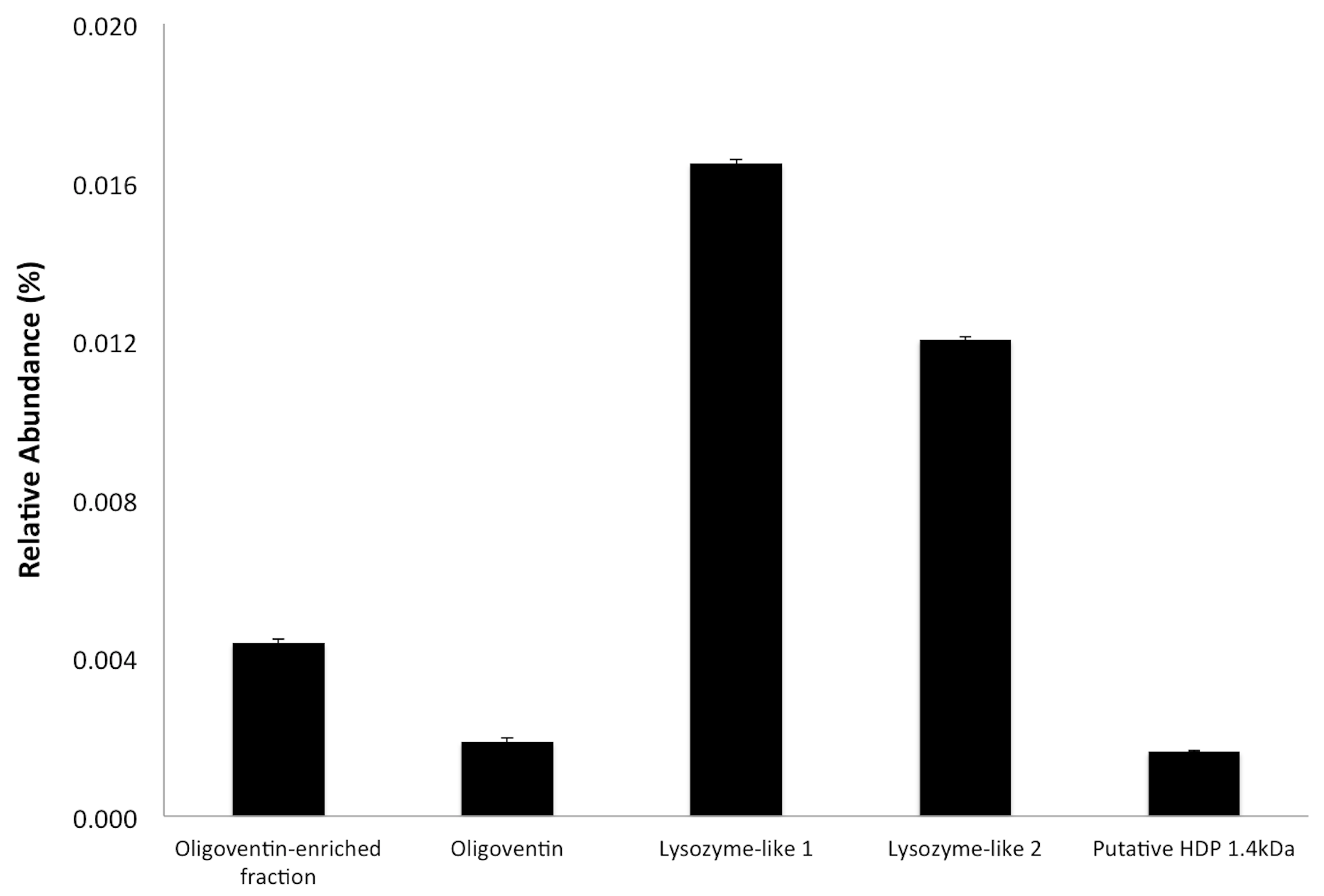


**Supplementary Figure S2.** Relative abundance of host defense effectors from *P. nigriventer* eggs. Altogether, these factors represent less than 3.2% of the total proteome at this early developmental stage of *P. nigriventer*.

**
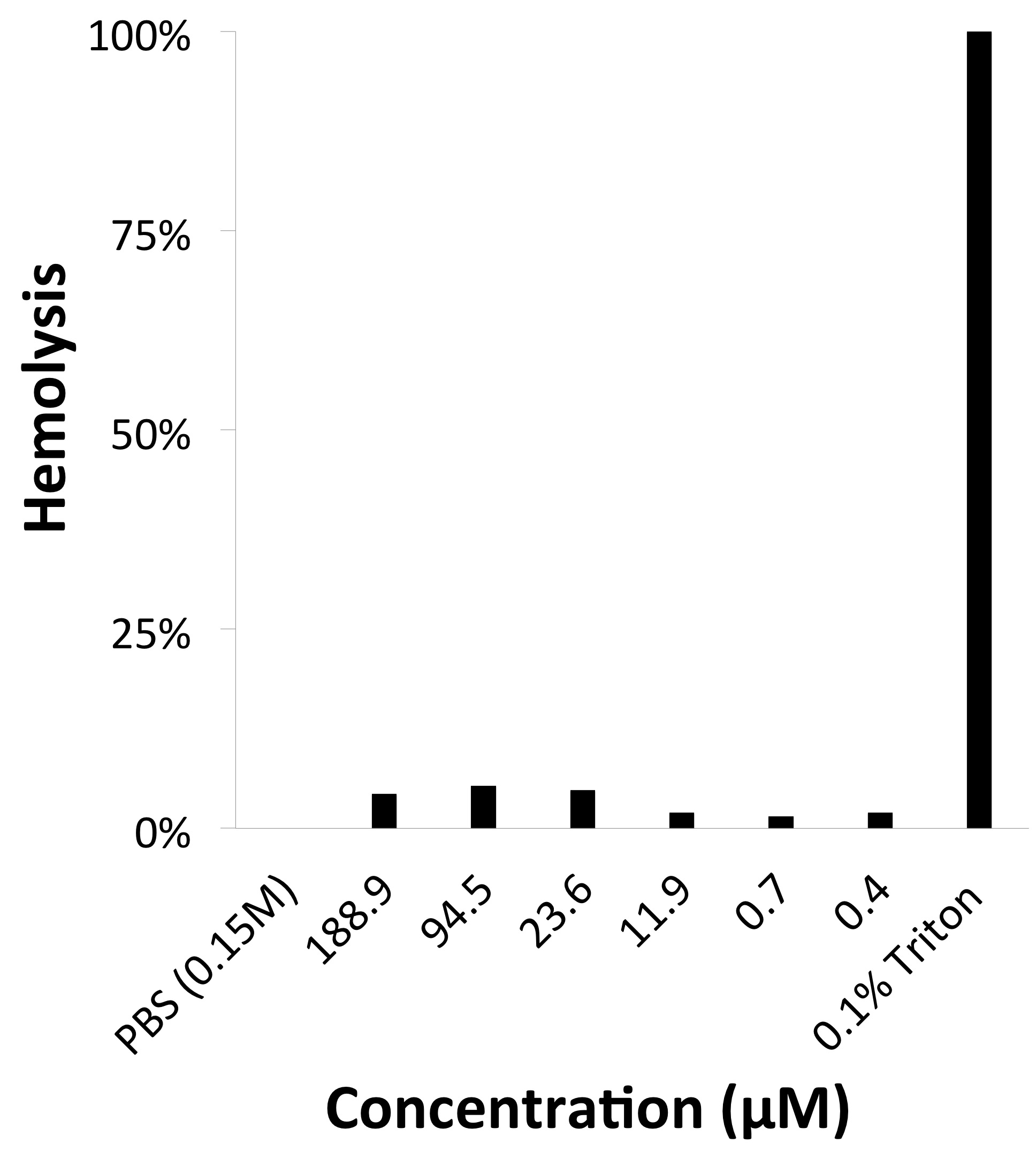
**

**Supplementary Figure S3.** Hemolytic activity of oligoventin against human erythrocytes. PBS and 1% (v/v) Triton X-100 were used as 0% and 100% controls, respectively.


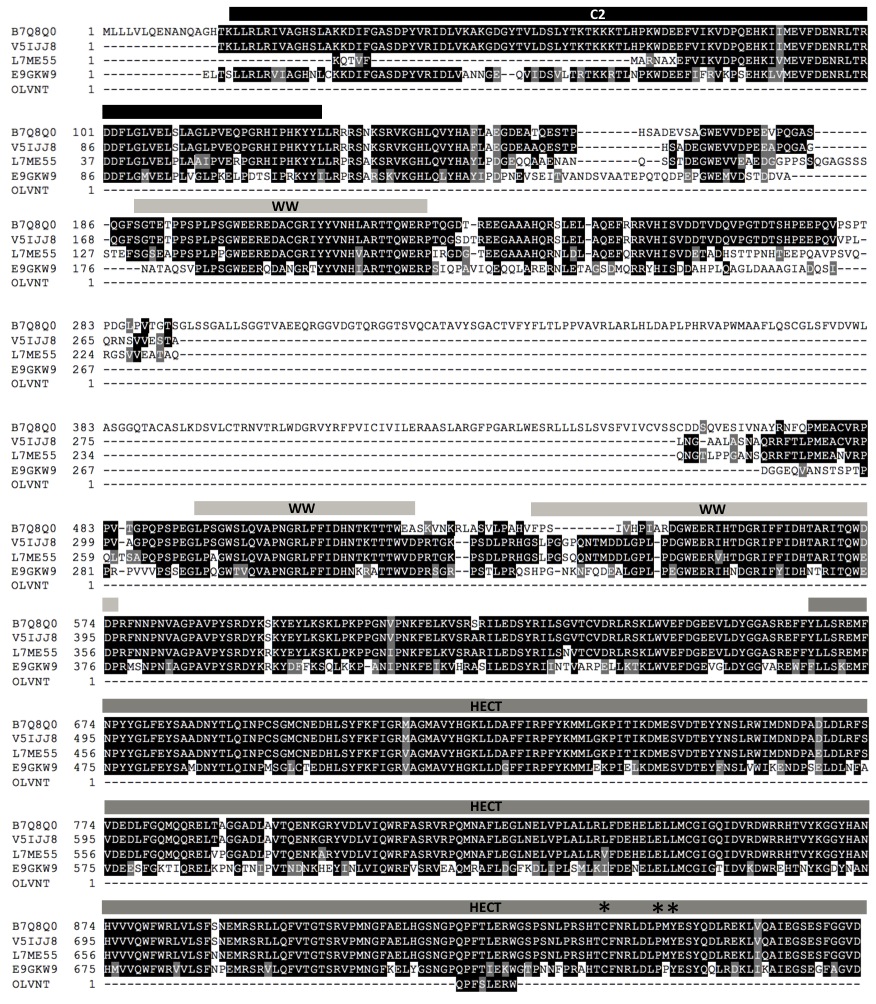


**Supplementary Figure S4.** Multiple sequence alignment (MSA) of arachnid HECT-containing Nedd4 ligases (Uniprot IDs: B7Q8Q0, V5IJJ8 AND L7ME55), the crustacean Nedd4 (E9GKW9) and oligoventin. Highly conserved amino acids are shaded in black. Bars above sequences indicate the structural C2, WW and HECT domains. Asterisks highlight the catalytic cysteine and PY motif, respectively.


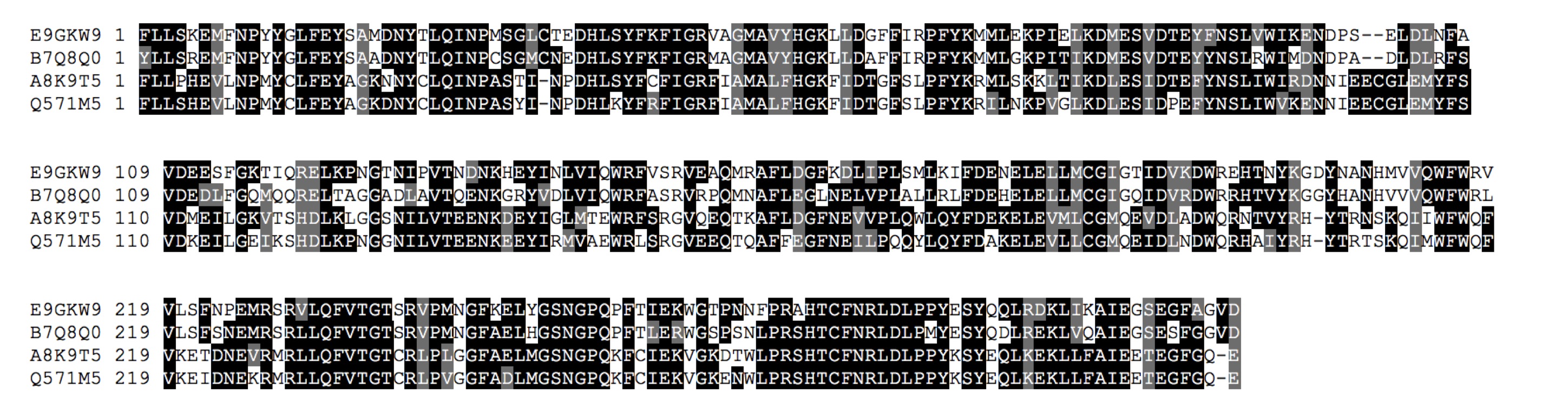


**Supplementary Figure S5.** MSA of HECT domains from vertebrate and arthropod E3s used for *in silico* protesomal degradation.


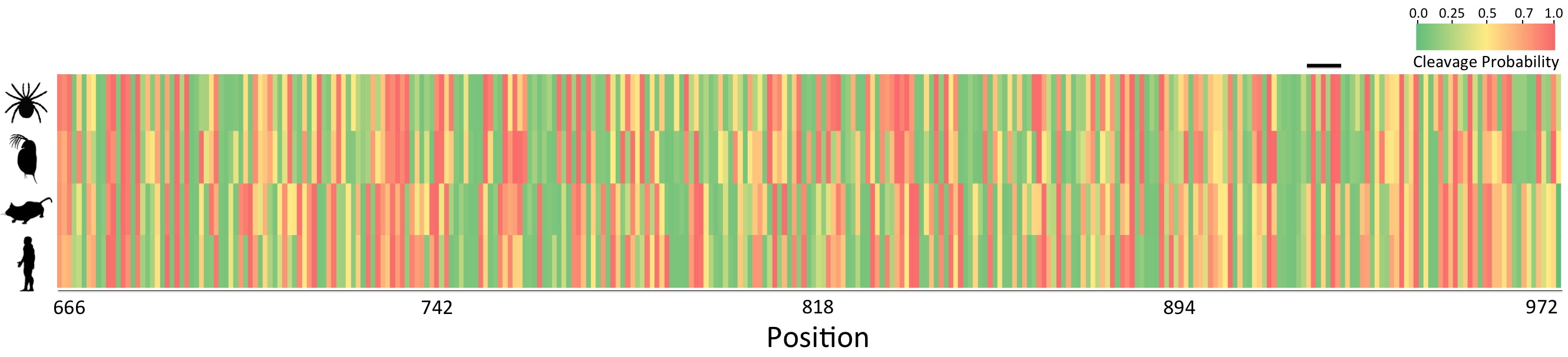


**Supplementary Figure S6.** Heat map illustrating results from *in silico* proteasomal degradation of Nedd4 HECT domains from *Ixodes scapularis*, *Daphnia pulex, Mus musculus* and *Homo sapiens*, respectively. The black bar represents the oligoventin-encrypted site. Notice the lack of cleavage sites in the flanking sides. Silhouettes from organisms are from Phylopic (phylopic.org/) and were released into the public domain.

**Supplementary Table S1 – Identification of lysozymes by MS/MS.** Results shown are a representative MS/MS ion searches against the Uniprot database using an in-house version of MASCOT Server (Matrix Science, USA). Individual ions scores > 47 indicate identity or extensive homology (p<0.05). MS/MS fragmentation spectra and corresponding tables with fragmentation masses (y series) are shown. MW, molecular weight in Da.

| **Protein ID** | **Observed MW** | **Expected MW** | **Calculated MW** | **Delta** | **Score** | **Peptide** |
| --- | --- | --- | --- | --- | --- | --- |
| **LYSC_PHAVE** | **838.89** | **1675.78** | **1675.77** | **0.0046** | **120** | **K.IVSDGDGMNAWVAWR.K** |


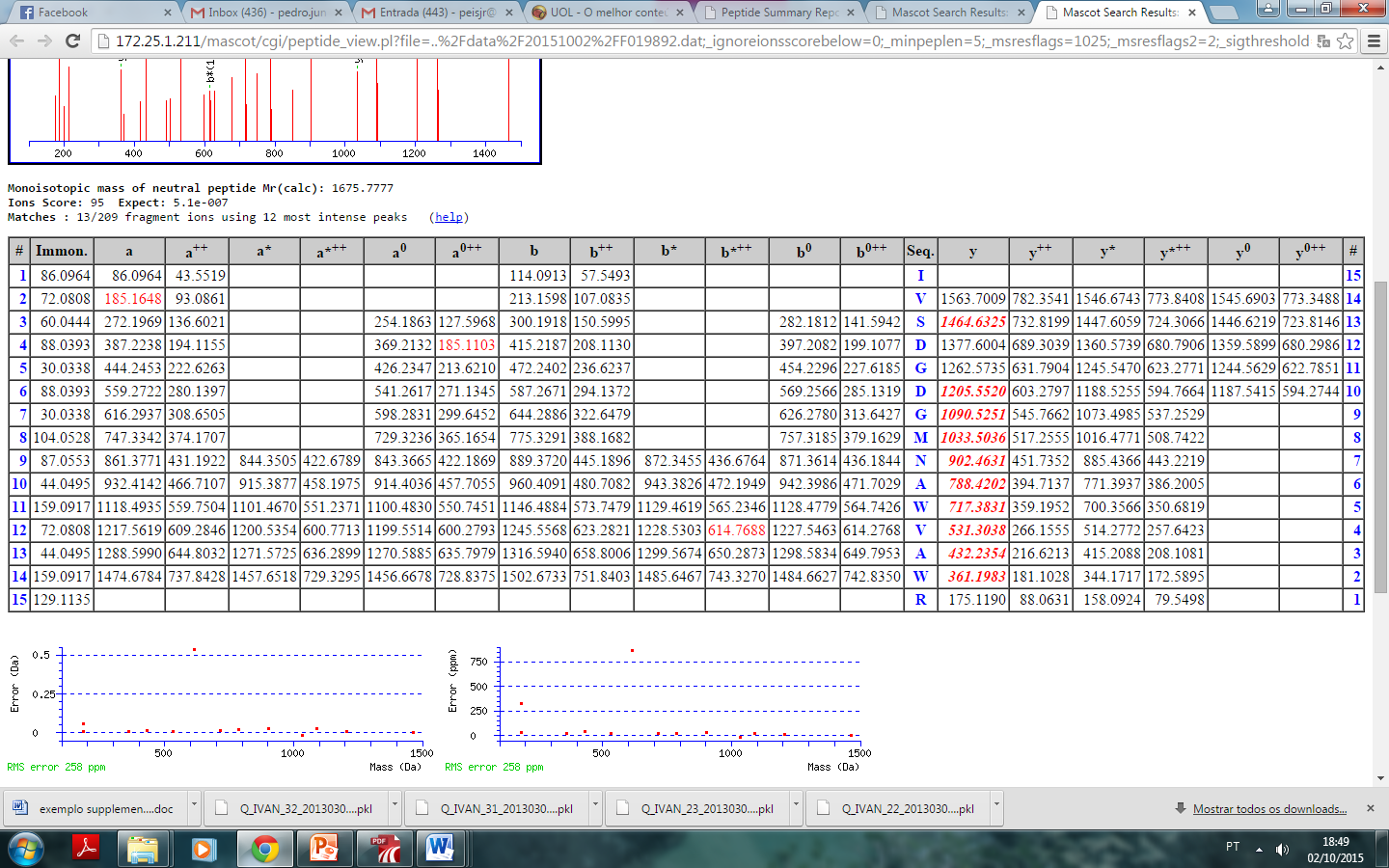

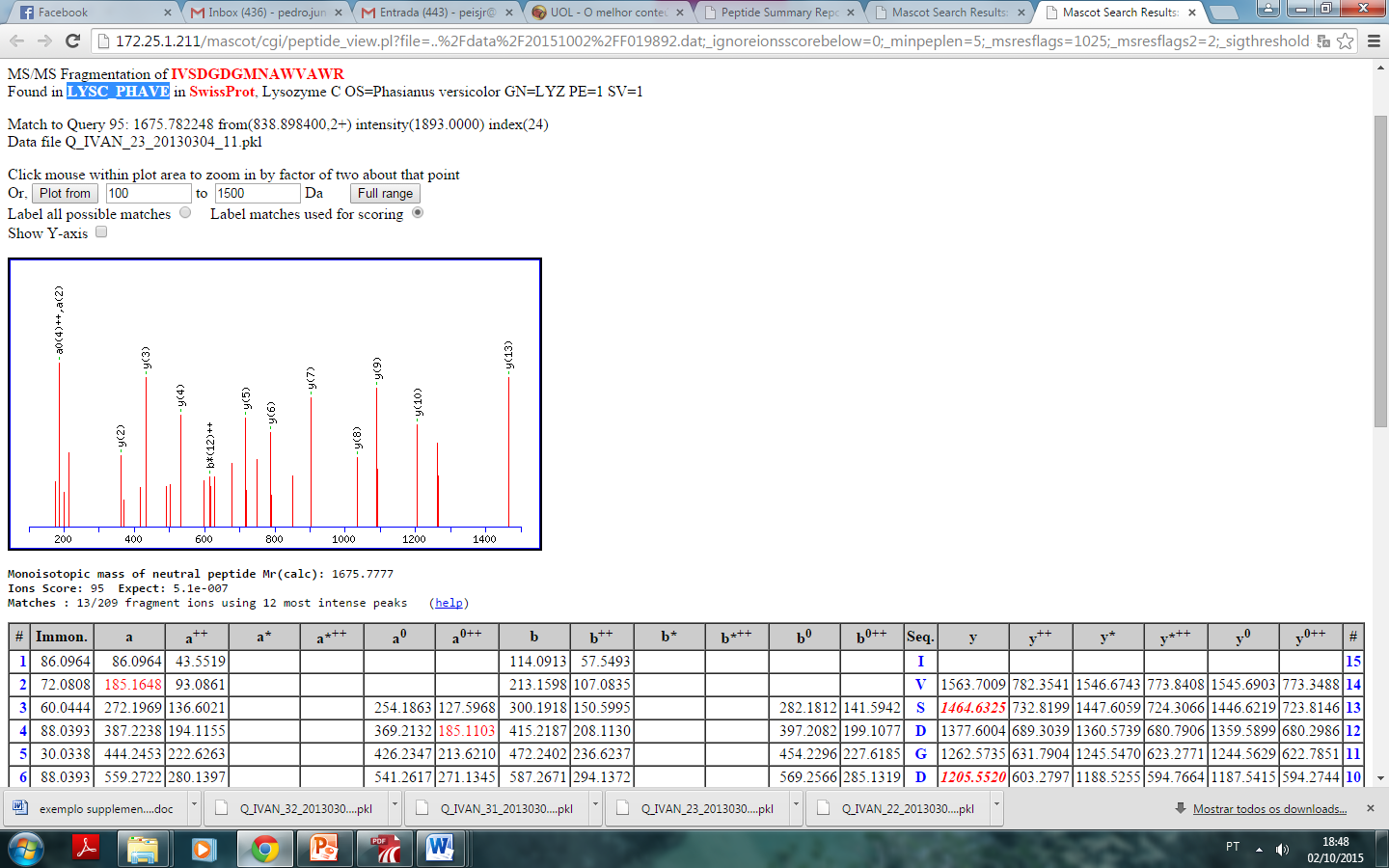


|  |  |  |  |  |  |  |  |  |  |  |  |  |  |
| --- | --- | --- | --- | --- | --- | --- | --- | --- | --- | --- | --- | --- | --- |

| **Protein ID** | **Observed MW** | **Expected MW** | **Calculated MW** | **Delta** | **Score** | **Peptide** |
| --- | --- | --- | --- | --- | --- | --- |
| **LYSC_CHICK** | **877.4202** | **1752.82** | **1752.82** | **0.0020** | **129** | **R.NTDGSTDYGILQINSR.W** |

**
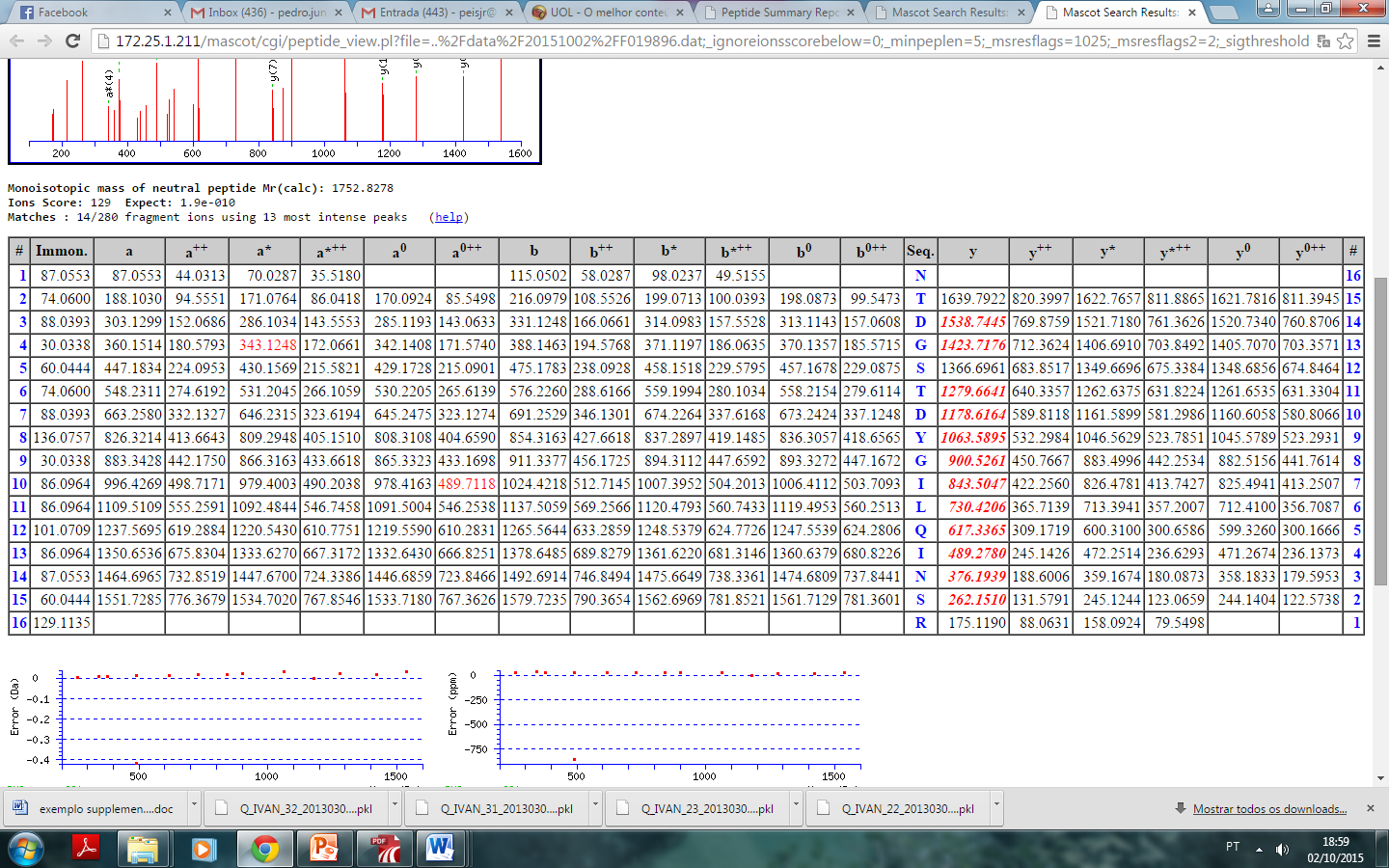

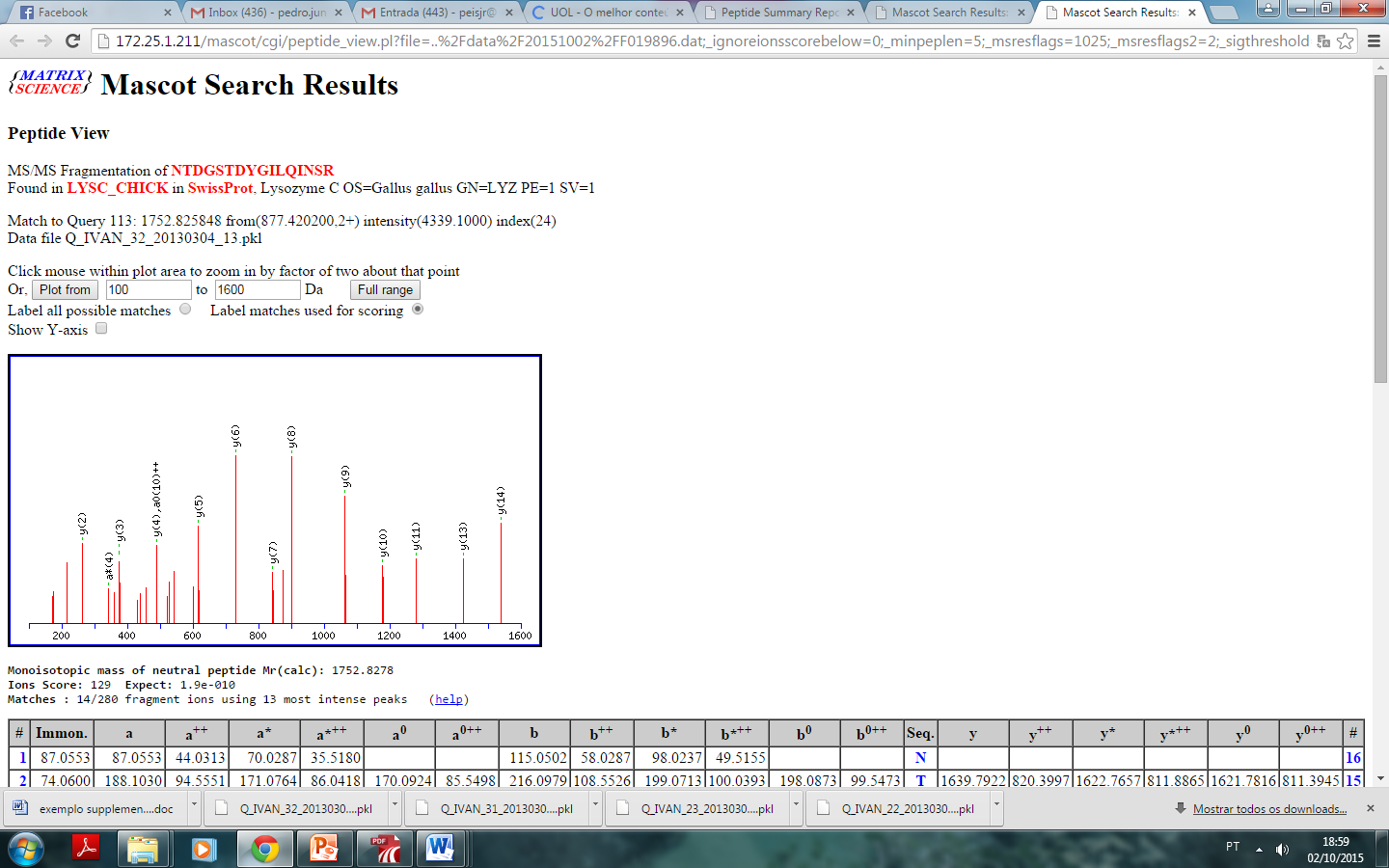
**

**Supplementary Table S2.** Anti-*M. luteus* synergy of oligoventin and hen egg lysozyme.

|  | **MIC (μM)** | | | | **Lowest FIC index^a^** |
| --- | --- | --- | --- | --- | --- |
| **Species** | Oligoventin | Hen egg lysozyme | Oligoventin in combination | Lysozyme in combination |  |
| *M. luteus A270* | 47.2-94.5 | 0.01-0.02 | 5.9-11.81 | 0.003-0.006 | 0.37(0.12+0.25) |

a- Fractional inhibitory concentration (FIC) index = FIC A + FIC B, where FIC A = [A]/MIC_A_ and FICB = [B]/MIC_B_, respectively [26]. MIC_A_ and MIC_B_ are the MICs of oligoventin and lysozymes alone and [A] and [B] are the MICs of oligoventin and lysozymes in combination. The numbers in parentheses are the FIC A and FIC B, respectively.

**Supplementary Table S3.** Homology hypotheses for oligoventin precursor proteins.

| **Description** | **Query cover** | **E value** | **Identity** | **Accession Code** |
| --- | --- | --- | --- | --- |
|  |  |  |  |  |
| Ubiquitin protein ligase, putative [Ixodes scapularis] | 1 | 0.029 | 0.88 | XP_002412402.1 |
| PREDICTED: cytochrome P450 2C23-like [Metaseiulus occidentalis] | 1 | 1.9 | 0.64 | XP_003747006.1 |
| PREDICTED: microspherule protein 1-like [Metaseiulus occidentalis] | 0.87 | 5.5 | 0.64 | XP_003741160.1 |
| PREDICTED: microspherule protein 1-like [Parasteatoda tepidariorum] | 0.87 | 5.5 | 0.64 | XP_015923740.1 |
| PREDICTED: glutamate receptor ionotropic, delta-1-like [Parasteatoda tepidariorum] | 0.87 | 5.5 | 0.86 | XP_015927423.1 |
| PREDICTED: beta-1,3-galactosyltransferase 5-like [Tetranychus urticae] | 0.75 | 5.5 | 1 | XP_015784026.1 |
| PREDICTED: E3 ubiquitin-protein ligase NEDD4-like isoform X1 [Tetranychus urticae] | 1 | 7.8 | 0.63 | XP_015784239.1 |
| PREDICTED: E3 ubiquitin-protein ligase NEDD4-like isoform X2 [Tetranychus urticae] | 1 | 7.8 | 0.63 | XP_015784241.1 |
| PREDICTED: E3 ubiquitin-protein ligase NEDD4-like isoform X3 [Tetranychus urticae] | 1 | 7.8 | 0.63 | XP_015784242.1 |
| PREDICTED: E3 ubiquitin-protein ligase NEDD4-like isoform X4 [Tetranychus urticae] | 1 | 7.8 | 0.63 | XP_015784243.1 |
| PREDICTED: E3 ubiquitin-protein ligase NEDD4-like isoform X5 [Tetranychus urticae] | 1 | 7.8 | 0.63 | XP_015784244.1 |
| PREDICTED: E3 ubiquitin-protein ligase NEDD4-like isoform X6 [Tetranychus urticae] | 1 | 7.8 | 0.63 | XP_015784245.1 |
| PREDICTED: cytochrome P450 2J2-like [Metaseiulus occidentalis] | 0.75 | 7.8 | 0.83 | XP_003748195.1 |
| alpha 1,2 mannosidase precursor-like protein [Sarcoptes scabiei] | 0.75 | 7.8 | 0.83 | KPM09352.1 |
| PREDICTED: uncharacterized protein LOC100897242 [Metaseiulus occidentalis] | 1 | 11 | 0.75 | XP_003741478.1 |
| PREDICTED: glutamate receptor ionotropic, kainate 2-like isoform X1 [Tetranychus urticae] | 0.87 | 16 | 0.86 | XP_015783967.1 |
| ionotropic glutamate receptor, putative [Ixodes scapularis] | 0.87 | 16 | 0.86 | XP_002407641.1 |
| PREDICTED: uncharacterized protein LOC107366527 [Tetranychus urticae] | 0.75 | 16 | 0.83 | XP_015789634.1 |
| PREDICTED: glutamate receptor ionotropic, kainate 2-like isoform X2 [Tetranychus urticae] | 0.87 | 16 | 0.86 | XP_015783968.1 |
| DNA polymerase alpha subunit B-like protein [Sarcoptes scabiei] | 0.87 | 16 | 0.5 | KPM08228.1 |
| PREDICTED: glutamate receptor, ionotropic kainate 3-like [Metaseiulus occidentalis] | 0.87 | 16 | 0.86 | XP_003741270.1 |
| PREDICTED: myosin-VIIa-like isoform X1 [Parasteatoda tepidariorum] | 0.62 | 22 | 1 | XP_015927846.1 |
| PREDICTED: myosin-VIIa-like isoform X2 [Parasteatoda tepidariorum] | 0.62 | 22 | 1 | XP_015927849.1 |
| Myosin-VIIa [Stegodyphus mimosarum] | 0.62 | 22 | 1 | KFM60334.1 |
| PREDICTED: lysosomal alpha-mannosidase-like [Tetranychus urticae] | 0.62 | 22 | 1 | XP_015789034.1 |
| PREDICTED: probable 3',5'-cyclic phosphodiesterase pde-5 [Parasteatoda tepidariorum] | 0.62 | 23 | 1 | XP_015921153.1 |
| sex-determining protein Fem1-like protein [Sarcoptes scabiei] | 0.62 | 23 | 1 | KPM03312.1 |
| hypothetical protein X975_14432 [Stegodyphus mimosarum] | 0.62 | 23 | 1 | KFM82786.1 |
| Cathepsin O [Stegodyphus mimosarum] | 0.62 | 23 | 1 | KFM83070.1 |
| PREDICTED: uncharacterized protein LOC107444955 isoform X1 [Parasteatoda tepidariorum] | 0.62 | 23 | 1 | XP_015914728.1 |
| PREDICTED: uncharacterized protein LOC107444955 isoform X2 [Parasteatoda tepidariorum] | 0.62 | 23 | 1 | XP_015914729.1 |
| PREDICTED: uncharacterized protein LOC107444955 isoform X3 [Parasteatoda tepidariorum] | 0.62 | 23 | 1 | XP_015914730.1 |
| PREDICTED: potassium channel subfamily K member 16-like [Parasteatoda tepidariorum] | 0.62 | 23 | 1 | XP_015923328.1 |
| PREDICTED: uncharacterized protein LOC107444955 isoform X4 [Parasteatoda tepidariorum] | 0.62 | 23 | 1 | XP_015914731.1 |
| conserved hypothetical protein [Ixodes scapularis] | 0.62 | 23 | 1 | XP_002403692.1 |
| hypothetical protein QR98_0033130 [Sarcoptes scabiei] | 0.87 | 23 | 0.71 | KPM04859.1 |
| PREDICTED: uncharacterized protein LOC107449974 [Parasteatoda tepidariorum] | 0.62 | 23 | 1 | XP_015921154.1 |
| NADH dehydrogenase subunit 3 [Steganacarus magnus] | 0.87 | 23 | 0.71 | YP_002317277.1 |
| hypothetical protein IscW_ISCW023599 [Ixodes scapularis] | 0.87 | 32 | 0.6 | XP_002416131.1 |
| PREDICTED: constitutive coactivator of peroxisome proliferator-activated receptor gamma-like [Parasteatoda tepidariorum] | 1 | 32 | 0.67 | XP_015905652.1 |
| hypothetical protein X975_22029 [Stegodyphus mimosarum] | 0.75 | 32 | 0.83 | KFM82753.1 |
| hypothetical protein QR98_0034610 [Sarcoptes scabiei] | 0.75 | 32 | 0.83 | KPM05003.1 |
| ser/thr kinase stk11, putative [Ixodes scapularis] | 1 | 32 | 0.75 | XP_002415690.1 |
| PREDICTED: twitchin-like [Parasteatoda tepidariorum] | 1 | 46 | 0.83 | XP_015925320.1 |
| Eukaryotic translation initiation factor 3 subunit A [Stegodyphus mimosarum] | 1 | 46 | 0.83 | KFM57471.1 |
| adenylyltransferase and sulfurtransferase MOCS3-like protein [Sarcoptes scabiei] | 0.75 | 46 | 0.83 | KPM06826.1 |
| WD-repeat protein, putative [Ixodes scapularis] | 0.75 | 46 | 0.83 | XP_002433401.1 |
| Major facilitator superfamily domain-containing protein 6 [Stegodyphus mimosarum] | 0.75 | 46 | 0.83 | KFM63078.1 |
| Hypothetical protein in type-1 retrotransposable element R1DM [Stegodyphus mimosarum] | 0.75 | 46 | 0.83 | KFM61453.1 |
| PREDICTED: fukutin-related protein-like [Metaseiulus occidentalis] | 0.75 | 46 | 0.83 | XP_003738081.1 |
| POC1-like protein [Sarcoptes scabiei] | 0.87 | 46 | 0.71 | KPM08764.1 |
| hypothetical protein IscW_ISCW004153 [Ixodes scapularis] | 0.75 | 47 | 0.83 | XP_002408395.1 |
| Tight junction protein ZO-1 [Stegodyphus mimosarum] | 0.87 | 65 | 0.86 | KFM71564.1 |
| PREDICTED: tight junction protein ZO-1-like [Parasteatoda tepidariorum] | 0.87 | 65 | 0.86 | XP_015917943.1 |
| PREDICTED: thyrotropin-releasing hormone-degrading ectoenzyme-like [Parasteatoda tepidariorum] | 0.75 | 65 | 0.83 | XP_015930152.1 |
| PREDICTED: glutamate receptor ionotropic, kainate 2-like [Parasteatoda tepidariorum] | 1 | 65 | 0.75 | XP_015917071.1 |
| RNA-binding protein 15B-like protein [Sarcoptes scabiei] | 0.75 | 65 | 0.83 | KPM05759.1 |
| Ubiquitin carboxyl-terminal hydrolase BAP1 [Stegodyphus mimosarum] | 0.75 | 65 | 0.83 | KFM65936.1 |
| Protein FAM149B1 [Stegodyphus mimosarum] | 0.75 | 65 | 0.83 | KFM72587.1 |
| Betaine--homocysteine S-methyltransferase 1 [Stegodyphus mimosarum] | 0.87 | 65 | 0.5 | KFM80327.1 |
| PREDICTED: transmembrane protein 161B-like [Metaseiulus occidentalis] | 0.87 | 65 | 0.83 | XP_003745243.1 |
| PREDICTED: betaine--homocysteine S-methyltransferase 1-like [Parasteatoda tepidariorum] | 0.87 | 65 | 0.5 | XP_015922930.1 |
| Neural-cadherin [Stegodyphus mimosarum] | 0.75 | 65 | 0.67 | KFM76451.1 |
| PREDICTED: lachesin-like [Metaseiulus occidentalis] | 0.75 | 65 | 0.83 | XP_003742968.1 |
| PREDICTED: alcohol dehydrogenase class-3 chain L-like isoform X1 [Parasteatoda tepidariorum] | 0.75 | 65 | 0.83 | XP_015920153.1 |
| Betaine--homocysteine S-methyltransferase 1 [Stegodyphus mimosarum] | 0.87 | 65 | 0.5 | KFM67443.1 |
| hypothetical protein X975_00770 [Stegodyphus mimosarum] | 0.75 | 65 | 0.83 | KFM67440.1 |
| PREDICTED: alcohol dehydrogenase class-3 chain L-like isoform X2 [Parasteatoda tepidariorum] | 0.75 | 65 | 0.83 | XP_015920155.1 |
| PREDICTED: uncharacterized protein LOC107363669 [Tetranychus urticae] | 0.75 | 65 | 0.83 | XP_015786419.1 |
| Phospholipase A2 [Stegodyphus mimosarum] | 0.87 | 65 | 0.39 | KFM82481.1 |
| PREDICTED: long-chain-fatty-acid--CoA ligase 6-like [Parasteatoda tepidariorum] | 0.75 | 66 | 0.83 | XP_015904674.1 |
| hypothetical protein QR98_0088520 [Sarcoptes scabiei] | 0.75 | 66 | 0.83 | KPM10299.1 |
| cellular protein AbCp-97 [Androctonus bicolor] | 0.75 | 67 | 0.83 | AIX87791.1 |
| PREDICTED: resact receptor-like [Parasteatoda tepidariorum] | 0.75 | 67 | 0.67 | XP_015927778.1 |
| cellular protein AbCp-98 [Androctonus bicolor] | 0.75 | 69 | 0.83 | AIX87792.1 |
| Uninflatable-like protein [Sarcoptes scabiei] | 0.62 | 93 | 1 | KPM04688.1 |
| PREDICTED: spectrin beta chain-like [Parasteatoda tepidariorum] | 0.62 | 93 | 1 | XP_015907082.1 |
| PREDICTED: uncharacterized protein LOC100898129 [Metaseiulus occidentalis] | 0.62 | 93 | 1 | XP_003744092.1 |
| hypothetical protein QR98_0006380 [Sarcoptes scabiei] | 0.75 | 93 | 0.67 | KPM02229.1 |
| PREDICTED: uncharacterized protein KIAA0564 homolog [Metaseiulus occidentalis] | 0.62 | 93 | 1 | XP_003737533.1 |
| hypothetical protein X975_03993 [Stegodyphus mimosarum] | 0.62 | 93 | 1 | KFM79914.1 |
| PREDICTED: Down syndrome cell adhesion molecule-like protein Dscam2 [Parasteatoda tepidariorum] | 1 | 93 | 1 | XP_015925083.1 |
| PREDICTED: mediator of RNA polymerase II transcription subunit 14-like [Metaseiulus occidentalis] | 0.62 | 93 | 1 | XP_003737168.1 |
| Multiple epidermal growth factor-like domains protein 6 [Stegodyphus mimosarum] | 0.62 | 93 | 1 | KFM77793.1 |
| PREDICTED: uncharacterized protein LOC107447624 [Parasteatoda tepidariorum] | 0.62 | 93 | 1 | XP_015918067.1 |
| PREDICTED: transcription initiation factor TFIID subunit 2 [Metaseiulus occidentalis] | 0.62 | 93 | 1 | XP_003746170.1 |
| transcription initiation factor TFII-D subunit, putative [Ixodes scapularis] | 0.75 | 93 | 1 | XP_002409520.1 |
| PREDICTED: uncharacterized protein LOC100898005 [Metaseiulus occidentalis] | 0.62 | 93 | 1 | XP_003737131.1 |
| Csa-Guanyl Cyclase 6 [Cupiennius salei] | 0.62 | 93 | 1 | CFW94181.1 |
| PREDICTED: protein LAP2-like [Tetranychus urticae] | 0.62 | 93 | 1 | XP_015784394.1 |
| PREDICTED: ADAM 17-like protease-like [Metaseiulus occidentalis] | 0.62 | 93 | 1 | XP_003737287.1 |
| PREDICTED: uncharacterized protein LOC107449900 [Parasteatoda tepidariorum] | 0.62 | 93 | 1 | XP_015921061.1 |
| PREDICTED: uncharacterized protein LOC100902626 [Metaseiulus occidentalis] | 0.62 | 93 | 1 | XP_003739958.1 |
| disintegrin and metalloproteinase domain-containing protein 12-like protein [Sarcoptes scabiei] | 0.62 | 93 | 1 | KPM10617.1 |
| PREDICTED: FTS and Hook-interacting protein-like [Parasteatoda tepidariorum] | 0.62 | 93 | 1 | XP_015909163.1 |
| Protein Jumonji [Stegodyphus mimosarum] | 0.87 | 93 | 1 | KFM64577.1 |
| PREDICTED: Bardet-Biedl syndrome 7 protein-like [Tetranychus urticae] | 0.62 | 93 | 1 | XP_015791083.1 |
| PREDICTED: nose resistant to fluoxetine protein 6-like [Metaseiulus occidentalis] | 0.62 | 93 | 1 | XP_003741622.1 |
| PREDICTED: uncharacterized protein LOC100898989 [Metaseiulus occidentalis] | 0.62 | 93 | 1 | XP_003743862.1 |
| Huntingtin [Stegodyphus mimosarum] | 0.62 | 93 | 1 | KFM68661.1 |
| zinc metalloprotease, putative [Ixodes scapularis] | 0.62 | 93 | 1 | XP_002434701.1 |
| conserved hypothetical protein [Ixodes scapularis] | 0.62 | 93 | 1 | XP_002407213.1 |
| Glycerol-3-phosphate acyltransferase 1, mitochondrial [Stegodyphus mimosarum] | 0.62 | 93 | 1 | KFM76522.1 |
| cleft lip and palate transmembrane protein 1-like protein [Sarcoptes scabiei] | 1 | 93 | 0.75 | KPM04756.1 |
| Early growth response protein 3 [Stegodyphus mimosarum] | 0.62 | 93 | 1 | KFM69900.1 |
| anaphase-promoting complex subunit, putative [Ixodes scapularis] | 0.62 | 93 | 1 | XP_002434052.1 |
| PREDICTED: desert hedgehog protein-like [Tetranychus urticae] | 0.62 | 93 | 1 | XP_015781554.1 |
| PREDICTED: transient receptor potential channel pyrexia-like [Parasteatoda tepidariorum] | 0.62 | 93 | 1 | XP_015928787.1 |
| PREDICTED: zinc finger protein 665-like [Parasteatoda tepidariorum] | 0.62 | 93 | 1 | XP_015908963.1 |
| PREDICTED: sialin-like [Tetranychus urticae] | 1 | 93 | 0.63 | XP_015783026.1 |
| PREDICTED: heterogeneous nuclear ribonucleoprotein L-like [Parasteatoda tepidariorum] | 0.62 | 93 | 1 | XP_015930931.1 |
| PREDICTED: tryptase-like [Tetranychus urticae] | 0.62 | 93 | 1 | XP_015793283.1 |
| oxidase/peroxidase-like protein [Sarcoptes scabiei] | 0.62 | 93 | 1 | KPM10364.1 |
| PREDICTED: uncharacterized protein LOC107452523 isoform X1 [Parasteatoda tepidariorum] | 0.62 | 93 | 1 | XP_015924500.1 |
| PREDICTED: uncharacterized protein LOC107452523 isoform X2 [Parasteatoda tepidariorum] | 0.62 | 93 | 1 | XP_015924501.1 |
| PREDICTED: uncharacterized protein LOC100897771 [Metaseiulus occidentalis] | 0.62 | 93 | 1 | XP_003741180.1 |
| PREDICTED: prolactin regulatory element-binding protein-like [Metaseiulus occidentalis] | 0.62 | 93 | 1 | XP_003741021.1 |
| PREDICTED: uncharacterized protein LOC107452524 [Parasteatoda tepidariorum] | 0.62 | 93 | 1 | XP_015924502.1 |
| PREDICTED: nucleoside diphosphate-linked moiety X motif 19, mitochondrial-like [Metaseiulus occidentalis] | 0.62 | 93 | 1 | XP_003737550.1 |
| PREDICTED: general transcription factor IIH subunit 4-like [Parasteatoda tepidariorum] | 0.75 | 93 | 0.67 | XP_015915442.1 |
| PREDICTED: STAM-binding protein-like A [Tetranychus urticae] | 0.62 | 93 | 1 | XP_015785965.1 |
| PREDICTED: agrin-like [Parasteatoda tepidariorum] | 0.75 | 93 | 0.83 | XP_015922150.1 |
| Guanine nucleotide-binding protein subunit alpha-13 [Stegodyphus mimosarum] | 0.62 | 93 | 1 | KFM78430.1 |
| sulfotransferase, putative [Ixodes scapularis] | 0.62 | 93 | 1 | XP_002405640.1 |
| transcription factor, putative [Ixodes scapularis] | 0.75 | 93 | 0.83 | XP_002406075.1 |
| Mg2+ and Co2+ transporter, putative [Ixodes scapularis] | 0.62 | 93 | 1 | XP_002405939.1 |
| PREDICTED: eukaryotic translation initiation factor 3 subunit H-like [Metaseiulus occidentalis] | 0.62 | 93 | 1 | XP_003737547.1 |
| PREDICTED: polymerase delta-interacting protein 2-like [Metaseiulus occidentalis] | 0.62 | 93 | 1 | XP_003742125.1 |
| PREDICTED: glutamine synthetase-like [Parasteatoda tepidariorum] | 0.62 | 93 | 1 | XP_015914943.1 |
| Homeobox-containing protein 1 [Stegodyphus mimosarum] | 0.62 | 93 | 1 | KFM82034.1 |
| PREDICTED: putative glucose-6-phosphate 1-epimerase [Parasteatoda tepidariorum] | 0.62 | 93 | 1 | XP_015929418.1 |
| hypothetical protein X975_14693 [Stegodyphus mimosarum] | 0.62 | 93 | 1 | KFM69752.1 |
| PREDICTED: uncharacterized protein C3orf38 homolog [Tetranychus urticae] | 0.62 | 93 | 1 | XP_015793509.1 |
| PREDICTED: venom protease-like [Tetranychus urticae] | 0.62 | 93 | 1 | XP_015793377.1 |
| PREDICTED: venom protease-like [Tetranychus urticae] | 0.62 | 93 | 1 | XP_015793319.1 |
| PREDICTED: uncharacterized protein LOC107456136 [Parasteatoda tepidariorum] | 0.62 | 93 | 1 | XP_015929406.1 |
| Polymerase delta-interacting protein 2 [Stegodyphus mimosarum] | 0.62 | 93 | 1 | KFM75472.1 |
| hypothetical protein QR98_0055960 [Sarcoptes scabiei] | 0.62 | 93 | 1 | KPM07112.1 |
| cytochrome c oxidase subunit II [Ricinoides karschii] | 0.62 | 93 | 1 | YP_009002085.1 |
| hypothetical protein IscW_ISCW021275 [Ixodes scapularis] | 0.87 | 94 | 0.46 | XP_002402830.1 |
| PREDICTED: Down syndrome cell adhesion molecule-like protein 1 [Parasteatoda tepidariorum] | 0.62 | 94 | 1 | XP_015910422.1 |
| hypothetical protein IscW_ISCW017514 [Ixodes scapularis] | 0.62 | 94 | 1 | XP_002407624.1 |
| TPA: translation initiation factor 2 beta subunit [Amblyomma variegatum] | 0.62 | 94 | 1 | DAA34350.1 |
| hypothetical protein IscW_ISCW015993 [Ixodes scapularis] | 0.62 | 94 | 1 | XP_002433492.1 |
| ribosomal protein L16, putative [Ixodes scapularis] | 0.75 | 94 | 0.83 | XP_002404674.1 |
| PREDICTED: agrin-like [Parasteatoda tepidariorum] | 0.75 | 94 | 0.83 | XP_015911518.1 |
| cytochrome P450, putative [Ixodes scapularis] | 0.62 | 94 | 1 | XP_002413882.1 |
| hypothetical protein QR98_0044180 [Sarcoptes scabiei] | 0.62 | 94 | 1 | KPM05945.1 |
| hypothetical protein IscW_ISCW004482 [Ixodes scapularis] | 0.62 | 94 | 1 | XP_002403502.1 |
| NADH dehydrogenase subunit 3 [Carios capensis] | 0.87 | 94 | 1 | NP_945207.1 |
| NADH dehydrogenase subunit 3 [Carios marinkellei] | 0.87 | 94 | 1 | AHF21696.1 |
| NADH dehydrogenase subunit 3 [Antricola mexicanus] | 0.87 | 94 | 1 | YP_008999622.1 |
| NADH dehydrogenase subunit 3 [Psoroptes cuniculi] | 0.62 | 94 | 1 | YP_009051470.1 |
| hypothetical protein IscW_ISCW001060 [Ixodes scapularis] | 0.62 | 95 | 1 | XP_002407842.1 |
| TPA: hypothetical secreted peptide precursor 2002 [Amblyomma variegatum] | 0.62 | 95 | 1 | DAA34671.1 |
| hypothetical protein QR98_0026680 [Sarcoptes scabiei] | 0.75 | 98 | 0.83 | KPM04225.1 |
| PREDICTED: pyruvate carboxylase, mitochondrial-like [Parasteatoda tepidariorum] | 0.75 | 132 | 0.83 | XP_015913705.1 |
| Pyruvate carboxylase, mitochondrial [Stegodyphus mimosarum] | 0.75 | 132 | 0.83 | KFM65987.1 |
| pre-mRNA-splicing factor SYF1-like protein [Sarcoptes scabiei] | 0.87 | 132 | 0.71 | KPM02154.1 |
| PREDICTED: suppressor APC domain-containing protein 2-like [Tetranychus urticae] | 0.75 | 133 | 0.83 | XP_015781886.1 |
| saposin-like protein [Sarcoptes scabiei] | 0.75 | 133 | 0.83 | KPL93614.1 |
| conserved hypothetical protein [Ixodes scapularis] | 0.87 | 133 | 0.47 | XP_002407772.1 |
| PREDICTED: probable tRNA pseudouridine synthase 2 [Parasteatoda tepidariorum] | 0.75 | 133 | 0.83 | XP_015926983.1 |
| PREDICTED: uncharacterized protein LOC107439453 [Parasteatoda tepidariorum] | 0.75 | 134 | 0.83 | XP_015907540.1 |
| PREDICTED: LOW QUALITY PROTEIN: midasin-like [Tetranychus urticae] | 0.5 | 189 | 1 | XP_015781709.1 |
| PREDICTED: dynein heavy chain 5, axonemal-like [Parasteatoda tepidariorum] | 0.75 | 189 | 1 | XP_015904465.1 |
| protocadherin-16, putative [Ixodes scapularis] | 0.87 | 189 | 0.71 | XP_002405854.1 |
| PREDICTED: teneurin-m-like isoform X1 [Parasteatoda tepidariorum] | 0.5 | 189 | 1 | XP_015918146.1 |
| PREDICTED: teneurin-m-like isoform X2 [Parasteatoda tepidariorum] | 0.5 | 189 | 1 | XP_015918147.1 |
| PREDICTED: teneurin-m-like isoform X3 [Parasteatoda tepidariorum] | 0.5 | 189 | 1 | XP_015918148.1 |
| PREDICTED: dystrophin-like [Metaseiulus occidentalis] | 0.62 | 189 | 1 | XP_003739283.1 |
| Teneurin-3 [Stegodyphus mimosarum] | 0.5 | 189 | 1 | KFM71252.1 |
| PREDICTED: teneurin-m-like isoform X4 [Parasteatoda tepidariorum] | 0.5 | 189 | 1 | XP_015918149.1 |
| hypothetical protein X975_19997 [Stegodyphus mimosarum] | 0.5 | 189 | 1 | KFM64386.1 |
| PREDICTED: pre-mRNA-processing-splicing factor 8 [Parasteatoda tepidariorum] | 0.5 | 189 | 1 | XP_015919108.1 |
| Pre-mRNA-processing-splicing factor 8 [Stegodyphus mimosarum] | 0.5 | 189 | 1 | KFM66933.1 |
| PREDICTED: pre-mRNA-processing-splicing factor 8 isoform 1 [Metaseiulus occidentalis] | 0.5 | 189 | 1 | XP_003737753.1 |
| PREDICTED: pre-mRNA-processing-splicing factor 8 [Tetranychus urticae] | 0.5 | 189 | 1 | XP_015785774.1 |
| PREDICTED: myosin-VIIa-like [Metaseiulus occidentalis] | 0.5 | 189 | 1 | XP_003741624.1 |
| pre-mRNA-processing-splicing factor 8-like protein [Sarcoptes scabiei] | 0.5 | 189 | 1 | KPM02532.1 |
| PREDICTED: pre-mRNA-processing-splicing factor 8 isoform 2 [Metaseiulus occidentalis] | 0.5 | 189 | 1 | XP_003737754.1 |
| pre-mRNA splicing factor Prp8, putative [Ixodes scapularis] | 0.5 | 189 | 1 | XP_002412787.1 |
| Spectrin beta chain [Stegodyphus mimosarum] | 0.5 | 189 | 1 | KFM62398.1 |
| phosphatidylinositol 4 kinase, putative [Ixodes scapularis] | 0.5 | 189 | 1 | XP_002400362.1 |
| PREDICTED: formin-J-like [Parasteatoda tepidariorum] | 0.5 | 189 | 1 | XP_015905823.1 |
| PREDICTED: brefeldin A-inhibited guanine nucleotide-exchange protein 2-like [Parasteatoda tepidariorum] | 0.75 | 189 | 0.83 | XP_015926421.1 |
| PREDICTED: uncharacterized protein LOC107447458 [Parasteatoda tepidariorum] | 0.75 | 189 | 0.83 | XP_015917864.1 |
| PREDICTED: CD109 antigen-like [Metaseiulus occidentalis] | 0.5 | 189 | 1 | XP_003742963.1 |
| PREDICTED: CD109 antigen-like [Parasteatoda tepidariorum] | 0.5 | 189 | 1 | XP_015919307.1 |
| macroglobulin complement-related [Hasarius adansoni] | 0.5 | 189 | 1 | BAR45624.1 |
| PREDICTED: spectrin alpha chain-like [Parasteatoda tepidariorum] | 0.5 | 189 | 1 | XP_015921115.1 |
| hypothetical protein X975_05837 [Stegodyphus mimosarum] | 0.75 | 189 | 0.83 | KFM56551.1 |
| map-kinase activating death domain protein, putative [Ixodes scapularis] | 0.5 | 189 | 1 | XP_002400464.1 |
| PREDICTED: bromodomain adjacent to zinc finger domain protein 1A-like isoform X1 [Parasteatoda tepidariorum] | 0.5 | 189 | 1 | XP_015928078.1 |
| PREDICTED: bromodomain adjacent to zinc finger domain protein 1A-like isoform X2 [Parasteatoda tepidariorum] | 0.5 | 189 | 1 | XP_015928080.1 |
| PREDICTED: protein VPRBP-like [Tetranychus urticae] | 0.5 | 189 | 1 | XP_015787303.1 |
| PREDICTED: uncharacterized protein LOC100897466 [Metaseiulus occidentalis] | 0.5 | 189 | 1 | XP_003738216.1 |
| conserved hypothetical protein [Ixodes scapularis] | 0.5 | 189 | 1 | XP_002410473.1 |
| PREDICTED: uncharacterized protein LOC100908993 [Metaseiulus occidentalis] | 0.87 | 189 | 0.71 | XP_003747825.1 |
| PREDICTED: bromodomain adjacent to zinc finger domain protein 1A-like isoform X3 [Parasteatoda tepidariorum] | 0.5 | 189 | 1 | XP_015928081.1 |
| PREDICTED: uncharacterized protein LOC107367504 isoform X1 [Tetranychus urticae] | 0.5 | 189 | 1 | XP_015790694.1 |
| PREDICTED: bromodomain adjacent to zinc finger domain protein 1A-like isoform X4 [Parasteatoda tepidariorum] | 0.5 | 189 | 1 | XP_015928082.1 |
| PREDICTED: bromodomain adjacent to zinc finger domain protein 1A-like isoform X5 [Parasteatoda tepidariorum] | 0.5 | 189 | 1 | XP_015928083.1 |
| PREDICTED: leucine-rich repeat-containing G-protein coupled receptor 4-like isoform X2 [Tetranychus urticae] | 0.5 | 189 | 1 | XP_015790702.1 |
| nuclear pore complex protein nup98, putative [Ixodes scapularis] | 0.87 | 189 | 0.67 | XP_002415833.1 |
| PREDICTED: uncharacterized protein LOC100900858 [Metaseiulus occidentalis] | 0.5 | 189 | 1 | XP_003738231.1 |
| multidrug resistance protein, putative [Ixodes scapularis] | 0.5 | 189 | 1 | XP_002410806.1 |
| PREDICTED: uncharacterized protein LOC107367281 isoform X1 [Tetranychus urticae] | 0.87 | 189 | 0.71 | XP_015790463.1 |
| PREDICTED: uncharacterized protein LOC107367281 isoform X2 [Tetranychus urticae] | 0.87 | 189 | 0.71 | XP_015790471.1 |
| PREDICTED: zinc finger SWIM domain-containing protein 4-like [Tetranychus urticae] | 0.5 | 189 | 1 | XP_015785030.1 |
| PREDICTED: lutropin-choriogonadotropic hormone receptor-like isoform X3 [Tetranychus urticae] | 0.5 | 189 | 1 | XP_015790709.1 |
| PREDICTED: protein FAM214A-like isoform X1 [Parasteatoda tepidariorum] | 0.5 | 189 | 1 | XP_015908347.1 |
| atrial natriuretic peptide receptor 1-like protein [Sarcoptes scabiei] | 0.75 | 189 | 0.86 | KPM04974.1 |
| myosin xvIII, putative [Ixodes scapularis] | 1 | 189 | 1 | XP_002434151.1 |
| PREDICTED: tonsoku-like protein-like [Metaseiulus occidentalis] | 0.5 | 189 | 1 | XP_003745847.1 |
| PREDICTED: protein FAM214A-like isoform X2 [Parasteatoda tepidariorum] | 0.5 | 189 | 1 | XP_015908348.1 |
| PREDICTED: furin-like isoform X1 [Tetranychus urticae] | 0.5 | 189 | 1 | XP_015787243.1 |
| PREDICTED: furin-like isoform X2 [Tetranychus urticae] | 0.5 | 189 | 1 | XP_015787244.1 |
| PREDICTED: unconventional myosin-IXb-like [Metaseiulus occidentalis] | 0.75 | 189 | 0.86 | XP_003739021.1 |
| exportin, putative [Ixodes scapularis] | 0.87 | 189 | 0.71 | XP_002433427.1 |
| Cullin-5 [Stegodyphus mimosarum] | 0.5 | 189 | 1 | KFM65638.1 |
| Protein FAM214A [Stegodyphus mimosarum] | 0.5 | 189 | 1 | KFM67019.1 |
| PREDICTED: putative uncharacterized protein DDB_G0286901 [Tetranychus urticae] | 0.5 | 189 | 1 | XP_015785917.1 |
| PREDICTED: thyrotropin receptor-like [Parasteatoda tepidariorum] | 0.5 | 189 | 1 | XP_015907776.1 |
| PREDICTED: glutamate receptor ionotropic, kainate 3-like [Tetranychus urticae] | 0.87 | 189 | 0.71 | XP_015781799.1 |
| PREDICTED: uncharacterized protein LOC107437838 isoform X1 [Parasteatoda tepidariorum] | 0.87 | 189 | 0.71 | XP_015905453.1 |
| PREDICTED: glycogen phosphorylase-like [Tetranychus urticae] | 0.5 | 189 | 1 | XP_015784291.1 |
| PREDICTED: protein nubbin-like isoform X1 [Tetranychus urticae] | 0.5 | 189 | 1 | XP_015785589.1 |
| hypothetical protein X975_23362 [Stegodyphus mimosarum] | 0.75 | 189 | 0.83 | KFM78005.1 |
| PREDICTED: glycogen phosphorylase-like [Parasteatoda tepidariorum] | 0.5 | 189 | 1 | XP_015913582.1 |
| PREDICTED: V-type proton ATPase 116 kDa subunit a isoform 1-like [Tetranychus urticae] | 0.75 | 189 | 0.83 | XP_015782612.1 |
| PREDICTED: glycogen phosphorylase-like isoform 1 [Metaseiulus occidentalis] | 0.5 | 189 | 1 | XP_003745573.1 |
| PREDICTED: glycogen phosphorylase-like [Parasteatoda tepidariorum] | 0.5 | 189 | 1 | XP_015917698.1 |
| hypothetical protein QR98_0018320 [Sarcoptes scabiei] | 0.5 | 189 | 1 | KPM03401.1 |
| PREDICTED: protein nubbin-like isoform X2 [Tetranychus urticae] | 0.5 | 189 | 1 | XP_015785590.1 |
| Glycogen phosphorylase [Stegodyphus mimosarum] | 0.5 | 189 | 1 | KFM72138.1 |
| PREDICTED: gamma-tubulin complex component 2-like [Tetranychus urticae] | 0.5 | 189 | 1 | XP_015792715.1 |
| glycogen phosphorylase, putative [Ixodes scapularis] | 0.5 | 189 | 1 | XP_002408095.1 |
| 5'-3' exoribonuclease 2 [Stegodyphus mimosarum] | 0.75 | 189 | 0.83 | KFM64852.1 |
| PREDICTED: glycogen phosphorylase-like isoform 2 [Metaseiulus occidentalis] | 0.5 | 189 | 1 | XP_003745574.1 |
| conserved hypothetical protein [Ixodes scapularis] | 0.75 | 189 | 0.83 | XP_002406815.1 |
| nucleolar protein c7c, putative [Ixodes scapularis] | 0.5 | 189 | 1 | XP_002412512.1 |
| PREDICTED: probable G-protein coupled receptor 158 [Parasteatoda tepidariorum] | 0.62 | 189 | 0.8 | XP_015919290.1 |
| PREDICTED: transducin beta-like protein 3-like [Metaseiulus occidentalis] | 0.5 | 189 | 1 | XP_003744313.1 |
| PREDICTED: mannosyl-oligosaccharide glucosidase-like [Tetranychus urticae] | 0.5 | 189 | 1 | XP_015781560.1 |
| PREDICTED: mushroom body large-type Kenyon cell-specific protein 1-like isoform X1 [Parasteatoda tepidariorum] | 0.75 | 189 | 0.86 | XP_015926288.1 |
| PREDICTED: mushroom body large-type Kenyon cell-specific protein 1-like isoform X2 [Parasteatoda tepidariorum] | 0.75 | 189 | 0.86 | XP_015926289.1 |
| PREDICTED: endothelin-converting enzyme 2-like [Tetranychus urticae] | 0.5 | 189 | 1 | XP_015785916.1 |
| PREDICTED: piwi-like protein 2-like [Metaseiulus occidentalis] | 0.5 | 189 | 1 | XP_003742657.1 |
| PREDICTED: pleckstrin homology domain-containing family G member 5-like isoform X2 [Parasteatoda tepidariorum] | 0.87 | 189 | 0.71 | XP_015905458.1 |
| PREDICTED: glycogen phosphorylase-like isoform 3 [Metaseiulus occidentalis] | 0.5 | 189 | 1 | XP_003745575.1 |
| hypothetical protein IscW_ISCW018372 [Ixodes scapularis] | 0.5 | 189 | 1 | XP_002409282.1 |
| PREDICTED: pleckstrin homology domain-containing family G member 7-like isoform X3 [Parasteatoda tepidariorum] | 0.87 | 189 | 0.71 | XP_015905462.1 |
| PREDICTED: uncharacterized protein LOC107445026 [Parasteatoda tepidariorum] | 0.5 | 189 | 1 | XP_015914824.1 |
| PREDICTED: uncharacterized protein LOC107450172 [Parasteatoda tepidariorum] | 0.62 | 189 | 1 | XP_015921402.1 |
| Brefeldin A-inhibited guanine nucleotide-exchange protein 1 [Stegodyphus mimosarum] | 0.75 | 189 | 0.83 | KFM66953.1 |
| hypothetical protein IscW_ISCW011594 [Ixodes scapularis] | 0.5 | 189 | 1 | XP_002411809.1 |
| xylosyltransferase I, putative [Ixodes scapularis] | 0.5 | 189 | 1 | XP_002416675.1 |
| PREDICTED: ATP-binding cassette sub-family G member 5-like isoform X1 [Tetranychus urticae] | 0.5 | 189 | 1 | XP_015787633.1 |
| heparan sulfate N-deacetylase/N-sulfotransferase, putative [Ixodes scapularis] | 0.5 | 189 | 1 | XP_002414989.1 |
| PREDICTED: peroxisomal acyl-coenzyme A oxidase 3 [Metaseiulus occidentalis] | 0.5 | 189 | 1 | XP_003737239.1 |
| PREDICTED: sodium-dependent neutral amino acid transporter B(0)AT3-like [Metaseiulus occidentalis] | 0.5 | 189 | 1 | XP_003745641.1 |
| PREDICTED: hemocyanin C chain-like [Parasteatoda tepidariorum] | 0.5 | 189 | 1 | XP_015914467.1 |
| PREDICTED: POU domain, class 6, transcription factor 1-like isoform X1 [Parasteatoda tepidariorum] | 0.5 | 189 | 1 | XP_015916995.1 |
| PREDICTED: POU domain, class 6, transcription factor 2-like isoform X2 [Parasteatoda tepidariorum] | 0.5 | 189 | 1 | XP_015917002.1 |
| RNA polymerase-associated protein RTF1-like protein [Stegodyphus mimosarum] | 0.5 | 189 | 1 | KFM70040.1 |
| PREDICTED: alpha-2 adrenergic receptor-like [Metaseiulus occidentalis] | 0.75 | 189 | 0.83 | XP_003741041.1 |
| PREDICTED: POU domain, class 6, transcription factor 2-like isoform X3 [Parasteatoda tepidariorum] | 0.5 | 189 | 1 | XP_015917003.1 |
| PREDICTED: POU domain, class 6, transcription factor 1-like isoform X4 [Parasteatoda tepidariorum] | 0.5 | 189 | 1 | XP_015917004.1 |
| PREDICTED: ATP-binding cassette sub-family G member 5-like isoform X2 [Tetranychus urticae] | 0.5 | 189 | 1 | XP_015787641.1 |
| ABC transporter sub-family G-like protein 12 [Sarcoptes scabiei] | 0.5 | 189 | 1 | KPM06683.1 |
| PREDICTED: follicle-stimulating hormone receptor-like [Metaseiulus occidentalis] | 0.5 | 189 | 1 | XP_003741293.1 |
| acyl-CoA synthetase, putative [Ixodes scapularis] | 0.5 | 189 | 1 | XP_002412456.1 |
| Glycogen phosphorylase [Stegodyphus mimosarum] | 0.5 | 189 | 1 | KFM68540.1 |
| hemocyanin subunit 3c [Pandinus imperator] | 0.5 | 189 | 1 | CAZ66714.1 |
| PREDICTED: protein phosphatase 1D-like isoform X1 [Parasteatoda tepidariorum] | 0.5 | 189 | 1 | XP_015929926.1 |
| PREDICTED: uncharacterized protein LOC107447724 isoform X1 [Parasteatoda tepidariorum] | 0.62 | 189 | 0.8 | XP_015918210.1 |
| PREDICTED: hemocyanin B chain-like [Parasteatoda tepidariorum] | 0.5 | 189 | 1 | XP_015903616.1 |
| RecName: Full=Hemocyanin C chain; Short=HcC | 0.5 | 189 | 1 | Q9NFL6.3 |
| conserved hypothetical protein [Ixodes scapularis] | 0.5 | 189 | 1 | XP_002434255.1 |
| hemocyanin subunit b [Mastigoproctus giganteus] | 0.5 | 189 | 1 | CCA94923.1 |
| hemocyanin subunit b [Euphrynichus bacillifer] | 0.5 | 189 | 1 | CCA94916.1 |
| caldesmon, putative [Ixodes scapularis] | 0.5 | 189 | 1 | XP_002435050.1 |
| conserved hypothetical protein [Ixodes scapularis] | 0.5 | 189 | 1 | XP_002406136.1 |
| PREDICTED: integrin alpha-PS1-like [Parasteatoda tepidariorum] | 0.75 | 189 | 0.86 | XP_015919929.1 |
| PREDICTED: RNA-directed DNA polymerase from mobile element jockey-like [Parasteatoda tepidariorum] | 0.5 | 189 | 1 | XP_015920822.1 |
| PREDICTED: diuretic hormone receptor-like [Metaseiulus occidentalis] | 0.5 | 189 | 1 | XP_003747782.1 |
| PREDICTED: probable protein phosphatase 2C 18 isoform X2 [Parasteatoda tepidariorum] | 0.5 | 189 | 1 | XP_015929927.1 |
| PREDICTED: dentin sialophosphoprotein-like [Tetranychus urticae] | 0.5 | 189 | 1 | XP_015785757.1 |
| PREDICTED: neurogenic locus notch homolog protein 2-like isoform X1 [Parasteatoda tepidariorum] | 0.62 | 189 | 0.8 | XP_015915696.1 |
| PREDICTED: uncharacterized protein LOC107447724 isoform X2 [Parasteatoda tepidariorum] | 0.62 | 189 | 0.8 | XP_015918211.1 |
| Nose resistant to fluoxetine protein 6 [Stegodyphus mimosarum] | 0.5 | 189 | 1 | KFM82899.1 |
| PREDICTED: fibropellin-1-like isoform X2 [Parasteatoda tepidariorum] | 0.62 | 189 | 0.8 | XP_015915697.1 |
| PREDICTED: lysosome membrane protein 2-like [Metaseiulus occidentalis] | 0.5 | 189 | 1 | XP_003748561.1 |
| PREDICTED: platelet glycoprotein 4-like [Tetranychus urticae] | 0.62 | 189 | 0.8 | XP_015782932.1 |
| hypothetical protein IscW_ISCW007346 [Ixodes scapularis] | 0.5 | 189 | 1 | XP_002406370.1 |
| PREDICTED: putative methyltransferase NSUN6 [Tetranychus urticae] | 0.5 | 189 | 1 | XP_015787895.1 |
| PREDICTED: centromere protein 3-like isoform X1 [Parasteatoda tepidariorum] | 0.5 | 189 | 1 | XP_015910397.1 |
| diuretic hormone 44 receptor GPRdih2 [Ixodes scapularis] | 0.5 | 189 | 1 | AEI25532.1 |
| PREDICTED: tigger transposable element-derived protein 6-like [Metaseiulus occidentalis] | 0.5 | 189 | 1 | XP_003737431.1 |
| pou domain transcription factor, putative [Ixodes scapularis] | 0.5 | 189 | 1 | XP_002433719.1 |
| conserved hypothetical protein [Ixodes scapularis] | 0.5 | 190 | 1 | XP_002399704.1 |
| PREDICTED: oligopeptidase A-like [Metaseiulus occidentalis] | 0.5 | 190 | 1 | XP_003744696.1 |
| RhoGEF-like protein [Sarcoptes scabiei] | 0.87 | 190 | 0.71 | KPM09865.1 |
| PREDICTED: diuretic hormone receptor-like [Parasteatoda tepidariorum] | 0.5 | 190 | 1 | XP_015924071.1 |
| PREDICTED: uncharacterized protein LOC107452959 [Parasteatoda tepidariorum] | 0.87 | 190 | 0.33 | XP_015925080.1 |
| PREDICTED: scavenger receptor class B member 1-like [Metaseiulus occidentalis] | 0.5 | 190 | 1 | XP_003744703.1 |
| PREDICTED: ankyrin repeat domain-containing protein 18B-like [Tetranychus urticae] | 0.5 | 190 | 1 | XP_015781274.1 |
| PREDICTED: putative methyltransferase NSUN6-like [Metaseiulus occidentalis] | 0.5 | 190 | 1 | XP_003739882.1 |
| cytochrome P450, putative [Ixodes scapularis] | 0.62 | 190 | 0.8 | XP_002415205.1 |
| PREDICTED: carboxypeptidase B-like [Metaseiulus occidentalis] | 0.75 | 190 | 0.86 | XP_003743256.1 |
| PREDICTED: DNA primase small subunit-like [Tetranychus urticae] | 0.5 | 190 | 1 | XP_015788735.1 |
| Serine/threonine-protein kinase greatwall [Stegodyphus mimosarum] | 0.5 | 190 | 1 | KFM73410.1 |
| corticotropin-releasing factor receptor type, putative [Ixodes scapularis] | 0.5 | 190 | 1 | XP_002403968.1 |
| inhibitor of nuclear factor kappa-B kinase alpha, putative [Ixodes scapularis] | 0.5 | 190 | 1 | XP_002410053.1 |
| Nose resistant to fluoxetine protein 6 [Stegodyphus mimosarum] | 0.5 | 190 | 1 | KFM81889.1 |
| 7 transmembrane receptor (rhodopsin family)-like protein 7 [Sarcoptes scabiei] | 0.75 | 190 | 0.83 | KPM04003.1 |
| Protein phosphatase 1D [Stegodyphus mimosarum] | 0.5 | 190 | 1 | KFM82613.1 |
| conserved hypothetical protein [Ixodes scapularis] | 0.5 | 190 | 1 | XP_002412726.1 |
| PREDICTED: synaptic vesicle 2-related protein-like [Parasteatoda tepidariorum] | 0.5 | 190 | 1 | XP_015912496.1 |
| secreted multiple inositol polyphosphate phosphatase, putative [Ixodes scapularis] | 0.5 | 190 | 1 | XP_002401241.1 |
| PREDICTED: uncharacterized protein LOC100899756 [Metaseiulus occidentalis] | 0.62 | 190 | 0.8 | XP_003743626.1 |
| Inactive phospholipase C-like protein 2 [Stegodyphus mimosarum] | 0.75 | 190 | 0.5 | KFM69436.1 |
| dihydroorotate dehydrogenase, putative [Ixodes scapularis] | 0.5 | 190 | 1 | XP_002401966.1 |
| PREDICTED: tRNA dimethylallyltransferase, mitochondrial-like [Metaseiulus occidentalis] | 0.5 | 190 | 1 | XP_003737209.1 |
| cuticle protein, putative [Ixodes scapularis] | 0.62 | 190 | 0.8 | XP_002402092.1 |
| TNF-alpha induced protein B12, putative [Ixodes scapularis] | 0.5 | 190 | 1 | XP_002435258.1 |
| ribosomal protein L31, putative [Ixodes scapularis] | 0.5 | 190 | 1 | XP_002401843.1 |
| conserved hypothetical protein [Ixodes scapularis] | 0.5 | 190 | 1 | XP_002410412.1 |
| PREDICTED: heparan sulfate glucosamine 3-O-sulfotransferase 3A1-like [Parasteatoda tepidariorum] | 0.5 | 190 | 1 | XP_015927486.1 |
| PREDICTED: immunoglobulin-binding protein 1b-like [Parasteatoda tepidariorum] | 0.5 | 190 | 1 | XP_015928483.1 |
| PREDICTED: phospholipase D LcsSicTox-betaIC1-like isoform X1 [Tetranychus urticae] | 0.75 | 190 | 0.86 | XP_015792031.1 |
| hypothetical protein IscW_ISCW024944 [Ixodes scapularis] | 0.75 | 190 | 0.86 | XP_002400275.1 |
| PREDICTED: uncharacterized protein LOC107447349 [Parasteatoda tepidariorum] | 0.5 | 190 | 1 | XP_015917719.1 |
| hypothetical protein QR98_0098000 [Sarcoptes scabiei] | 0.75 | 190 | 0.83 | KPM11230.1 |
| C1A receptor, putative [Ixodes scapularis] | 0.5 | 190 | 1 | XP_002406301.1 |
| conserved hypothetical protein [Ixodes scapularis] | 0.5 | 190 | 1 | XP_002413575.1 |
| PREDICTED: phospholipase D LcsSicTox-betaIC1-like isoform X2 [Tetranychus urticae] | 0.75 | 190 | 0.86 | XP_015792032.1 |
| Fibronectin type III domain containing protein 6 [Sarcoptes scabiei] | 0.5 | 190 | 1 | KPM09174.1 |
| hemocyanin subunit C [Latrodectus hesperus] | 0.5 | 190 | 1 | ADV40153.1 |
| PREDICTED: phospholipase D LcsSicTox-betaIC1-like isoform X3 [Tetranychus urticae] | 0.75 | 190 | 0.86 | XP_015792033.1 |
| ATP synthase gamma subunit, putative [Ixodes scapularis] | 0.75 | 190 | 0.86 | XP_002415504.1 |
| transcription termination factor, putative [Ixodes scapularis] | 0.62 | 190 | 1 | XP_002410293.1 |
| PREDICTED: diuretic hormone receptor-like [Parasteatoda tepidariorum] | 0.5 | 190 | 1 | XP_015927205.1 |
| hypothetical protein IscW_ISCW023213 [Ixodes scapularis] | 0.5 | 190 | 1 | XP_002415253.1 |
| phosphatidylserine receptor, putative [Ixodes scapularis] | 0.5 | 190 | 1 | XP_002407607.1 |
| hypothetical protein IscW_ISCW010836 [Ixodes scapularis] | 0.87 | 190 | 0.71 | XP_002404981.1 |
| chitin deacetylase 5-like protein [Sarcoptes scabiei] | 0.75 | 190 | 0.83 | KPM09918.1 |
| CD109 antigen [Stegodyphus mimosarum] | 0.5 | 190 | 1 | KFM71459.1 |
| putative juvenile hormone acid methyltransferase [Hyalomma impeltatum] | 0.5 | 190 | 1 | ACI46631.1 |
| PREDICTED: E3 ubiquitin-protein ligase MARCH2-like [Parasteatoda tepidariorum] | 0.5 | 190 | 1 | XP_015914081.1 |
| twisted gastrulation-like protein [Sarcoptes scabiei] | 0.5 | 190 | 1 | KPM10190.1 |
| PREDICTED: enolase-phosphatase E1-like [Tetranychus urticae] | 0.5 | 190 | 1 | XP_015790540.1 |
| conserved hypothetical protein [Ixodes scapularis] | 0.5 | 190 | 1 | XP_002411410.1 |
| hypothetical protein IscW_ISCW006323 [Ixodes scapularis] | 0.5 | 190 | 1 | XP_002435131.1 |
| PREDICTED: uncharacterized protein LOC100899062 [Metaseiulus occidentalis] | 0.5 | 190 | 1 | XP_003739324.1 |
| PREDICTED: protein twisted gastrulation-like [Tetranychus urticae] | 0.5 | 190 | 1 | XP_015789659.1 |
| hypothetical protein QR98_0089270 [Sarcoptes scabiei] | 0.62 | 190 | 0.8 | KPM10372.1 |
| heat shock protein 70 (HSP70)-interacting protein, putative [Ixodes scapularis] | 0.5 | 190 | 1 | XP_002408076.1 |
| diuretic hormone receptor, putative [Ixodes scapularis] | 0.5 | 190 | 1 | XP_002435658.1 |
| PREDICTED: uncharacterized protein LOC100903259 [Metaseiulus occidentalis] | 0.5 | 190 | 1 | XP_003740985.1 |
| acid methyltransferase, putative [Ixodes scapularis] | 0.5 | 190 | 1 | XP_002402714.1 |
| PREDICTED: protein RER1-like [Metaseiulus occidentalis] | 0.87 | 190 | 0.71 | XP_003746768.1 |
| mitotic spindle assembly checkpoint protein MAD2A, putative [Ixodes scapularis] | 0.5 | 190 | 1 | XP_002401838.1 |
| sarcoplasmic calcium-binding proteins I, III, and IV, putative [Ixodes scapularis] | 0.5 | 190 | 1 | XP_002434211.1 |
| PREDICTED: pre-mRNA-splicing factor SPF27-like [Tetranychus urticae] | 0.87 | 190 | 0.6 | XP_015789573.1 |
| membrane-associated RING finger containing protein, putative [Ixodes scapularis] | 0.5 | 190 | 1 | XP_002409559.1 |
| PREDICTED: uncharacterized protein LOC107454317 [Parasteatoda tepidariorum] | 0.75 | 190 | 0.83 | XP_015926960.1 |
| Mitotic spindle assembly checkpoint protein MAD2A [Stegodyphus mimosarum] | 0.5 | 190 | 1 | KFM72511.1 |
| PREDICTED: mitotic spindle assembly checkpoint protein MAD2A-like [Parasteatoda tepidariorum] | 0.5 | 190 | 1 | XP_015907907.1 |
| PREDICTED: mitotic spindle assembly checkpoint protein MAD2A-like [Metaseiulus occidentalis] | 0.5 | 190 | 1 | XP_003747559.1 |
| PREDICTED: mitotic spindle assembly checkpoint protein MAD2A-like [Metaseiulus occidentalis] | 0.5 | 190 | 1 | XP_003739485.1 |
| PREDICTED: E3 ubiquitin-protein ligase MARCH2-like [Tetranychus urticae] | 0.5 | 190 | 1 | XP_015792255.1 |
| conserved hypothetical protein [Ixodes scapularis] | 0.5 | 191 | 1 | XP_002412836.1 |
| Heparan sulfate glucosamine 3-O-sulfotransferase 2 [Stegodyphus mimosarum] | 0.5 | 191 | 1 | KFM63966.1 |
| membrane-associated RING finger containing protein, putative [Ixodes scapularis] | 0.5 | 191 | 1 | XP_002416046.1 |
| hypothetical protein IscW_ISCW016676 [Ixodes scapularis] | 0.5 | 191 | 1 | XP_002408332.1 |
| hypothetical protein IscW_ISCW018970 [Ixodes scapularis] | 0.5 | 191 | 1 | XP_002434897.1 |
| PREDICTED: FAD-linked sulfhydryl oxidase ALR-like [Metaseiulus occidentalis] | 0.5 | 191 | 1 | XP_003748213.1 |
| putative boophilin-like protein [Dermacentor variabilis] | 0.5 | 191 | 1 | ACF35510.1 |
| RecName: Full=Boophilin-G2; Flags: Precursor | 0.5 | 191 | 1 | Q8WPI3.1 |
| RecName: Full=Boophilin-H2; Flags: Precursor | 0.5 | 191 | 1 | Q8WPI2.1 |
| serine protease inhibitor, putative [Ixodes scapularis] | 0.5 | 191 | 1 | XP_002413378.1 |
| Thyrotropin receptor [Stegodyphus mimosarum] | 0.5 | 191 | 1 | KFM77161.1 |
| PREDICTED: uncharacterized protein LOC107447323 [Parasteatoda tepidariorum] | 0.5 | 191 | 1 | XP_015917682.1 |
| hypothetical protein X975_07570 [Stegodyphus mimosarum] | 0.87 | 191 | 0.71 | KFM70227.1 |
| PREDICTED: sensory neuron membrane protein 1-like [Metaseiulus occidentalis] | 0.5 | 191 | 1 | XP_003747405.1 |
| hypothetical protein [Isometrus maculatus] | 0.5 | 191 | 1 | ACD11919.1 |
| hypothetical protein X975_00009 [Stegodyphus mimosarum] | 0.5 | 191 | 1 | KFM58378.1 |
| Chain E, Thrombin-bound Boophilin Displays A Functional And Accessible Reactive-site Loop | 0.5 | 191 | 1 | 2ODY_E |
| hemocyanin subunit C [Ummidia sp. JS-2013] | 0.5 | 191 | 1 | AGL10030.1 |
| hemocyanin subunit C [Bothriocyrtum californicum] | 0.5 | 191 | 1 | AGL10028.1 |
| hemocyanin subunit C [Aphonopelma reversum] | 0.5 | 191 | 1 | AGL10027.1 |
| PREDICTED: COX assembly mitochondrial protein homolog [Parasteatoda tepidariorum] | 0.5 | 192 | 1 | XP_015904914.1 |
| RecName: Full=U5-lycotoxin-Ls1a; AltName: Full=Toxin-like structure LSTX-E1; Flags: Precursor [Lycosa singoriensis] | 0.75 | 192 | 0.83 | B6DCV0.1 |
| Transmembrane protein 19 [Stegodyphus mimosarum] | 0.5 | 192 | 1 | KFM80875.1 |
| hypothetical protein IscW_ISCW013209 [Ixodes scapularis] | 0.87 | 192 | 0.67 | XP_002414246.1 |
| leucine-rich repeat serine/threonine-protein kinase 1-like protein [Sarcoptes scabiei] | 0.75 | 270 | 0.83 | KPM05284.1 |
| PREDICTED: probable serine/threonine-protein kinase DDB_G0282963 isoform X1 [Tetranychus urticae] | 0.87 | 270 | 0.6 | XP_015795931.1 |
| PREDICTED: probable serine/threonine-protein kinase DDB_G0282963 isoform X2 [Tetranychus urticae] | 0.87 | 270 | 0.6 | XP_015795932.1 |
| PREDICTED: Down syndrome cell adhesion molecule-like protein Dscam2 isoform X2 [Parasteatoda tepidariorum] | 1 | 270 | 0.63 | XP_015919515.1 |
| PREDICTED: multidrug resistance-associated protein 4-like [Tetranychus urticae] | 0.87 | 270 | 0.71 | XP_015781064.1 |
| PREDICTED: large proline-rich protein BAG6-like [Parasteatoda tepidariorum] | 0.87 | 270 | 0.86 | XP_015913022.1 |
| plasma membrane calcium-transporting ATPase 3-like protein [Sarcoptes scabiei] | 0.87 | 270 | 0.5 | KPM11886.1 |
| PREDICTED: mitogen-activated protein kinase kinase kinase MLT-like isoform X1 [Parasteatoda tepidariorum] | 0.87 | 270 | 0.57 | XP_015903757.1 |
| hypothetical protein X975_00693 [Stegodyphus mimosarum] | 0.87 | 270 | 0.71 | KFM56880.1 |
| PREDICTED: mitogen-activated protein kinase kinase kinase MLT-like isoform X2 [Parasteatoda tepidariorum] | 0.87 | 271 | 0.57 | XP_015903763.1 |
| Mitogen-activated protein kinase kinase kinase MLT [Stegodyphus mimosarum] | 0.87 | 271 | 0.57 | KFM79473.1 |
| PREDICTED: cytochrome P450 2J2-like [Tetranychus urticae] | 0.87 | 271 | 0.41 | XP_015783165.1 |
| chitin synthase, putative [Ixodes scapularis] | 0.62 | 271 | 0.8 | XP_002402530.1 |
| PREDICTED: segment polarity protein dishevelled homolog DVL-3-like [Tetranychus urticae] | 1 | 271 | 0.63 | XP_015787348.1 |
| PREDICTED: serine/threonine-protein kinase NLK-like [Metaseiulus occidentalis] | 0.87 | 271 | 0.86 | XP_003744975.1 |
| hypothetical protein X975_06244 [Stegodyphus mimosarum] | 1 | 271 | 0.67 | KFM72467.1 |
| Paternally-expressed 3 protein [Stegodyphus mimosarum] | 0.87 | 271 | 0.71 | KFM61056.1 |
| hypothetical protein X975_23011 [Stegodyphus mimosarum] | 0.87 | 271 | 0.71 | KFM77794.1 |
| PREDICTED: uncharacterized protein LOC100905625 [Metaseiulus occidentalis] | 0.75 | 271 | 0.75 | XP_003743115.1 |
| Gem-associated protein 2 [Stegodyphus mimosarum] | 0.62 | 271 | 0.8 | KFM81586.1 |
| PREDICTED: uncharacterized protein LOC100906180 [Metaseiulus occidentalis] | 0.87 | 271 | 0.86 | XP_003748438.1 |
| conserved hypothetical protein [Ixodes scapularis] | 0.87 | 272 | 0.71 | XP_002399283.1 |
| putative secreted salivary protein [Ixodes scapularis] | 0.75 | 272 | 0.75 | AAY66762.1 |
| hypothetical protein X975_14631 [Stegodyphus mimosarum] | 0.62 | 272 | 0.8 | KFM73206.1 |
| PREDICTED: E3 ubiquitin-protein ligase HUWE1-like isoform X1 [Tetranychus urticae] | 0.75 | 386 | 0.83 | XP_015788439.1 |
| PREDICTED: E3 ubiquitin-protein ligase HUWE1-like isoform X2 [Tetranychus urticae] | 0.75 | 386 | 0.83 | XP_015788440.1 |
| PREDICTED: E3 ubiquitin-protein ligase HUWE1-like isoform X3 [Tetranychus urticae] | 0.75 | 386 | 0.83 | XP_015788441.1 |
| PREDICTED: E3 ubiquitin-protein ligase HUWE1-like isoform X4 [Tetranychus urticae] | 0.75 | 386 | 0.83 | XP_015788442.1 |
| PREDICTED: E3 ubiquitin-protein ligase HUWE1-like isoform X5 [Tetranychus urticae] | 0.75 | 386 | 0.83 | XP_015788443.1 |
| PREDICTED: E3 ubiquitin-protein ligase HUWE1-like isoform X6 [Tetranychus urticae] | 0.75 | 386 | 0.83 | XP_015788444.1 |
| PREDICTED: E3 ubiquitin-protein ligase HUWE1-like isoform X7 [Tetranychus urticae] | 0.75 | 386 | 0.83 | XP_015788446.1 |
| furry-like protein [Sarcoptes scabiei] | 0.75 | 386 | 0.83 | KPM05164.1 |
| PREDICTED: CAD protein-like [Metaseiulus occidentalis] | 0.87 | 386 | 0.71 | XP_003739459.1 |
| PREDICTED: uncharacterized protein LOC107439645 [Parasteatoda tepidariorum] | 0.75 | 386 | 0.83 | XP_015907798.1 |
| cation-independent mannose-6-phosphate receptor-like protein [Sarcoptes scabiei] | 0.75 | 386 | 0.83 | KPM03088.1 |
| PREDICTED: NACHT domain- and WD repeat-containing protein 1-like isoform X1 [Parasteatoda tepidariorum] | 0.62 | 386 | 0.8 | XP_015912568.1 |
| PREDICTED: NACHT domain- and WD repeat-containing protein 1-like isoform X2 [Parasteatoda tepidariorum] | 0.62 | 387 | 0.8 | XP_015912569.1 |
| Nesprin-2 [Stegodyphus mimosarum] | 0.62 | 387 | 0.8 | KFM65646.1 |
| PREDICTED: protein kibra-like [Parasteatoda tepidariorum] | 0.75 | 387 | 0.67 | XP_015911058.1 |
| conserved hypothetical protein [Ixodes scapularis] | 0.75 | 387 | 0.83 | XP_002408788.1 |
| PREDICTED: condensin-2 complex subunit D3-like [Metaseiulus occidentalis] | 0.62 | 387 | 0.8 | XP_003737183.1 |
| Protein kibra [Stegodyphus mimosarum] | 0.75 | 387 | 0.67 | KFM62896.1 |
| PREDICTED: liprin-beta-2-like isoform X1 [Parasteatoda tepidariorum] | 0.75 | 387 | 0.83 | XP_015930223.1 |
| hypothetical protein X975_04624 [Stegodyphus mimosarum] | 0.75 | 387 | 0.83 | KFM57501.1 |
| hypothetical protein IscW_ISCW007857 [Ixodes scapularis] | 0.75 | 387 | 0.67 | XP_002408012.1 |
| Coatomer subunit beta' [Stegodyphus mimosarum] | 0.75 | 387 | 0.71 | KFM70627.1 |
| PREDICTED: uncharacterized protein LOC107441074 [Parasteatoda tepidariorum] | 0.75 | 387 | 0.83 | XP_015909699.1 |
| putative G-protein coupled receptor 158 [Stegodyphus mimosarum] | 0.75 | 387 | 0.83 | KFM81314.1 |
| PREDICTED: DNA replication licensing factor mcm5-A-like [Parasteatoda tepidariorum] | 0.87 | 387 | 0.71 | XP_015924003.1 |
| hypothetical protein QR98_0064720 [Sarcoptes scabiei] | 0.87 | 387 | 0.67 | KPM07959.1 |
| PREDICTED: uncharacterized protein LOC107445061 isoform X1 [Parasteatoda tepidariorum] | 0.75 | 387 | 0.83 | XP_015914864.1 |
| PREDICTED: liprin-beta-1-like isoform X2 [Parasteatoda tepidariorum] | 0.75 | 387 | 0.83 | XP_015930224.1 |
| PREDICTED: enhancer of polycomb homolog 1-like [Parasteatoda tepidariorum] | 0.75 | 387 | 0.67 | XP_015922199.1 |
| PREDICTED: leishmanolysin-like peptidase isoform X2 [Parasteatoda tepidariorum] | 0.75 | 387 | 0.83 | XP_015914865.1 |
| PREDICTED: leishmanolysin-like peptidase isoform X3 [Parasteatoda tepidariorum] | 0.75 | 387 | 0.83 | XP_015914866.1 |
| PREDICTED: RNA polymerase-associated protein RTF1 homolog isoform 2 [Metaseiulus occidentalis] | 0.75 | 387 | 0.67 | XP_003744270.1 |
| PREDICTED: mediator of RNA polymerase II transcription subunit 17-like [Metaseiulus occidentalis] | 0.75 | 387 | 0.67 | XP_003746343.1 |
| PREDICTED: glutamate receptor ionotropic, kainate 2-like isoform X1 [Parasteatoda tepidariorum] | 1 | 387 | 0.75 | XP_015903253.1 |
| PREDICTED: RNA polymerase-associated protein RTF1 homolog isoform 1 [Metaseiulus occidentalis] | 0.75 | 387 | 0.67 | XP_003744269.1 |
| PREDICTED: glutamate receptor ionotropic, kainate 2-like isoform X2 [Parasteatoda tepidariorum] | 1 | 387 | 0.75 | XP_015903254.1 |
| hypothetical protein IscW_ISCW011656 [Ixodes scapularis] | 0.75 | 387 | 0.83 | XP_002412180.1 |
| PREDICTED: leishmanolysin-like peptidase isoform X4 [Parasteatoda tepidariorum] | 0.75 | 387 | 0.83 | XP_015914868.1 |
| conserved hypothetical protein [Ixodes scapularis] | 0.75 | 387 | 0.83 | XP_002406880.1 |
| PREDICTED: DEP domain-containing protein 7-like [Parasteatoda tepidariorum] | 0.75 | 387 | 0.83 | XP_015927172.1 |
| PREDICTED: cytochrome P450 2J6-like [Parasteatoda tepidariorum] | 0.75 | 387 | 0.83 | XP_015925056.1 |
| PREDICTED: cytoplasmic dynein 1 light intermediate chain 1-like [Tetranychus urticae] | 0.62 | 387 | 0.8 | XP_015792163.1 |
| PREDICTED: segment polarity protein dishevelled homolog DVL-3-like [Tetranychus urticae] | 1 | 387 | 0.63 | XP_015785753.1 |
| hypothetical protein QR98_0031250 [Sarcoptes scabiei] | 0.75 | 387 | 0.83 | KPM04674.1 |
| alpha-1,3-mannosyl-glycoprotein beta-1, 2-N-acetylglucosaminyltransferase, putative [Ixodes scapularis] | 0.75 | 387 | 0.83 | XP_002410165.1 |
| PREDICTED: enhancer of polycomb homolog 1-like [Parasteatoda tepidariorum] | 0.75 | 387 | 0.67 | XP_015907991.1 |
| alcohol dehydrogenase class-3-like protein [Sarcoptes scabiei] | 0.75 | 387 | 0.83 | KPM03549.1 |
| PREDICTED: innexin shaking-B-like [Parasteatoda tepidariorum] | 0.75 | 387 | 0.83 | XP_015916628.1 |
| PREDICTED: GMP reductase 2-like [Parasteatoda tepidariorum] | 0.75 | 387 | 0.67 | XP_015916263.1 |
| hypothetical protein [Latrodectus hesperus] | 0.75 | 388 | 0.43 | ADV40304.1 |
| Enhancer of polycomb-like protein [Stegodyphus mimosarum] | 0.75 | 388 | 0.67 | KFM71369.1 |
| PREDICTED: uncharacterized protein LOC107455615 [Parasteatoda tepidariorum] | 0.75 | 388 | 0.67 | XP_015928725.1 |
| PREDICTED: DEP domain-containing protein 7-like [Parasteatoda tepidariorum] | 0.75 | 388 | 0.83 | XP_015928447.1 |
| PREDICTED: GMP reductase 2-like [Parasteatoda tepidariorum] | 0.75 | 388 | 0.67 | XP_015909990.1 |
| Octopamine receptor [Stegodyphus mimosarum] | 0.75 | 388 | 0.83 | KFM70108.1 |
| PSP domain containing protein [Sarcoptes scabiei] | 0.62 | 388 | 0.8 | KPM07551.1 |
| Abhydrolase domain-containing protein 4 [Stegodyphus mimosarum] | 0.62 | 388 | 0.8 | KFM62763.1 |
| PREDICTED: uncharacterized protein LOC100904256 [Metaseiulus occidentalis] | 0.87 | 388 | 0.75 | XP_003739288.1 |
| PREDICTED: astacin-like metalloprotease toxin 5 [Parasteatoda tepidariorum] | 0.75 | 388 | 0.83 | XP_015909666.1 |
| nudt9 protein, putative [Ixodes scapularis] | 0.75 | 388 | 0.43 | XP_002411962.1 |
| hypothetical protein IscW_ISCW020897 [Ixodes scapularis] | 0.87 | 388 | 0.46 | XP_002400862.1 |
| anaphase-promoting complex, subunit, putative [Ixodes scapularis] | 0.75 | 389 | 0.83 | XP_002402817.1 |
| protein kinase [Prokoenenia wheeleri] | 0.62 | 389 | 0.8 | ACY43495.1 |
| protein kinase [Dinothrombium pandorae] | 0.62 | 389 | 0.8 | ACY43467.1 |
| NADH dehydrogenase subunit 3 [Amblyomma americanum] | 0.75 | 390 | 0.83 | ABA19097.1 |
| NADH dehydrogenase subunit 3 [Argas africolumbae] | 0.75 | 390 | 0.83 | YP_007026374.1 |
| NADH dehydrogenase subunit 3 [Rhipicephalus sanguineus] | 0.75 | 390 | 0.83 | NP_008516.1 |
| NADH dehydrogenase subunit 3 [Rhipicephalus sanguineus] | 0.75 | 390 | 0.83 | AFR24935.1 |
| NADH dehydrogenase subunit 3 [Rhipicephalus microplus] | 0.75 | 390 | 0.83 | AID18700.1 |
| NADH dehydrogenase subunit 3 [Rhipicephalus geigyi] | 0.75 | 390 | 0.83 | YP_008999755.1 |
| NADH dehydrogenase subunit 3 [Rhipicephalus kohlsi] | 0.75 | 390 | 0.83 | AGH19752.1 |
| NADH dehydrogenase subunit 3 [Rhipicephalus microplus] | 0.75 | 390 | 0.83 | YP_008999532.1 |
| NADH dehydrogenase subunit 3 [Rhipicephalus microplus] | 0.75 | 390 | 0.83 | AGH19725.1 |
| NADH dehydrogenase subunit 3 [Rhipicephalus microplus] | 0.75 | 390 | 0.83 | AGH19712.1 |
| NADH dehydrogenase subunit 3 [Rhipicephalus appendiculatus] | 0.75 | 390 | 0.83 | AGH19691.1 |
| NADH dehydrogenase subunit 3 [Rhipicephalus annulatus] | 0.75 | 390 | 0.83 | AGH19682.1 |
| NADH dehydrogenase subunit 3 [Rhipicephalus australis] | 0.75 | 390 | 0.83 | YP_008999729.1 |
| NADH dehydrogenase subunit 3 [Ornithodoros rostratus] | 0.75 | 390 | 0.83 | YP_009000457.1 |

**Supplementary Table S4. Results for *in silico* proteasomal degradation of eight HECT-domain sequences from Nedd4s from five arachnids, one crustacean and two vertebrates using two different algorithms. Results in red highlight the oligoventin-encrypted site.**

| **Protein Template** | L7ME55 | | L7ML19 | | B7Q8Q0 | | V5IJJ8 | | T1K985 | | E9GKW9 | | A8K9T5 | | Q571M5 | |
| --- | --- | --- | --- | --- | --- | --- | --- | --- | --- | --- | --- | --- | --- | --- | --- | --- |
| **Species** | *R. pulchellus* | | *R. pulchellus* | | *I. scapularis* | | *I. ricinus* | | *T. urticae* | | *D. pulex* | | *M. musculus* | | *H. sapiens* | |
| **Algorithm** | C-term | 20S | C-term | 20S | C-term | 20S | C-term | 20S | C-term | 20S | C-term | 20S | C-term | 20S | C-term | 20S |
| **Position** | **Cleavage Probability** | | | | | | | | | | | | | | | |
| **666** | 0.86 | 0.48 | 0.86 | 0.48 | 0.86 | 0.48 | 0.86 | 0.48 | 0.86 | 0.48 | 0.67 | 0.48 | 0.70 | 0.48 | 0.66 | 0.45 |
| **667** | 0.78 | 0.54 | 0.78 | 0.54 | 0.78 | 0.54 | 0.78 | 0.54 | 0.76 | 0.33 | 0.55 | 0.26 | 0.59 | 0.50 | 0.48 | 0.51 |
| **668** | 0.92 | 0.73 | 0.92 | 0.73 | 0.92 | 0.73 | 0.92 | 0.73 | 0.89 | 0.38 | 0.86 | 0.37 | 0.80 | 0.97 | 0.52 | 0.94 |
| **669** | 0.03 | 0.53 | 0.03 | 0.53 | 0.03 | 0.53 | 0.03 | 0.53 | 0.03 | 0.38 | 0.04 | 0.40 | 0.08 | 0.35 | 0.09 | 0.08 |
| **670** | 0.35 | 0.94 | 0.35 | 0.94 | 0.35 | 0.94 | 0.35 | 0.94 | 0.73 | 0.43 | 0.77 | 0.43 | 0.15 | 0.80 | 0.10 | 0.09 |
| **671** | 0.02 | 0.91 | 0.02 | 0.91 | 0.02 | 0.91 | 0.02 | 0.91 | 0.02 | 0.88 | 0.03 | 0.88 | 0.02 | 0.24 | 0.03 | 0.20 |
| **672** | 0.30 | 0.83 | 0.30 | 0.83 | 0.30 | 0.83 | 0.30 | 0.83 | 0.40 | 0.84 | 0.46 | 0.84 | 0.59 | 0.68 | 0.48 | 0.86 |
| **673** | 0.17 | 0.92 | 0.17 | 0.92 | 0.17 | 0.92 | 0.17 | 0.92 | 0.17 | 0.88 | 0.09 | 0.88 | 0.40 | 0.54 | 0.58 | 0.54 |
| **674** | 0.02 | 0.66 | 0.02 | 0.66 | 0.02 | 0.66 | 0.02 | 0.66 | 0.02 | 0.66 | 0.02 | 0.66 | 0.02 | 0.47 | 0.03 | 0.47 |
| **675** | 0.03 | 0.31 | 0.03 | 0.31 | 0.03 | 0.31 | 0.03 | 0.31 | 0.03 | 0.31 | 0.03 | 0.31 | 0.04 | 0.36 | 0.05 | 0.36 |
| **676** | 0.82 | 0.18 | 0.82 | 0.18 | 0.82 | 0.18 | 0.82 | 0.18 | 0.88 | 0.18 | 0.88 | 0.18 | 0.74 | 0.10 | 0.95 | 0.10 |
| **677** | 0.95 | 0.72 | 0.95 | 0.72 | 0.95 | 0.72 | 0.95 | 0.72 | 0.83 | 0.72 | 0.74 | 0.72 | 0.95 | 0.82 | 0.92 | 0.82 |
| **678** | 0.03 | 0.86 | 0.03 | 0.86 | 0.03 | 0.86 | 0.03 | 0.86 | 0.04 | 0.86 | 0.04 | 0.86 | 0.03 | 0.96 | 0.03 | 0.96 |
| **679** | 0.97 | 0.87 | 0.97 | 0.87 | 0.97 | 0.87 | 0.97 | 0.87 | 0.97 | 0.87 | 0.97 | 0.87 | 0.95 | 0.95 | 0.92 | 0.95 |
| **680** | 0.87 | 0.74 | 0.87 | 0.74 | 0.87 | 0.74 | 0.87 | 0.74 | 0.87 | 0.74 | 0.85 | 0.74 | 0.86 | 0.88 | 0.73 | 0.88 |
| **681** | 0.02 | 0.63 | 0.02 | 0.63 | 0.02 | 0.63 | 0.02 | 0.63 | 0.02 | 0.63 | 0.02 | 0.63 | 0.03 | 0.71 | 0.02 | 0.71 |
| **682** | 0.98 | 0.87 | 0.98 | 0.87 | 0.98 | 0.87 | 0.98 | 0.87 | 0.98 | 0.87 | 0.97 | 0.94 | 0.92 | 0.92 | 0.83 | 0.92 |
| **683** | 0.08 | 0.31 | 0.08 | 0.31 | 0.08 | 0.31 | 0.08 | 0.31 | 0.08 | 0.31 | 0.06 | 0.45 | 0.11 | 0.08 | 0.07 | 0.09 |
| **684** | 0.47 | 0.85 | 0.47 | 0.85 | 0.47 | 0.85 | 0.47 | 0.85 | 0.47 | 0.85 | 0.31 | 0.87 | 0.03 | 0.17 | 0.03 | 0.35 |
| **685** | 0.04 | 0.78 | 0.04 | 0.78 | 0.04 | 0.78 | 0.04 | 0.78 | 0.04 | 0.78 | 0.16 | 0.89 | 0.08 | 0.40 | 0.08 | 0.62 |
| **686** | 0.55 | 0.81 | 0.55 | 0.81 | 0.55 | 0.81 | 0.55 | 0.81 | 0.55 | 0.81 | 0.60 | 0.91 | 0.29 | 0.71 | 0.24 | 0.16 |
| **687** | 0.02 | 0.24 | 0.02 | 0.24 | 0.02 | 0.24 | 0.02 | 0.24 | 0.02 | 0.24 | 0.02 | 0.51 | 0.02 | 0.43 | 0.02 | 0.76 |
| **688** | 0.60 | 0.92 | 0.60 | 0.92 | 0.60 | 0.92 | 0.60 | 0.92 | 0.58 | 0.92 | 0.53 | 0.94 | 0.85 | 0.79 | 0.97 | 0.79 |
| **689** | 0.03 | 0.09 | 0.03 | 0.09 | 0.03 | 0.09 | 0.03 | 0.09 | 0.03 | 0.09 | 0.03 | 0.09 | 0.03 | 0.21 | 0.03 | 0.32 |
| **690** | 0.97 | 0.93 | 0.97 | 0.93 | 0.97 | 0.93 | 0.97 | 0.93 | 0.97 | 0.93 | 0.95 | 0.93 | 0.93 | 0.94 | 0.95 | 0.94 |
| **691** | 0.08 | 0.63 | 0.08 | 0.63 | 0.08 | 0.63 | 0.08 | 0.63 | 0.07 | 0.63 | 0.06 | 0.63 | 0.03 | 0.64 | 0.03 | 0.64 |
| **692** | 0.97 | 0.85 | 0.97 | 0.85 | 0.97 | 0.85 | 0.97 | 0.85 | 0.96 | 0.80 | 0.95 | 0.90 | 0.86 | 0.57 | 0.86 | 0.57 |
| **693** | 0.02 | 0.14 | 0.02 | 0.14 | 0.02 | 0.14 | 0.02 | 0.14 | 0.02 | 0.37 | 0.02 | 0.17 | 0.02 | 0.09 | 0.02 | 0.09 |
| **694** | 0.04 | 0.06 | 0.04 | 0.06 | 0.04 | 0.06 | 0.04 | 0.06 | 0.04 | 0.06 | 0.03 | 0.08 | 0.03 | 0.14 | 0.03 | 0.07 |
| **695** | 0.08 | 0.06 | 0.08 | 0.06 | 0.08 | 0.06 | 0.08 | 0.06 | 0.08 | 0.04 | 0.95 | 0.17 | 0.23 | 0.08 | 0.36 | 0.07 |
| **696** | 0.08 | 0.10 | 0.08 | 0.10 | 0.08 | 0.10 | 0.08 | 0.10 | 0.09 | 0.11 | 0.14 | 0.14 | 0.04 | 0.13 | 0.04 | 0.14 |
| **697** | 0.25 | 0.70 | 0.25 | 0.70 | 0.25 | 0.70 | 0.25 | 0.70 | 0.57 | 0.57 | 0.42 | 0.51 | 0.89 | 0.94 | 0.04 | 0.43 |
| **698** | 0.80 | 0.68 | 0.80 | 0.68 | 0.80 | 0.68 | 0.80 | 0.68 | 0.87 | 0.20 | 0.91 | 0.81 | 0.56 | 0.34 | 0.90 | 0.36 |
| **699** | 0.04 | 0.19 | 0.04 | 0.19 | 0.04 | 0.19 | 0.04 | 0.19 | 0.10 | 0.22 | 0.04 | 0.13 | 0.03 | 0.54 | 0.03 | 0.40 |
| **700** | 0.03 | 0.70 | 0.03 | 0.70 | 0.03 | 0.70 | 0.03 | 0.70 | 0.03 | 0.73 | 0.03 | 0.75 | 0.02 | 0.40 | 0.02 | 0.45 |
|  |  |  |  |  |  |  |  |  |  |  |  |  |  |  |  |  |

| **701** | 0.03 | 0.97 | 0.03 | 0.97 | 0.03 | 0.97 | 0.03 | 0.97 | 0.04 | 0.96 | 0.05 | 0.95 | 0.15 | 0.05 | 0.15 | 0.06 |
| --- | --- | --- | --- | --- | --- | --- | --- | --- | --- | --- | --- | --- | --- | --- | --- | --- |
| **702** | 0.04 | 0.51 | 0.04 | 0.51 | 0.04 | 0.51 | 0.04 | 0.51 | 0.04 | 0.49 | 0.14 | 0.64 | 0.03 | 0.05 | 0.04 | 0.09 |
| **703** | 0.02 | 0.11 | 0.02 | 0.11 | 0.02 | 0.11 | 0.02 | 0.11 | 0.02 | 0.16 | 0.03 | 0.20 | 0.77 | 0.95 | 0.67 | 0.96 |
| **704** | 0.25 | 0.84 | 0.25 | 0.84 | 0.25 | 0.84 | 0.25 | 0.84 | 0.20 | 0.84 | 0.34 | 0.84 | 0.85 | 0.30 | 0.04 | 0.40 |
| **705** | 0.03 | 0.21 | 0.03 | 0.21 | 0.03 | 0.21 | 0.03 | 0.21 | 0.03 | 0.21 | 0.04 | 0.21 | 0.96 | 0.64 | 0.96 | 0.82 |
| **706** | 0.70 | 0.25 | 0.70 | 0.25 | 0.73 | 0.25 | 0.73 | 0.25 | 0.77 | 0.25 | 0.76 | 0.25 | 0.35 | 0.99 | 0.62 | 0.96 |
| **707** | 0.17 | 0.97 | 0.17 | 0.97 | 0.23 | 0.97 | 0.23 | 0.97 | 0.32 | 0.97 | 0.32 | 0.97 | 0.15 | 0.90 | 0.03 | 0.71 |
| **708** | 0.39 | 0.32 | 0.39 | 0.32 | 0.39 | 0.32 | 0.39 | 0.32 | 0.68 | 0.32 | 0.41 | 0.32 | 0.53 | 0.91 | 0.31 | 0.93 |
| **709** | 0.56 | 0.90 | 0.56 | 0.90 | 0.71 | 0.90 | 0.71 | 0.90 | 0.57 | 0.90 | 0.56 | 0.90 | 0.07 | 0.62 | 0.08 | 0.53 |
| **710** | 0.16 | 0.61 | 0.16 | 0.61 | 0.10 | 0.70 | 0.10 | 0.70 | 0.09 | 0.71 | 0.16 | 0.61 | 0.03 | 0.91 | 0.04 | 0.85 |
| **711** | 0.04 | 0.32 | 0.04 | 0.32 | 0.04 | 0.38 | 0.04 | 0.38 | 0.04 | 0.46 | 0.04 | 0.32 | 0.21 | 0.87 | 0.13 | 0.87 |
| **712** | 0.14 | 0.83 | 0.14 | 0.83 | 0.24 | 0.95 | 0.24 | 0.95 | 0.11 | 0.83 | 0.14 | 0.83 | 0.73 | 0.50 | 0.72 | 0.50 |
| **713** | 0.90 | 0.84 | 0.90 | 0.84 | 0.89 | 0.89 | 0.89 | 0.89 | 0.81 | 0.77 | 0.90 | 0.84 | 0.18 | 0.86 | 0.11 | 0.86 |
| **714** | 0.07 | 0.57 | 0.07 | 0.57 | 0.06 | 0.61 | 0.06 | 0.61 | 0.05 | 0.51 | 0.07 | 0.57 | 0.21 | 0.94 | 0.04 | 0.94 |
| **715** | 0.03 | 0.50 | 0.03 | 0.50 | 0.03 | 0.42 | 0.03 | 0.42 | 0.03 | 0.38 | 0.03 | 0.50 | 0.91 | 0.96 | 0.79 | 0.96 |
| **716** | 0.70 | 0.93 | 0.70 | 0.93 | 0.35 | 0.95 | 0.35 | 0.95 | 0.80 | 0.96 | 0.70 | 0.93 | 0.20 | 0.93 | 0.20 | 0.93 |
| **717** | 0.03 | 0.51 | 0.03 | 0.51 | 0.03 | 0.51 | 0.03 | 0.51 | 0.03 | 0.51 | 0.03 | 0.51 | 0.70 | 0.95 | 0.70 | 0.95 |
| **718** | 0.31 | 0.97 | 0.31 | 0.97 | 0.16 | 0.97 | 0.16 | 0.97 | 0.34 | 0.97 | 0.32 | 0.97 | 0.95 | 0.67 | 0.95 | 0.67 |
| **719** | 0.97 | 0.80 | 0.97 | 0.80 | 0.97 | 0.80 | 0.97 | 0.80 | 0.97 | 0.80 | 0.98 | 0.80 | 0.02 | 0.16 | 0.02 | 0.16 |
| **720** | 0.05 | 0.31 | 0.05 | 0.31 | 0.05 | 0.31 | 0.05 | 0.31 | 0.04 | 0.31 | 0.06 | 0.31 | 0.03 | 0.66 | 0.03 | 0.66 |
| **721** | 0.03 | 0.54 | 0.03 | 0.54 | 0.04 | 0.54 | 0.04 | 0.54 | 0.04 | 0.54 | 0.05 | 0.54 | 0.55 | 0.14 | 0.55 | 0.14 |
| **722** | 0.28 | 0.13 | 0.28 | 0.13 | 0.28 | 0.13 | 0.28 | 0.13 | 0.28 | 0.13 | 0.39 | 0.13 | 0.89 | 0.20 | 0.89 | 0.20 |
| **723** | 0.91 | 0.22 | 0.91 | 0.22 | 0.91 | 0.22 | 0.91 | 0.22 | 0.91 | 0.22 | 0.90 | 0.39 | 0.07 | 0.81 | 0.07 | 0.81 |
| **724** | 0.24 | 0.80 | 0.24 | 0.80 | 0.24 | 0.80 | 0.24 | 0.80 | 0.24 | 0.80 | 0.24 | 0.90 | 0.07 | 0.92 | 0.07 | 0.92 |
| **725** | 0.04 | 0.97 | 0.04 | 0.97 | 0.04 | 0.97 | 0.04 | 0.97 | 0.04 | 0.97 | 0.04 | 0.91 | 0.20 | 0.30 | 0.20 | 0.30 |
| **726** | 0.17 | 0.77 | 0.17 | 0.77 | 0.17 | 0.77 | 0.17 | 0.77 | 0.17 | 0.77 | 0.04 | 0.45 | 0.05 | 0.22 | 0.05 | 0.22 |
| **727** | 0.10 | 0.63 | 0.10 | 0.63 | 0.10 | 0.63 | 0.10 | 0.63 | 0.10 | 0.63 | 0.09 | 0.68 | 0.51 | 0.74 | 0.36 | 0.74 |
| **728** | 0.06 | 0.19 | 0.06 | 0.19 | 0.06 | 0.19 | 0.06 | 0.19 | 0.06 | 0.19 | 0.06 | 0.10 | 0.02 | 0.10 | 0.02 | 0.10 |
| **729** | 0.07 | 0.83 | 0.07 | 0.83 | 0.07 | 0.83 | 0.07 | 0.83 | 0.07 | 0.83 | 0.08 | 0.86 | 0.88 | 0.99 | 0.91 | 0.99 |
| **730** | 0.52 | 0.98 | 0.52 | 0.98 | 0.52 | 0.98 | 0.52 | 0.98 | 0.45 | 0.98 | 0.67 | 0.98 | 0.04 | 0.04 | 0.04 | 0.04 |
| **731** | 0.09 | 0.08 | 0.09 | 0.08 | 0.09 | 0.08 | 0.09 | 0.08 | 0.07 | 0.08 | 0.16 | 0.08 | 0.90 | 0.04 | 0.92 | 0.04 |
| **732** | 0.41 | 0.05 | 0.41 | 0.05 | 0.41 | 0.05 | 0.41 | 0.05 | 0.42 | 0.05 | 0.43 | 0.05 | 0.68 | 0.74 | 0.52 | 0.72 |
| **733** | 0.90 | 0.88 | 0.90 | 0.88 | 0.90 | 0.88 | 0.90 | 0.88 | 0.93 | 0.88 | 0.79 | 0.88 | 0.46 | 0.93 | 0.39 | 0.90 |
| **734** | 0.77 | 0.90 | 0.77 | 0.90 | 0.77 | 0.90 | 0.77 | 0.90 | 0.77 | 0.90 | 0.78 | 0.90 | 0.91 | 0.90 | 0.96 | 0.97 |
| **735** | 0.95 | 0.97 | 0.95 | 0.97 | 0.95 | 0.97 | 0.95 | 0.97 | 0.97 | 0.96 | 0.95 | 0.96 | 0.52 | 0.80 | 0.66 | 0.46 |
| **736** | 0.72 | 0.82 | 0.72 | 0.82 | 0.72 | 0.82 | 0.72 | 0.82 | 0.67 | 0.85 | 0.80 | 0.63 | 0.88 | 0.36 | 0.88 | 0.32 |
| **737** | 0.93 | 0.23 | 0.93 | 0.23 | 0.93 | 0.23 | 0.93 | 0.23 | 0.97 | 0.26 | 0.97 | 0.41 | 0.05 | 0.24 | 0.05 | 0.18 |
| **738** | 0.05 | 0.37 | 0.05 | 0.37 | 0.05 | 0.37 | 0.05 | 0.37 | 0.04 | 0.87 | 0.03 | 0.81 | 0.62 | 0.53 | 0.82 | 0.49 |
| **739** | 0.09 | 0.32 | 0.09 | 0.32 | 0.09 | 0.32 | 0.09 | 0.32 | 0.30 | 0.17 | 0.36 | 0.43 | 0.16 | 0.07 | 0.65 | 0.15 |
| **740** | 0.02 | 0.04 | 0.02 | 0.04 | 0.02 | 0.04 | 0.02 | 0.04 | 0.02 | 0.07 | 0.03 | 0.04 | 0.47 | 0.04 | 0.69 | 0.91 |
| **741** | 0.07 | 0.07 | 0.07 | 0.07 | 0.07 | 0.07 | 0.07 | 0.07 | 0.11 | 0.04 | 0.13 | 0.04 | 0.18 | 0.03 | 0.04 | 0.04 |
| **742** | 0.04 | 0.03 | 0.04 | 0.03 | 0.04 | 0.03 | 0.04 | 0.03 | 0.03 | 0.05 | 0.02 | 0.04 | 0.94 | 0.99 | 0.60 | 0.96 |
| **743** | 0.76 | 0.97 | 0.76 | 0.97 | 0.76 | 0.97 | 0.76 | 0.97 | 0.86 | 0.96 | 0.93 | 0.98 | 0.97 | 0.07 | 0.96 | 0.18 |
| **744** | 0.95 | 0.21 | 0.95 | 0.21 | 0.95 | 0.21 | 0.95 | 0.21 | 0.86 | 0.13 | 0.91 | 0.12 | 0.15 | 0.61 | 0.09 | 0.64 |
| **745** | 0.32 | 0.89 | 0.32 | 0.89 | 0.32 | 0.89 | 0.32 | 0.89 | 0.17 | 0.65 | 0.24 | 0.71 | 0.89 | 0.58 | 0.86 | 0.73 |
| **746** | 0.86 | 0.53 | 0.86 | 0.53 | 0.86 | 0.53 | 0.86 | 0.53 | 0.61 | 0.36 | 0.38 | 0.36 | 0.08 | 0.57 | 0.03 | 0.57 |
| **747** | 0.04 | 0.62 | 0.04 | 0.62 | 0.04 | 0.62 | 0.04 | 0.62 | 0.05 | 0.62 | 0.04 | 0.62 | 0.03 | 0.18 | 0.03 | 0.19 |
| **748** | 0.03 | 0.18 | 0.03 | 0.18 | 0.03 | 0.18 | 0.03 | 0.18 | 0.02 | 0.37 | 0.03 | 0.18 | 0.07 | 0.16 | 0.10 | 0.43 |
| **749** | 0.15 | 0.64 | 0.15 | 0.64 | 0.15 | 0.64 | 0.15 | 0.64 | 0.17 | 0.61 | 0.31 | 0.64 | 0.03 | 0.25 | 0.03 | 0.43 |
| **750** | 0.03 | 0.69 | 0.03 | 0.69 | 0.03 | 0.69 | 0.03 | 0.69 | 0.04 | 0.44 | 0.04 | 0.69 | 0.03 | 0.24 | 0.03 | 0.73 |
| **751** | 0.02 | 0.65 | 0.02 | 0.65 | 0.02 | 0.65 | 0.02 | 0.65 | 0.29 | 0.96 | 0.02 | 0.48 | 0.03 | 0.05 | 0.03 | 0.75 |
| **752** | 0.03 | 0.84 | 0.03 | 0.84 | 0.03 | 0.84 | 0.03 | 0.84 | 0.02 | 0.72 | 0.03 | 0.90 | 0.84 | 0.64 | 0.90 | 0.85 |
| **753** | 0.96 | 0.88 | 0.96 | 0.88 | 0.96 | 0.88 | 0.96 | 0.88 | 0.95 | 0.93 | 0.95 | 0.68 | 0.26 | 0.95 | 0.32 | 0.90 |
| **754** | 0.84 | 0.92 | 0.84 | 0.92 | 0.84 | 0.92 | 0.84 | 0.92 | 0.70 | 0.88 | 0.30 | 0.91 | 0.03 | 0.52 | 0.03 | 0.52 |
| **755** | 0.05 | 0.51 | 0.05 | 0.51 | 0.05 | 0.51 | 0.05 | 0.51 | 0.03 | 0.22 | 0.03 | 0.35 | 0.05 | 0.25 | 0.04 | 0.25 |
| **756** | 0.03 | 0.13 | 0.03 | 0.13 | 0.03 | 0.13 | 0.03 | 0.13 | 0.03 | 0.05 | 0.03 | 0.18 | 0.92 | 0.93 | 0.79 | 0.95 |
| **757** | 0.96 | 0.98 | 0.96 | 0.98 | 0.96 | 0.98 | 0.96 | 0.98 | 0.93 | 0.98 | 0.94 | 0.96 | 0.68 | 0.15 | 0.34 | 0.46 |
| **758** | 0.19 | 0.77 | 0.19 | 0.77 | 0.19 | 0.77 | 0.19 | 0.77 | 0.59 | 0.14 | 0.69 | 0.26 | 0.59 | 0.31 | 0.79 | 0.77 |
| **759** | 0.93 | 0.84 | 0.93 | 0.84 | 0.92 | 0.84 | 0.92 | 0.84 | 0.92 | 0.85 | 0.96 | 0.34 | 0.64 | 0.33 | 0.34 | 0.75 |
| **760** | 0.34 | 0.73 | 0.34 | 0.73 | 0.35 | 0.73 | 0.35 | 0.73 | 0.63 | 0.52 | 0.94 | 0.38 | 0.88 | 0.81 | 0.32 | 0.96 |
| **761** | 0.69 | 0.95 | 0.69 | 0.95 | 0.68 | 0.95 | 0.68 | 0.95 | 0.82 | 0.95 | 0.85 | 0.70 | 0.03 | 0.97 | 0.03 | 0.98 |
| **762** | 0.03 | 0.96 | 0.03 | 0.96 | 0.03 | 0.96 | 0.03 | 0.96 | 0.03 | 0.94 | 0.04 | 0.91 | 0.06 | 0.47 | 0.04 | 0.28 |
| **763** | 0.06 | 0.07 | 0.06 | 0.07 | 0.06 | 0.07 | 0.06 | 0.07 | 0.12 | 0.13 | 0.07 | 0.22 | 0.03 | 0.06 | 0.02 | 0.11 |
| **764** | 0.02 | 0.69 | 0.02 | 0.69 | 0.03 | 0.69 | 0.03 | 0.69 | 0.03 | 0.70 | 0.03 | 0.33 | 0.91 | 0.21 | 0.93 | 0.19 |
| **765** | 0.08 | 0.03 | 0.08 | 0.03 | 0.05 | 0.04 | 0.05 | 0.04 | 0.03 | 0.04 | 0.06 | 0.03 | 0.03 | 0.47 | 0.03 | 0.47 |
| **766** | 0.12 | 0.04 | 0.12 | 0.04 | 0.08 | 0.03 | 0.08 | 0.03 | 0.07 | 0.04 | 0.03 | 0.03 | 0.04 | 0.88 | 0.03 | 0.88 |
| **767** | 0.02 | 0.25 | 0.02 | 0.25 | 0.02 | 0.32 | 0.02 | 0.32 | 0.02 | 0.25 | 0.02 | 0.37 | 0.03 | 0.40 | 0.10 | 0.40 |
| **768** | 0.95 | 0.90 | 0.95 | 0.90 | 0.92 | 0.77 | 0.92 | 0.77 | 0.94 | 0.90 | 0.78 | 0.81 | 0.03 | 0.07 | 0.02 | 0.07 |
| **769** | 0.04 | 0.46 | 0.04 | 0.46 | 0.03 | 0.41 | 0.03 | 0.41 | 0.03 | 0.46 | 0.06 | 0.85 | 0.12 | 0.83 | 0.12 | 0.83 |
| **770** | 0.96 | 0.94 | 0.96 | 0.94 | 0.95 | 0.92 | 0.95 | 0.92 | 0.90 | 0.95 | 0.94 | 0.86 | 0.02 | 0.67 | 0.02 | 0.67 |
| **771** | 0.05 | 0.32 | 0.05 | 0.32 | 0.07 | 0.32 | 0.07 | 0.32 | 0.05 | 0.51 | 0.02 | 0.09 | 0.76 | 0.97 | 0.77 | 0.97 |
| **772** | 0.96 | 0.87 | 0.96 | 0.87 | 0.96 | 0.87 | 0.96 | 0.87 | 0.91 | 0.62 | 0.96 | 0.91 | 0.90 | 0.46 | 0.79 | 0.46 |
| **773** | 0.02 | 0.25 | 0.02 | 0.25 | 0.02 | 0.25 | 0.02 | 0.25 | 0.06 | 0.71 | 0.03 | 0.38 | 0.52 | 0.93 | 0.44 | 0.93 |
| **774** | 0.94 | 0.49 | 0.94 | 0.49 | 0.90 | 0.49 | 0.90 | 0.49 | 0.88 | 0.53 | 0.94 | 0.23 | 0.02 | 0.22 | 0.02 | 0.54 |
| **775** | 0.06 | 0.89 | 0.06 | 0.89 | 0.07 | 0.89 | 0.07 | 0.89 | 0.07 | 0.96 | 0.44 | 0.58 | 0.34 | 0.15 | 0.21 | 0.70 |
| **776** | 0.04 | 0.64 | 0.04 | 0.64 | 0.04 | 0.64 | 0.04 | 0.64 | 0.03 | 0.57 | 0.03 | 0.44 | 0.05 | 0.71 | 0.04 | 0.83 |
| **777** | 0.04 | 0.83 | 0.04 | 0.83 | 0.04 | 0.83 | 0.04 | 0.83 | 0.06 | 0.95 | 0.04 | 0.91 | 0.80 | 0.40 | 0.69 | 0.91 |
| **778** | 0.89 | 0.21 | 0.89 | 0.21 | 0.89 | 0.21 | 0.89 | 0.21 | 0.71 | 0.13 | 0.03 | 0.09 | 0.03 | 0.47 | 0.03 | 0.59 |
| **779** | 0.14 | 0.35 | 0.14 | 0.35 | 0.14 | 0.35 | 0.14 | 0.35 | 0.12 | 0.53 | 0.17 | 0.06 | 0.81 | 0.45 | 0.92 | 0.27 |
| **780** | 0.04 | 0.14 | 0.04 | 0.14 | 0.03 | 0.14 | 0.03 | 0.14 | 0.03 | 0.14 | 0.03 | 0.16 | 0.31 | 0.44 | 0.38 | 0.21 |
| **781** | 0.22 | 0.66 | 0.22 | 0.66 | 0.16 | 0.66 | 0.16 | 0.66 | 0.12 | 0.87 | 0.95 | 0.29 | 0.05 | 0.14 | 0.03 | 0.14 |
| **782** | 0.77 | 0.73 | 0.77 | 0.73 | 0.84 | 0.73 | 0.84 | 0.73 | 0.64 | 0.81 | 0.07 | 0.08 | 0.11 | 0.17 | 0.87 | 0.29 |
| **783** | 0.37 | 0.51 | 0.37 | 0.51 | 0.33 | 0.51 | 0.33 | 0.51 | 0.05 | 0.87 | 0.92 | 0.69 | 0.79 | 0.21 | 0.49 | 0.31 |
| **784** | 0.24 | 0.94 | 0.24 | 0.94 | 0.20 | 0.94 | 0.20 | 0.94 | 0.96 | 0.86 | 0.19 | 0.78 | 0.30 | 0.59 | 0.07 | 0.91 |
| **785** | 0.93 | 0.95 | 0.93 | 0.95 | 0.94 | 0.94 | 0.94 | 0.94 | 0.86 | 0.52 | 0.98 | 0.94 | 0.06 | 0.47 | 0.11 | 0.72 |
| **786** | 0.03 | 0.32 | 0.03 | 0.32 | 0.03 | 0.41 | 0.03 | 0.41 | 0.03 | 0.10 | 0.04 | 0.09 | 0.76 | 0.56 | 0.87 | 0.30 |
| **787** | 0.24 | 0.55 | 0.24 | 0.55 | 0.25 | 0.85 | 0.25 | 0.85 | 0.80 | 0.62 | 0.79 | 0.66 | 0.09 | 0.04 | 0.22 | 0.11 |
| **788** | 0.22 | 0.68 | 0.22 | 0.68 | 0.03 | 0.45 | 0.03 | 0.45 | 0.22 | 0.08 | 0.17 | 0.36 | 0.85 | 0.44 | 0.92 | 0.78 |
| **789** | 0.03 | 0.06 | 0.03 | 0.06 | 0.38 | 0.49 | 0.38 | 0.49 | 0.03 | 0.06 | 0.04 | 0.09 | 0.20 | 0.14 | 0.36 | 0.09 |
| **790** | 0.03 | 0.03 | 0.03 | 0.03 | 0.03 | 0.06 | 0.03 | 0.06 | 0.02 | 0.07 | 0.02 | 0.03 | 0.03 | 0.11 | 0.95 | 0.62 |
| **791** | 0.03 | 0.35 | 0.03 | 0.35 | 0.03 | 0.62 | 0.03 | 0.62 | 0.03 | 0.15 | 0.02 | 0.52 | 0.02 | 0.04 | 0.02 | 0.09 |
| **792** | 0.50 | 0.34 | 0.50 | 0.34 | 0.21 | 0.12 | 0.21 | 0.12 | 0.02 | 0.37 | 0.05 | 0.37 | 0.03 | 0.22 | 0.03 | 0.79 |
| **793** | 0.03 | 0.14 | 0.03 | 0.14 | 0.03 | 0.17 | 0.03 | 0.17 | 0.03 | 0.04 | 0.03 | 0.05 | 0.04 | 0.66 | 0.03 | 0.23 |
| **794** | 0.97 | 0.52 | 0.97 | 0.52 | 0.97 | 0.66 | 0.97 | 0.66 | 0.84 | 0.84 | 0.78 | 0.74 | 0.03 | 0.06 | 0.02 | 0.10 |
| **795** | 0.03 | 0.04 | 0.03 | 0.04 | 0.10 | 0.27 | 0.10 | 0.27 | 0.19 | 0.32 | 0.06 | 0.07 | 0.61 | 0.31 | 0.67 | 0.43 |
| **796** | 0.66 | 0.05 | 0.66 | 0.05 | 0.28 | 0.54 | 0.28 | 0.54 | 0.97 | 0.80 | 0.96 | 0.12 | 0.93 | 0.71 | 0.95 | 0.70 |
| **797** | 0.03 | 0.10 | 0.03 | 0.10 | 0.05 | 0.11 | 0.05 | 0.11 | 0.05 | 0.07 | 0.04 | 0.36 | 0.97 | 0.57 | 0.90 | 0.57 |
| **798** | 0.07 | 0.93 | 0.07 | 0.93 | 0.08 | 0.82 | 0.08 | 0.82 | 0.97 | 0.96 | 0.09 | 0.62 | 0.21 | 0.44 | 0.20 | 0.44 |
| **799** | 0.06 | 0.26 | 0.06 | 0.26 | 0.03 | 0.41 | 0.03 | 0.41 | 0.03 | 0.62 | 0.03 | 0.44 | 0.04 | 0.85 | 0.09 | 0.85 |
| **800** | 0.16 | 0.21 | 0.16 | 0.21 | 0.06 | 0.39 | 0.06 | 0.39 | 0.03 | 0.69 | 0.03 | 0.28 | 0.02 | 0.21 | 0.04 | 0.18 |
| **801** | 0.76 | 0.39 | 0.76 | 0.39 | 0.42 | 0.17 | 0.42 | 0.17 | 0.09 | 0.84 | 0.18 | 0.21 | 0.04 | 0.13 | 0.03 | 0.28 |
| **802** | 0.47 | 0.68 | 0.47 | 0.68 | 0.03 | 0.22 | 0.03 | 0.22 | 0.03 | 0.62 | 0.14 | 0.37 | 0.13 | 0.14 | 0.10 | 0.08 |
| **803** | 0.05 | 0.81 | 0.05 | 0.81 | 0.07 | 0.83 | 0.07 | 0.83 | 0.02 | 0.41 | 0.03 | 0.72 | 0.02 | 0.58 | 0.03 | 0.69 |
| **804** | 0.42 | 0.88 | 0.42 | 0.88 | 0.25 | 0.70 | 0.25 | 0.70 | 0.25 | 0.93 | 0.76 | 0.93 | 0.03 | 0.87 | 0.02 | 0.66 |
| **805** | 0.10 | 0.80 | 0.10 | 0.80 | 0.11 | 0.81 | 0.11 | 0.81 | 0.15 | 0.60 | 0.24 | 0.56 | 0.33 | 0.50 | 0.40 | 0.79 |
| **806** | 0.02 | 0.89 | 0.02 | 0.89 | 0.03 | 0.89 | 0.03 | 0.89 | 0.02 | 0.23 | 0.02 | 0.19 | 0.10 | 0.66 | 0.10 | 0.37 |
| **807** | 0.36 | 0.74 | 0.36 | 0.74 | 0.62 | 0.74 | 0.62 | 0.74 | 0.41 | 0.94 | 0.21 | 0.93 | 0.09 | 0.90 | 0.03 | 0.15 |
| **808** | 0.08 | 0.12 | 0.08 | 0.12 | 0.09 | 0.12 | 0.09 | 0.12 | 0.75 | 0.10 | 0.42 | 0.13 | 0.39 | 0.71 | 0.71 | 0.96 |
| **809** | 0.29 | 0.93 | 0.29 | 0.93 | 0.16 | 0.93 | 0.16 | 0.93 | 0.26 | 0.95 | 0.42 | 0.95 | 0.62 | 0.63 | 0.71 | 0.18 |
| **810** | 0.05 | 0.75 | 0.05 | 0.75 | 0.06 | 0.75 | 0.06 | 0.75 | 0.03 | 0.75 | 0.03 | 0.75 | 0.32 | 0.96 | 0.03 | 0.90 |
| **811** | 0.97 | 0.98 | 0.97 | 0.98 | 0.97 | 0.98 | 0.97 | 0.98 | 0.96 | 0.99 | 0.95 | 0.99 | 0.03 | 0.85 | 0.10 | 0.82 |
| **812** | 0.65 | 0.43 | 0.65 | 0.43 | 0.65 | 0.43 | 0.65 | 0.43 | 0.42 | 0.53 | 0.28 | 0.53 | 0.96 | 0.92 | 0.96 | 0.98 |
| **813** | 0.79 | 0.95 | 0.79 | 0.95 | 0.79 | 0.95 | 0.79 | 0.95 | 0.33 | 0.79 | 0.48 | 0.79 | 0.04 | 0.26 | 0.06 | 0.31 |
| **814** | 0.22 | 0.90 | 0.22 | 0.90 | 0.22 | 0.90 | 0.22 | 0.90 | 0.34 | 0.92 | 0.23 | 0.92 | 0.95 | 0.93 | 0.50 | 0.93 |
| **815** | 0.04 | 0.56 | 0.04 | 0.56 | 0.04 | 0.56 | 0.04 | 0.56 | 0.05 | 0.55 | 0.03 | 0.60 | 0.03 | 0.76 | 0.03 | 0.84 |
| **816** | 0.40 | 0.57 | 0.40 | 0.57 | 0.40 | 0.57 | 0.40 | 0.57 | 0.44 | 0.81 | 0.49 | 0.80 | 0.61 | 0.84 | 0.79 | 0.79 |
| **817** | 0.88 | 0.39 | 0.88 | 0.39 | 0.88 | 0.39 | 0.88 | 0.39 | 0.95 | 0.39 | 0.96 | 0.27 | 0.04 | 0.05 | 0.05 | 0.09 |
| **818** | 0.03 | 0.94 | 0.03 | 0.94 | 0.03 | 0.94 | 0.03 | 0.94 | 0.03 | 0.83 | 0.02 | 0.92 | 0.78 | 0.43 | 0.90 | 0.40 |
| **819** | 0.04 | 0.19 | 0.04 | 0.19 | 0.04 | 0.19 | 0.04 | 0.19 | 0.04 | 0.14 | 0.64 | 0.85 | 0.02 | 0.40 | 0.03 | 0.37 |
| **820** | 0.03 | 0.12 | 0.03 | 0.12 | 0.03 | 0.12 | 0.03 | 0.12 | 0.04 | 0.10 | 0.03 | 0.32 | 0.05 | 0.77 | 0.06 | 0.81 |
| **821** | 0.77 | 0.81 | 0.77 | 0.81 | 0.77 | 0.81 | 0.77 | 0.81 | 0.74 | 0.69 | 0.90 | 0.86 | 0.14 | 0.44 | 0.12 | 0.10 |
| **822** | 0.03 | 0.96 | 0.03 | 0.96 | 0.03 | 0.96 | 0.03 | 0.96 | 0.03 | 0.95 | 0.06 | 0.98 | 0.05 | 0.14 | 0.10 | 0.26 |
| **823** | 0.76 | 0.71 | 0.76 | 0.71 | 0.76 | 0.71 | 0.76 | 0.71 | 0.23 | 0.32 | 0.93 | 0.63 | 0.14 | 0.94 | 0.81 | 0.71 |
| **824** | 0.90 | 0.54 | 0.90 | 0.54 | 0.90 | 0.54 | 0.90 | 0.54 | 0.91 | 0.27 | 0.08 | 0.92 | 0.41 | 0.82 | 0.52 | 0.44 |
| **825** | 0.95 | 0.87 | 0.95 | 0.87 | 0.95 | 0.87 | 0.95 | 0.87 | 0.68 | 0.85 | 0.50 | 0.86 | 0.84 | 0.48 | 0.62 | 0.92 |
| **826** | 0.04 | 0.64 | 0.04 | 0.64 | 0.04 | 0.64 | 0.04 | 0.64 | 0.89 | 0.32 | 0.02 | 0.80 | 0.61 | 0.86 | 0.40 | 0.77 |
| **827** | 0.05 | 0.55 | 0.05 | 0.55 | 0.05 | 0.55 | 0.05 | 0.55 | 0.03 | 0.39 | 0.06 | 0.04 | 0.02 | 0.90 | 0.03 | 0.90 |
| **828** | 0.55 | 0.75 | 0.55 | 0.75 | 0.55 | 0.75 | 0.55 | 0.75 | 0.13 | 0.75 | 0.08 | 0.92 | 0.05 | 0.48 | 0.03 | 0.35 |
| **829** | 0.03 | 0.07 | 0.03 | 0.07 | 0.03 | 0.07 | 0.03 | 0.07 | 0.02 | 0.45 | 0.36 | 0.06 | 0.34 | 0.80 | 0.09 | 0.60 |
| **830** | 0.02 | 0.73 | 0.02 | 0.73 | 0.02 | 0.73 | 0.02 | 0.73 | 0.03 | 0.37 | 0.04 | 0.79 | 0.03 | 0.12 | 0.02 | 0.06 |
| **831** | 0.88 | 0.56 | 0.88 | 0.56 | 0.84 | 0.56 | 0.84 | 0.56 | 0.22 | 0.35 | 0.83 | 0.64 | 0.02 | 0.78 | 0.02 | 0.70 |
| **832** | 0.29 | 0.35 | 0.29 | 0.35 | 0.31 | 0.35 | 0.31 | 0.35 | 0.72 | 0.55 | 0.24 | 0.39 | 0.95 | 0.22 | 0.95 | 0.47 |
| **833** | 0.03 | 0.08 | 0.03 | 0.08 | 0.03 | 0.08 | 0.03 | 0.08 | 0.02 | 0.44 | 0.02 | 0.04 | 0.24 | 0.53 | 0.65 | 0.53 |
| **834** | 0.68 | 0.51 | 0.68 | 0.51 | 0.72 | 0.51 | 0.72 | 0.51 | 0.93 | 0.96 | 0.73 | 0.25 | 0.03 | 0.09 | 0.03 | 0.04 |
| **835** | 0.42 | 0.35 | 0.42 | 0.35 | 0.68 | 0.35 | 0.68 | 0.35 | 0.06 | 0.25 | 0.07 | 0.23 | 0.09 | 0.23 | 0.82 | 0.66 |
| **836** | 0.77 | 0.88 | 0.77 | 0.88 | 0.77 | 0.94 | 0.77 | 0.94 | 0.03 | 0.40 | 0.45 | 0.69 | 0.07 | 0.60 | 0.08 | 0.31 |
| **837** | 0.96 | 0.90 | 0.96 | 0.90 | 0.97 | 0.93 | 0.97 | 0.93 | 0.65 | 0.84 | 0.94 | 0.91 | 0.51 | 0.97 | 0.54 | 0.98 |
| **838** | 0.72 | 0.95 | 0.72 | 0.95 | 0.83 | 0.95 | 0.83 | 0.95 | 0.19 | 0.81 | 0.96 | 0.38 | 0.87 | 0.93 | 0.83 | 0.78 |
| **839** | 0.95 | 0.72 | 0.95 | 0.72 | 0.97 | 0.86 | 0.97 | 0.86 | 0.86 | 0.72 | 0.54 | 0.74 | 0.21 | 0.91 | 0.19 | 0.94 |
| **840** | 0.77 | 0.79 | 0.77 | 0.79 | 0.68 | 0.69 | 0.68 | 0.69 | 0.55 | 0.79 | 0.71 | 0.72 | 0.98 | 0.94 | 0.97 | 0.96 |
| **841** | 0.03 | 0.97 | 0.03 | 0.97 | 0.03 | 0.96 | 0.03 | 0.96 | 0.04 | 0.94 | 0.02 | 0.92 | 0.96 | 0.38 | 0.95 | 0.32 |
| **842** | 0.03 | 0.54 | 0.03 | 0.54 | 0.03 | 0.50 | 0.03 | 0.50 | 0.03 | 0.74 | 0.03 | 0.74 | 0.02 | 0.37 | 0.03 | 0.21 |
| **843** | 0.59 | 0.51 | 0.59 | 0.51 | 0.29 | 0.51 | 0.29 | 0.51 | 0.04 | 0.29 | 0.09 | 0.29 | 0.23 | 0.60 | 0.03 | 0.66 |
| **844** | 0.03 | 0.14 | 0.03 | 0.14 | 0.03 | 0.14 | 0.03 | 0.14 | 0.02 | 0.35 | 0.03 | 0.35 | 0.66 | 0.17 | 0.46 | 0.25 |
| **845** | 0.97 | 0.83 | 0.97 | 0.83 | 0.97 | 0.83 | 0.97 | 0.83 | 0.97 | 0.75 | 0.89 | 0.75 | 0.03 | 0.12 | 0.02 | 0.12 |
| **846** | 0.04 | 0.29 | 0.04 | 0.29 | 0.05 | 0.29 | 0.05 | 0.29 | 0.03 | 0.56 | 0.03 | 0.44 | 0.89 | 0.92 | 0.76 | 0.80 |
| **847** | 0.96 | 0.76 | 0.96 | 0.76 | 0.96 | 0.76 | 0.96 | 0.76 | 0.97 | 0.80 | 0.96 | 0.76 | 0.03 | 0.56 | 0.02 | 0.47 |
| **848** | 0.20 | 0.87 | 0.20 | 0.87 | 0.20 | 0.87 | 0.20 | 0.87 | 0.33 | 0.63 | 0.29 | 0.87 | 0.85 | 0.64 | 0.94 | 0.87 |
| **849** | 0.75 | 0.86 | 0.75 | 0.86 | 0.75 | 0.86 | 0.75 | 0.86 | 0.88 | 0.90 | 0.45 | 0.86 | 0.44 | 0.73 | 0.21 | 0.67 |
| **850** | 0.03 | 0.79 | 0.03 | 0.79 | 0.03 | 0.79 | 0.03 | 0.79 | 0.02 | 0.64 | 0.02 | 0.79 | 0.95 | 0.91 | 0.94 | 0.95 |
| **851** | 0.03 | 0.29 | 0.03 | 0.29 | 0.03 | 0.29 | 0.03 | 0.29 | 0.03 | 0.36 | 0.03 | 0.35 | 0.03 | 0.44 | 0.02 | 0.43 |
| **852** | 0.06 | 0.50 | 0.06 | 0.50 | 0.06 | 0.50 | 0.06 | 0.50 | 0.08 | 0.24 | 0.06 | 0.71 | 0.06 | 0.60 | 0.05 | 0.73 |
| **853** | 0.03 | 0.10 | 0.03 | 0.10 | 0.03 | 0.10 | 0.03 | 0.10 | 0.04 | 0.11 | 0.03 | 0.13 | 0.21 | 0.62 | 0.31 | 0.49 |
| **854** | 0.02 | 0.72 | 0.02 | 0.72 | 0.02 | 0.72 | 0.02 | 0.72 | 0.02 | 0.78 | 0.02 | 0.29 | 0.08 | 0.34 | 0.03 | 0.26 |
| **855** | 0.71 | 0.56 | 0.71 | 0.56 | 0.71 | 0.56 | 0.71 | 0.56 | 0.86 | 0.24 | 0.89 | 0.20 | 0.02 | 0.95 | 0.02 | 0.95 |
| **856** | 0.03 | 0.40 | 0.03 | 0.40 | 0.03 | 0.40 | 0.03 | 0.40 | 0.02 | 0.80 | 0.04 | 0.12 | 0.81 | 0.53 | 0.90 | 0.49 |
| **857** | 0.42 | 0.35 | 0.42 | 0.35 | 0.42 | 0.35 | 0.42 | 0.35 | 0.20 | 0.17 | 0.63 | 0.35 | 0.02 | 0.74 | 0.03 | 0.32 |
| **858** | 0.07 | 0.95 | 0.07 | 0.95 | 0.07 | 0.95 | 0.07 | 0.95 | 0.02 | 0.59 | 0.19 | 0.49 | 0.25 | 0.35 | 0.23 | 0.54 |
| **859** | 0.03 | 0.67 | 0.03 | 0.67 | 0.03 | 0.67 | 0.03 | 0.67 | 0.03 | 0.51 | 0.04 | 0.53 | 0.02 | 0.30 | 0.03 | 0.59 |
| **860** | 0.25 | 0.87 | 0.25 | 0.87 | 0.25 | 0.87 | 0.25 | 0.87 | 0.42 | 0.23 | 0.60 | 0.88 | 0.03 | 0.74 | 0.03 | 0.59 |
| **861** | 0.05 | 0.93 | 0.05 | 0.93 | 0.05 | 0.93 | 0.05 | 0.93 | 0.08 | 0.83 | 0.07 | 0.78 | 0.55 | 0.37 | 0.45 | 0.29 |
| **862** | 0.39 | 0.92 | 0.39 | 0.92 | 0.39 | 0.92 | 0.39 | 0.92 | 0.75 | 0.34 | 0.03 | 0.78 | 0.02 | 0.63 | 0.02 | 0.66 |
| **863** | 0.03 | 0.96 | 0.03 | 0.96 | 0.03 | 0.96 | 0.03 | 0.96 | 0.05 | 0.94 | 0.05 | 0.97 | 0.30 | 0.93 | 0.56 | 0.96 |
| **864** | 0.05 | 0.14 | 0.05 | 0.14 | 0.05 | 0.14 | 0.05 | 0.14 | 0.07 | 0.19 | 0.16 | 0.15 | 0.08 | 0.96 | 0.02 | 0.75 |
| **865** | 0.96 | 0.42 | 0.96 | 0.42 | 0.96 | 0.42 | 0.96 | 0.42 | 0.76 | 0.27 | 0.03 | 0.05 | 0.29 | 0.58 | 0.05 | 0.48 |
| **866** | 0.97 | 0.86 | 0.97 | 0.86 | 0.97 | 0.86 | 0.97 | 0.86 | 0.97 | 0.86 | 0.97 | 0.87 | 0.33 | 0.60 | 0.78 | 0.70 |
| **867** | 0.62 | 0.71 | 0.62 | 0.71 | 0.62 | 0.71 | 0.62 | 0.71 | 0.73 | 0.57 | 0.19 | 0.39 | 0.96 | 0.98 | 0.97 | 0.99 |
| **868** | 0.11 | 0.42 | 0.11 | 0.42 | 0.11 | 0.42 | 0.11 | 0.42 | 0.21 | 0.60 | 0.13 | 0.21 | 0.11 | 0.95 | 0.53 | 0.97 |
| **869** | 0.04 | 0.77 | 0.04 | 0.77 | 0.04 | 0.77 | 0.04 | 0.77 | 0.03 | 0.53 | 0.03 | 0.94 | 0.09 | 0.93 | 0.10 | 0.89 |
| **870** | 0.72 | 0.28 | 0.72 | 0.28 | 0.72 | 0.28 | 0.72 | 0.28 | 0.47 | 0.22 | 0.84 | 0.20 | 0.97 | 0.85 | 0.97 | 0.87 |
| **871** | 0.03 | 0.85 | 0.03 | 0.85 | 0.03 | 0.85 | 0.03 | 0.85 | 0.03 | 0.71 | 0.02 | 0.67 | 0.05 | 0.65 | 0.03 | 0.78 |
| **872** | 0.33 | 0.95 | 0.33 | 0.95 | 0.33 | 0.95 | 0.33 | 0.95 | 0.11 | 0.96 | 0.05 | 0.96 | 0.46 | 0.63 | 0.58 | 0.53 |
| **873** | 0.03 | 0.19 | 0.03 | 0.19 | 0.03 | 0.19 | 0.03 | 0.19 | 0.06 | 0.30 | 0.02 | 0.15 | 0.03 | 0.07 | 0.03 | 0.06 |
| **874** | 0.07 | 0.21 | 0.07 | 0.21 | 0.07 | 0.21 | 0.07 | 0.21 | 0.08 | 0.25 | 0.09 | 0.65 | 0.03 | 0.63 | 0.02 | 0.64 |
| **875** | 0.07 | 0.17 | 0.07 | 0.17 | 0.07 | 0.17 | 0.07 | 0.17 | 0.15 | 0.32 | 0.13 | 0.25 | 0.61 | 0.38 | 0.38 | 0.26 |
| **876** | 0.29 | 0.17 | 0.29 | 0.17 | 0.29 | 0.17 | 0.29 | 0.17 | 0.18 | 0.78 | 0.46 | 0.15 | 0.07 | 0.13 | 0.06 | 0.09 |
| **877** | 0.86 | 0.93 | 0.86 | 0.93 | 0.86 | 0.93 | 0.86 | 0.93 | 0.49 | 0.77 | 0.69 | 0.92 | 0.38 | 0.98 | 0.20 | 0.96 |
| **878** | 0.05 | 0.79 | 0.05 | 0.79 | 0.05 | 0.79 | 0.05 | 0.79 | 0.03 | 0.89 | 0.03 | 0.84 | 0.11 | 0.65 | 0.10 | 0.44 |
| **879** | 0.81 | 0.98 | 0.81 | 0.98 | 0.81 | 0.98 | 0.81 | 0.98 | 0.79 | 0.97 | 0.18 | 0.98 | 0.68 | 0.93 | 0.47 | 0.91 |
| **880** | 0.17 | 0.62 | 0.17 | 0.62 | 0.20 | 0.62 | 0.20 | 0.62 | 0.74 | 0.86 | 0.11 | 0.42 | 0.20 | 0.86 | 0.14 | 0.84 |
| **881** | 0.62 | 0.98 | 0.62 | 0.98 | 0.68 | 0.98 | 0.68 | 0.98 | 0.49 | 0.95 | 0.48 | 0.97 | 0.70 | 0.98 | 0.94 | 0.98 |
| **882** | 0.04 | 0.91 | 0.04 | 0.91 | 0.06 | 0.91 | 0.06 | 0.91 | 0.05 | 0.85 | 0.04 | 0.90 | 0.02 | 0.34 | 0.03 | 0.34 |
| **883** | 0.97 | 0.96 | 0.97 | 0.96 | 0.96 | 0.96 | 0.96 | 0.96 | 0.95 | 0.91 | 0.96 | 0.91 | 0.49 | 0.69 | 0.89 | 0.69 |
| **884** | 0.29 | 0.62 | 0.29 | 0.62 | 0.47 | 0.62 | 0.47 | 0.62 | 0.18 | 0.74 | 0.44 | 0.74 | 0.30 | 0.81 | 0.78 | 0.67 |
| **885** | 0.95 | 0.98 | 0.95 | 0.98 | 0.96 | 0.98 | 0.96 | 0.98 | 0.97 | 0.99 | 0.97 | 0.99 | 0.97 | 0.30 | 0.96 | 0.32 |
| **886** | 0.03 | 0.52 | 0.03 | 0.52 | 0.03 | 0.17 | 0.03 | 0.17 | 0.19 | 0.19 | 0.04 | 0.51 | 0.04 | 0.80 | 0.04 | 0.93 |
| **887** | 0.90 | 0.97 | 0.90 | 0.97 | 0.92 | 0.98 | 0.92 | 0.98 | 0.98 | 0.89 | 0.96 | 0.83 | 0.73 | 0.79 | 0.05 | 0.46 |
| **888** | 0.02 | 0.10 | 0.02 | 0.10 | 0.02 | 0.13 | 0.02 | 0.13 | 0.02 | 0.14 | 0.02 | 0.08 | 0.02 | 0.27 | 0.03 | 0.58 |
| **889** | 0.04 | 0.51 | 0.04 | 0.51 | 0.07 | 0.41 | 0.07 | 0.41 | 0.11 | 0.46 | 0.04 | 0.16 | 0.03 | 0.04 | 0.03 | 0.09 |
| **890** | 0.04 | 0.74 | 0.04 | 0.74 | 0.04 | 0.70 | 0.04 | 0.70 | 0.05 | 0.64 | 0.04 | 0.05 | 0.02 | 0.79 | 0.02 | 0.54 |
| **891** | 0.95 | 0.61 | 0.95 | 0.61 | 0.96 | 0.73 | 0.96 | 0.73 | 0.96 | 0.69 | 0.85 | 0.50 | 0.92 | 0.15 | 0.90 | 0.60 |
| **892** | 0.51 | 0.91 | 0.51 | 0.91 | 0.45 | 0.91 | 0.45 | 0.91 | 0.59 | 0.94 | 0.51 | 0.95 | 0.11 | 0.85 | 0.09 | 0.89 |
| **893** | 0.08 | 0.73 | 0.08 | 0.73 | 0.05 | 0.73 | 0.05 | 0.73 | 0.04 | 0.73 | 0.07 | 0.62 | 0.64 | 0.97 | 0.93 | 0.97 |
| **894** | 0.07 | 0.91 | 0.07 | 0.91 | 0.09 | 0.91 | 0.09 | 0.91 | 0.07 | 0.91 | 0.09 | 0.91 | 0.06 | 0.87 | 0.12 | 0.90 |
| **895** | 0.15 | 0.71 | 0.15 | 0.71 | 0.27 | 0.71 | 0.27 | 0.71 | 0.36 | 0.71 | 0.11 | 0.51 | 0.57 | 0.92 | 0.69 | 0.92 |
| **896** | 0.33 | 0.96 | 0.33 | 0.96 | 0.50 | 0.96 | 0.50 | 0.96 | 0.73 | 0.96 | 0.92 | 0.97 | 0.33 | 0.97 | 0.28 | 0.97 |
| **897** | 0.03 | 0.58 | 0.03 | 0.58 | 0.03 | 0.58 | 0.03 | 0.58 | 0.03 | 0.58 | 0.03 | 0.60 | 0.02 | 0.58 | 0.02 | 0.58 |
| **898** | 0.68 | 0.96 | 0.68 | 0.96 | 0.68 | 0.96 | 0.68 | 0.96 | 0.68 | 0.96 | 0.77 | 0.96 | 0.31 | 0.96 | 0.47 | 0.96 |
| **899** | 0.55 | 0.79 | 0.55 | 0.79 | 0.55 | 0.79 | 0.55 | 0.79 | 0.55 | 0.79 | 0.84 | 0.79 | 0.61 | 0.79 | 0.43 | 0.79 |
| **900** | 0.06 | 0.30 | 0.06 | 0.30 | 0.06 | 0.30 | 0.06 | 0.30 | 0.06 | 0.30 | 0.05 | 0.30 | 0.12 | 0.33 | 0.13 | 0.33 |
| **901** | 0.42 | 0.58 | 0.42 | 0.58 | 0.42 | 0.58 | 0.42 | 0.58 | 0.42 | 0.58 | 0.44 | 0.58 | 0.66 | 0.81 | 0.75 | 0.81 |
| **902** | 0.29 | 0.20 | 0.29 | 0.20 | 0.29 | 0.20 | 0.29 | 0.20 | 0.29 | 0.20 | 0.19 | 0.20 | 0.51 | 0.28 | 0.56 | 0.28 |
| **903** | 0.29 | 0.29 | 0.29 | 0.29 | 0.29 | 0.29 | 0.29 | 0.29 | 0.24 | 0.29 | 0.22 | 0.29 | 0.19 | 0.62 | 0.39 | 0.62 |
| **904** | 0.10 | 0.70 | 0.10 | 0.70 | 0.10 | 0.70 | 0.10 | 0.70 | 0.10 | 0.70 | 0.10 | 0.70 | 0.19 | 0.76 | 0.17 | 0.87 |
| **905** | 0.82 | 0.62 | 0.82 | 0.62 | 0.82 | 0.62 | 0.82 | 0.62 | 0.74 | 0.62 | 0.74 | 0.62 | 0.94 | 0.90 | 0.96 | 0.93 |
| **906** | 0.03 | 0.38 | 0.03 | 0.38 | 0.03 | 0.38 | 0.03 | 0.38 | 0.03 | 0.38 | 0.03 | 0.38 | 0.02 | 0.05 | 0.03 | 0.06 |
| **907** | 0.42 | 0.31 | 0.42 | 0.31 | 0.42 | 0.31 | 0.42 | 0.31 | 0.43 | 0.31 | 0.43 | 0.31 | 0.23 | 0.10 | 0.37 | 0.18 |
| **908** | 0.04 | 0.19 | 0.04 | 0.19 | 0.04 | 0.19 | 0.04 | 0.19 | 0.03 | 0.15 | 0.03 | 0.15 | 0.04 | 0.18 | 0.04 | 0.12 |
| **909** | 0.07 | 0.47 | 0.07 | 0.47 | 0.07 | 0.47 | 0.07 | 0.47 | 0.04 | 0.16 | 0.04 | 0.16 | 0.03 | 0.33 | 0.03 | 0.31 |
| **910** | 0.78 | 0.91 | 0.78 | 0.91 | 0.78 | 0.91 | 0.78 | 0.91 | 0.55 | 0.86 | 0.55 | 0.86 | 0.19 | 0.90 | 0.23 | 0.74 |
| **911** | 0.83 | 0.40 | 0.83 | 0.40 | 0.83 | 0.40 | 0.83 | 0.40 | 0.93 | 0.20 | 0.93 | 0.20 | 0.44 | 0.37 | 0.34 | 0.55 |
| **912** | 0.03 | 0.92 | 0.03 | 0.92 | 0.03 | 0.92 | 0.03 | 0.92 | 0.04 | 0.87 | 0.04 | 0.87 | 0.15 | 0.76 | 0.09 | 0.67 |
| **913** | 0.98 | 0.79 | 0.98 | 0.79 | 0.98 | 0.79 | 0.98 | 0.79 | 0.98 | 0.94 | 0.98 | 0.94 | 0.98 | 0.82 | 0.98 | 0.92 |
| **914** | 0.21 | 0.09 | 0.21 | 0.09 | 0.21 | 0.09 | 0.21 | 0.09 | 0.96 | 0.21 | 0.96 | 0.21 | 0.38 | 0.19 | 0.77 | 0.19 |
| **915** | 0.05 | 0.51 | 0.05 | 0.51 | 0.05 | 0.51 | 0.05 | 0.51 | 0.04 | 0.76 | 0.04 | 0.76 | 0.04 | 0.74 | 0.04 | 0.79 |
| **916** | 0.03 | 0.27 | 0.03 | 0.27 | 0.03 | 0.27 | 0.03 | 0.27 | 0.04 | 0.37 | 0.04 | 0.37 | 0.04 | 0.41 | 0.03 | 0.41 |
| **917** | 0.04 | 0.03 | 0.04 | 0.03 | 0.04 | 0.03 | 0.04 | 0.03 | 0.04 | 0.04 | 0.04 | 0.04 | 0.02 | 0.05 | 0.02 | 0.05 |
| **918** | 0.02 | 0.14 | 0.02 | 0.14 | 0.02 | 0.14 | 0.02 | 0.14 | 0.02 | 0.14 | 0.02 | 0.14 | 0.02 | 0.10 | 0.02 | 0.10 |
| **919** | 0.03 | 0.04 | 0.03 | 0.04 | 0.03 | 0.04 | 0.03 | 0.04 | 0.03 | 0.04 | 0.03 | 0.04 | 0.04 | 0.03 | 0.05 | 0.03 |
| **920** | **0.11** | **0.04** | **0.11** | **0.04** | **0.11** | **0.04** | **0.11** | **0.04** | **0.07** | **0.04** | **0.07** | **0.04** | **0.10** | **0.04** | **0.09** | **0.04** |
| **921** | **0.04** | **0.23** | **0.04** | **0.23** | **0.04** | **0.23** | **0.04** | **0.23** | **0.03** | **0.17** | **0.03** | **0.17** | **0.34** | **0.23** | **0.34** | **0.23** |
| **922** | **0.94** | **0.21** | **0.94** | **0.21** | **0.94** | **0.21** | **0.94** | **0.21** | **0.92** | **0.20** | **0.92** | **0.20** | **0.90** | **0.94** | **0.91** | **0.94** |
| **923** | **0.11** | **0.07** | **0.11** | **0.07** | **0.11** | **0.07** | **0.11** | **0.07** | **0.04** | **0.04** | **0.04** | **0.04** | **0.02** | **0.14** | **0.02** | **0.14** |
| **924** | **0.97** | **0.76** | **0.97** | **0.76** | **0.97** | **0.76** | **0.97** | **0.76** | **0.88** | **0.18** | **0.88** | **0.18** | **0.25** | **0.19** | **0.32** | **0.19** |
| **925** | **0.08** | **0.97** | **0.08** | **0.97** | **0.08** | **0.97** | **0.08** | **0.97** | **0.04** | **0.96** | **0.03** | **0.96** | **0.03** | **0.80** | **0.03** | **0.80** |
| **926** | **0.97** | **0.92** | **0.97** | **0.92** | **0.97** | **0.92** | **0.97** | **0.92** | **0.98** | **0.45** | **0.98** | **0.45** | **0.97** | **0.10** | **0.96** | **0.10** |
| **927** | **0.94** | **0.26** | **0.94** | **0.26** | **0.94** | **0.26** | **0.94** | **0.26** | **0.96** | **0.73** | **0.95** | **0.73** | **0.44** | **0.09** | **0.67** | **0.08** |
| **928** | 0.04 | 0.05 | 0.04 | 0.05 | 0.04 | 0.05 | 0.04 | 0.05 | 0.05 | 0.05 | 0.03 | 0.08 | 0.04 | 0.18 | 0.04 | 0.37 |
| **929** | 0.02 | 0.27 | 0.02 | 0.27 | 0.02 | 0.27 | 0.02 | 0.27 | 0.02 | 0.23 | 0.02 | 0.65 | 0.39 | 0.25 | 0.37 | 0.09 |
| **930** | 0.06 | 0.22 | 0.06 | 0.22 | 0.06 | 0.22 | 0.06 | 0.22 | 0.08 | 0.39 | 0.05 | 0.24 | 0.03 | 0.91 | 0.03 | 0.95 |
| **931** | 0.12 | 0.04 | 0.12 | 0.04 | 0.12 | 0.04 | 0.12 | 0.04 | 0.67 | 0.07 | 0.05 | 0.06 | 0.03 | 0.43 | 0.03 | 0.27 |
| **932** | 0.05 | 0.07 | 0.05 | 0.07 | 0.05 | 0.07 | 0.05 | 0.07 | 0.04 | 0.07 | 0.03 | 0.15 | 0.42 | 0.58 | 0.66 | 0.57 |
| **933** | 0.97 | 0.85 | 0.97 | 0.85 | 0.97 | 0.85 | 0.97 | 0.85 | 0.95 | 0.89 | 0.85 | 0.87 | 0.54 | 0.19 | 0.63 | 0.28 |
| **934** | 0.04 | 0.04 | 0.04 | 0.04 | 0.04 | 0.04 | 0.04 | 0.04 | 0.03 | 0.05 | 0.03 | 0.06 | 0.03 | 0.08 | 0.04 | 0.13 |
| **935** | 0.16 | 0.49 | 0.16 | 0.49 | 0.16 | 0.49 | 0.16 | 0.49 | 0.20 | 0.49 | 0.28 | 0.48 | 0.23 | 0.74 | 0.25 | 0.74 |
| **936** | 0.06 | 0.23 | 0.06 | 0.23 | 0.06 | 0.23 | 0.06 | 0.23 | 0.06 | 0.23 | 0.84 | 0.58 | 0.06 | 0.23 | 0.07 | 0.23 |
| **937** | 0.37 | 0.95 | 0.37 | 0.95 | 0.37 | 0.95 | 0.37 | 0.95 | 0.20 | 0.95 | 0.24 | 0.92 | 0.06 | 0.95 | 0.06 | 0.95 |
| **938** | 0.25 | 0.33 | 0.25 | 0.33 | 0.25 | 0.33 | 0.25 | 0.33 | 0.30 | 0.33 | 0.17 | 0.39 | 0.40 | 0.33 | 0.61 | 0.33 |
| **939** | 0.23 | 0.73 | 0.23 | 0.73 | 0.23 | 0.73 | 0.23 | 0.73 | 0.37 | 0.73 | 0.29 | 0.73 | 0.39 | 0.73 | 0.41 | 0.73 |
| **940** | 0.96 | 0.88 | 0.96 | 0.88 | 0.96 | 0.88 | 0.96 | 0.88 | 0.97 | 0.88 | 0.96 | 0.88 | 0.97 | 0.88 | 0.97 | 0.88 |
| **941** | 0.10 | 0.54 | 0.10 | 0.54 | 0.10 | 0.54 | 0.10 | 0.54 | 0.17 | 0.54 | 0.11 | 0.54 | 0.16 | 0.54 | 0.16 | 0.54 |
| **942** | 0.29 | 0.94 | 0.29 | 0.94 | 0.29 | 0.94 | 0.29 | 0.94 | 0.51 | 0.94 | 0.32 | 0.94 | 0.41 | 0.94 | 0.41 | 0.94 |
| **943** | 0.94 | 0.71 | 0.94 | 0.71 | 0.94 | 0.71 | 0.94 | 0.71 | 0.97 | 0.71 | 0.97 | 0.71 | 0.95 | 0.71 | 0.95 | 0.71 |
| **944** | 0.02 | 0.43 | 0.02 | 0.43 | 0.02 | 0.43 | 0.02 | 0.43 | 0.03 | 0.31 | 0.03 | 0.31 | 0.03 | 0.31 | 0.03 | 0.31 |
| **945** | 0.21 | 0.93 | 0.21 | 0.93 | 0.21 | 0.93 | 0.21 | 0.93 | 0.22 | 0.80 | 0.22 | 0.80 | 0.21 | 0.80 | 0.21 | 0.80 |
| **946** | 0.02 | 0.08 | 0.02 | 0.08 | 0.02 | 0.08 | 0.02 | 0.08 | 0.02 | 0.06 | 0.02 | 0.06 | 0.02 | 0.04 | 0.02 | 0.04 |
| **947** | 0.40 | 0.04 | 0.40 | 0.04 | 0.40 | 0.04 | 0.40 | 0.04 | 0.03 | 0.03 | 0.03 | 0.03 | 0.02 | 0.03 | 0.02 | 0.03 |
| **948** | 0.82 | 0.74 | 0.82 | 0.74 | 0.82 | 0.74 | 0.82 | 0.74 | 0.81 | 0.06 | 0.74 | 0.06 | 0.69 | 0.06 | 0.69 | 0.06 |
| **949** | 0.05 | 0.98 | 0.05 | 0.98 | 0.05 | 0.98 | 0.05 | 0.98 | 0.04 | 0.92 | 0.05 | 0.92 | 0.94 | 0.52 | 0.94 | 0.52 |
| **950** | 0.38 | 0.42 | 0.38 | 0.42 | 0.38 | 0.42 | 0.38 | 0.42 | 0.30 | 0.58 | 0.39 | 0.53 | 0.50 | 0.39 | 0.50 | 0.39 |
| **951** | 0.88 | 0.94 | 0.88 | 0.94 | 0.88 | 0.94 | 0.88 | 0.94 | 0.97 | 0.86 | 0.97 | 0.91 | 0.97 | 0.96 | 0.97 | 0.96 |
| **952** | 0.11 | 0.33 | 0.11 | 0.33 | 0.11 | 0.33 | 0.11 | 0.33 | 0.71 | 0.23 | 0.73 | 0.46 | 0.11 | 0.33 | 0.11 | 0.33 |
| **953** | 0.06 | 0.78 | 0.06 | 0.78 | 0.06 | 0.78 | 0.06 | 0.78 | 0.07 | 0.26 | 0.45 | 0.64 | 0.35 | 0.14 | 0.35 | 0.14 |
| **954** | 0.97 | 0.41 | 0.97 | 0.41 | 0.97 | 0.41 | 0.97 | 0.41 | 0.98 | 0.93 | 0.97 | 0.95 | 0.97 | 0.69 | 0.97 | 0.69 |
| **955** | 0.12 | 0.32 | 0.12 | 0.32 | 0.12 | 0.32 | 0.12 | 0.32 | 0.34 | 0.08 | 0.15 | 0.21 | 0.66 | 0.04 | 0.66 | 0.04 |
| **956** | 0.03 | 0.83 | 0.03 | 0.83 | 0.03 | 0.76 | 0.03 | 0.76 | 0.03 | 0.93 | 0.03 | 0.94 | 0.02 | 0.91 | 0.02 | 0.91 |
| **957** | 0.65 | 0.17 | 0.65 | 0.17 | 0.57 | 0.12 | 0.57 | 0.12 | 0.21 | 0.57 | 0.48 | 0.07 | 0.79 | 0.44 | 0.79 | 0.44 |
| **958** | 0.83 | 0.48 | 0.83 | 0.48 | 0.88 | 0.49 | 0.88 | 0.49 | 0.57 | 0.08 | 0.47 | 0.13 | 0.87 | 0.77 | 0.87 | 0.77 |
| **959** | 0.48 | 0.47 | 0.48 | 0.47 | 0.66 | 0.56 | 0.66 | 0.56 | 0.25 | 0.38 | 0.19 | 0.49 | 0.45 | 0.59 | 0.45 | 0.59 |
| **960** | 0.03 | 0.89 | 0.03 | 0.89 | 0.03 | 0.90 | 0.03 | 0.90 | 0.47 | 0.64 | 0.33 | 0.47 | 0.46 | 0.95 | 0.46 | 0.95 |
| **961** | 0.76 | 0.63 | 0.76 | 0.63 | 0.79 | 0.68 | 0.79 | 0.68 | 0.85 | 0.34 | 0.77 | 0.34 | 0.24 | 0.37 | 0.24 | 0.37 |
| **962** | 0.93 | 0.61 | 0.93 | 0.61 | 0.91 | 0.48 | 0.91 | 0.48 | 0.65 | 0.81 | 0.94 | 0.81 | 0.57 | 0.32 | 0.57 | 0.32 |
| **963** | 0.05 | 0.10 | 0.05 | 0.10 | 0.06 | 0.10 | 0.06 | 0.10 | 0.03 | 0.12 | 0.03 | 0.10 | 0.05 | 0.69 | 0.05 | 0.69 |
| **964** | 0.06 | 0.23 | 0.06 | 0.23 | 0.06 | 0.23 | 0.06 | 0.23 | 0.36 | 0.52 | 0.04 | 0.28 | 0.03 | 0.80 | 0.03 | 0.80 |
| **965** | 0.05 | 0.13 | 0.05 | 0.13 | 0.06 | 0.13 | 0.06 | 0.13 | 0.04 | 0.14 | 0.04 | 0.40 | 0.53 | 0.46 | 0.53 | 0.46 |
| **966** | 0.03 | 0.72 | 0.03 | 0.72 | 0.03 | 0.72 | 0.03 | 0.72 | 0.05 | 0.13 | 0.03 | 0.20 | 0.16 | 0.68 | 0.16 | 0.68 |
| **967** | 0.03 | 0.27 | 0.03 | 0.27 | 0.03 | 0.27 | 0.03 | 0.27 | 0.03 | 0.29 | 0.04 | 0.13 | 0.06 | 0.13 | 0.06 | 0.13 |
| **968** | 0.92 | 0.76 | 0.92 | 0.76 | 0.92 | 0.76 | 0.92 | 0.76 | 0.97 | 0.55 | 0.89 | 0.92 | 0.44 | 0.54 | 0.44 | 0.54 |
| **969** | 0.07 | 0.05 | 0.07 | 0.05 | 0.07 | 0.05 | 0.07 | 0.05 | 0.15 | 0.11 | 0.14 | 0.08 | 0.13 | 0.04 | 0.13 | 0.04 |
| **970** | 0.06 | 0.22 | 0.06 | 0.22 | 0.06 | 0.22 | 0.06 | 0.22 | 0.15 | 0.22 | 0.05 | 0.20 | 0.17 | 0.59 | 0.17 | 0.59 |
| **971** | 0.74 | 0.22 | 0.74 | 0.22 | 0.74 | 0.22 | 0.74 | 0.22 | 0.58 | 0.20 | 0.95 | 0.39 | 0.27 | 0.48 | 0.27 | 0.48 |
| **972** | 0.12 | 0.43 | 0.12 | 0.43 | 0.12 | 0.43 | 0.12 | 0.43 | 0.10 | 0.43 | 0.18 | 0.38 | 0.00 | 0.00 | 0.00 | 0.00 |

**Supplementary Table S5. Acession numbers for 318 HECT ubiquitin E3 ligases used in Bayesian phylogenetic analysis.**

| **Entry** | **Organism** | | **Common name/Strain** | |
| --- | --- | --- | --- | --- |
| I1FCE7_AMPQE | *Amphimedon queenslandica* | | Sponge | |
| F2U5I7_SALR5 | *Salpingoeca rosetta* | | strain ATCC 50818 / BSB-021 | |
| E4YWL1_OIKDI | *Oikopleura dioica* | | Tunicate | |
| I1F2S5_AMPQE | *Amphimedon queenslandica* | | Sponge | |
| H3EJ01_PRIPA | *Pristionchus pacificus* | | Parasitic nematode | |
| A8WZ81_CAEBR | | *Caenorhabditis briggsae* | |  |
| G0MM60_CAEBE | *Caenorhabditis brenneri* | | Nematode worm | |
| H2VND3_CAEJA | | *Caenorhabditis japonica* | |  |
| E3MFN2_CAERE | *Caenorhabditis remanei* | | Caenorhabditis vulgaris | |
| Q9N2Z7_CAEEL | | *Caenorhabditis elegans* | |  |
| A8QCW2_BRUMA | *Brugia malayi* | | Filarial nematode worm | |
| F1KSN2_ASCSU | *Ascaris suum* | | Pig roundworm | |
| H9K716_APIME | *Apis melifera* | | Honey bee | |
| E0VJS3_PEDHC | *Pediculus humanus subsp. corporis* | | Body louse | |
| D6WLV1_TRICA | *Tribolium castaneum* | | Red flour beetle | |
| G6DEV1_DANPL | *Danaus plexippus* | | Monarch butterfly | |
| B4MVK8_DROWI | *Drosophila willistoni* | | Fruit fly | |
| B4JAH9_DROGR | *Drosophila grimshawi* | | Fruit fly | |
| B4KIY8_DROMO | *Drosophila mojavensis* | | Fruit fly | |
| B4LVA8_DROVI | *Drosophila virilis* | | Fruit fly | |
| Q29LM8_DROPS | *Drosophila pseudoobscura pseudoobscura* | | Fruit fly | |
| B3MJD3_DROAN | *Drosophila ananassae* | | Fruit fly | |
| B4IDF3_DROSE | *Drosophila sechellia* | | Fruit fly | |
| B4NW40_DROYA | *Drosophila yakuba* | | Fruit fly | |
| Q7PW66_ANOGA | *Anopheles gambiae* | | African malaria mosquito | |
| B0WB11_CULQU | *Culex quinquefasciatus* | | Southern house mosquito | |
| A2I859_AEDAE | *Aedes aegypti* | | Yellowfever mosquito | |
| F7BGT4_CIOIN | *Ciona intestinalis* | | Transparent sea squirt | |
| F7AY66_ORNAN | *Ornithorhynchus anatinus* | | Duckbill platypus | |
| E7FEW3_DANRE | *Danio rerio* | | Zebrafish | |
| H2LPB0_ORYLA | *Oryzias latipes* | | Japanese rice fish | |
| H2VDA6_TAKRU | *Takifugu rubripes* | | Japanese pufferfish | |
| Q4RIL1_TETNG | *Tetraodon nigroviridis* | | Spotted green pufferfish | |
| E6ZF41_DICLA | *Dicentrarchus labrax* | | European seabass | |
| G3NR11_GASAC | *Gasterosteus aculeatus* | | Three-spined stickleback | |
| H9GXL4_DANRE | *Danio rerio* | | Zebrafish | |
| H2L639_ORYLA | *Oryzias latipes* | | Japanese rice fish | |
| G3NLP4_GASAC | *Gasterosteus aculeatus* | | Three-spined stickleback | |
| H2TWN5_TAKRU | *Takifugu rubripes* | | Japanese pufferfish | |
| A0JM32_XENTR | *Xenopus tropicalis* | | Western clawed frog | |
| H3BIG6_LATCH | *Latimeria chalumnae* | | West Indian ocean coelacanth | |
| G1KIS2_ANOCA | *Anolis carolinensis* | | Green anole | |
| G3WYP2_SARHA | *Sarcophilus harrisii* | | Tasmanian devil | |
| H0XG55_OTOGA | *Otolemur garnettii* | | Small-eared galago | |
| Q5YB86_RAT | *Rattus norvegicus* | | Rat | |
| G3HAN9_CRIGR | *Cricetulus griseus* | | Chinese hamster | |
| Q571M5_MOUSE | *Mus musculus* | | Mouse | |
| F6QCT8_CALJA | *Callithrix jacchus* | | White-tufted-ear marmoset | |
| A2VE03_BOVIN | *Bos taurus* | | Bovine | |
| G5BPT5_HETGA | *Heterocephalus glaber* | | Naked mole rat | |
| G1P398_MYOLU | *Myotis lucifugus* | | Little brown bat | |
| F6XN76_HORSE | *Equus caballus* | | Horse | |
| E2QXM7_CANFA | *Canis familiaris* | | Dog | |
| D2H434_AILME | *Ailuropoda melanoleuca* | | Giant panda | |
| G1LRF3_AILME | *Ailuropoda melanoleuca* | | Giant panda | |
| G1T9A1_RABIT | *Oryctolagus cuniculus* | | Rabbit | |
| G3T7U0_LOXAF | *Loxodonta africana* | | African elephant | |
| H0VPJ7_CAVPO | *Cavia porcellus* | | Guinea pig | |
| H9ERF6_MACMU | *Macaca mulatta* | | Rhesus macaque | |
| G3S7Y3_GORGO | *Gorilla gorilla gorilla* | | Western lowland gorilla | |
| G1RGD4_NOMLE | *Nomascus leucogenys* | | Northern white-cheeked gibbon | |
| H2P1P9_PONAB | *Pongo abelii* | | Sumatran orangutan | |
| ITCH_HUMAN | *Homo sapiens* | | Human | |
| G3QWC8_GORGO | *Gorilla gorilla gorilla* | | Western lowland gorilla | |
| H0YZC8_TAEGU | *Taeniopygia guttata* | | Zebra finch | |
| E1BVS1_CHICK | *Gallus gallus* | | Chicken | |
| G1MTE4_MELGA | *Meleagris gallopavo* | | Common turkey | |
| G7Q1I7_MACFA | *Macaca fascicularis* | | Crab-eating macaque | |
| Q3UJU3_MOUSE | *Mus musculus* | | Mouse | |
| B4F767_RAT | *Rattus norvegicus* | | Rat | |
| Q4SKN0_TETNG | *Tetraodon nigroviridis* | | Spotted green pufferfish | |
| H2LKD9_ORYLA | *Oryzias latipes* | | Japanese rice fish | |
| H2S001_TAKRU | *Takifugu rubripes* | | Japanese pufferfish | |
| G3NAS4_GASAC | *Gasterosteus aculeatus* | | Three-spined stickleback | |
| E6ZI85_DICLA | *Dicentrarchus labrax* | | European seabass | |
| H3A194_LATCH | *Latimeria chalumnae* | | West Indian ocean coelacanth | |
| Q0P4P5_XENTR | *Xenopus tropicalis* | | Western clawed frog | |
| G1KJA9_ANOCA | *Anolis carolinensis* | | Green anole | |
| H0ZND1_TAEGU | *Taeniopygia guttata* | | Zebra finch | |
| Q5F372_CHICK | *Gallus gallus* | | Chicken | |
| G1NGU6_MELGA | *Meleagris gallopavo* | | Common turkey | |
| G1SEP8_RABIT | *Oryctolagus cuniculus* | | Rabbit | |
| H0VQY5_CAVPO | *Cavia porcellus* | | Guinea pig | |
| Q4V8H7_RAT | *Rattus norvegicus* | | Rat | |
| Q3UV52_MOUSE | *Mus musculus* | | Mouse | |
| F6XJT5_HORSE | *Equus caballus* | | Horse | |
| G3STI3_LOXAF | *Loxodonta africana* | | African elephant | |
| G5BPK1_HETGA | *Heterocephalus glaber* | | Naked mole rat | |
| Q32PG0_BOVIN | *Bos taurus* | | Bovine | |
| E2RSE2_CANFA | *Canis familiaris* | | Dog | |
| G1L7C7_AILME | *Ailuropoda melanoleuca* | | Giant panda | |
| F1RXD3_PIG | *Sus scrofa* | | Pig | |
| H0X671_OTOGA | *Otolemur garnettii* | | Small-eared galago | |
| G7PC59_MACFA | *Macaca fascicularis* | | Crab-eating macaque | |
| G7MZP8_MACMU | *Macaca mulatta* | | Rhesus macaque | |
| F7HIU3_MACMU | *Macaca mulatta* | | Rhesus macaque | |
| F7HIU5_MACMU | *Macaca mulatta* | | Rhesus macaque | |
| F7GXT3_CALJA | *Callithrix jacchus* | | White-tufted-ear marmoset | |
| A8K9T5_HUMAN | *Homo sapiens* | | Human | |
| G1QM19_NOMLE | *Nomascus leucogenys* | | Northern white-cheeked gibbon | |
| H2PQQ8_PONAB | *Pongo abelii* | | Sumatran orangutan | |
| F7BGK2_ORNAN | *Ornithorhynchus anatinus* | | Duckbill platypus | |
| F7ELS3_MONDO | *Monodelphis domestica* | | Gray short-tailed opossum | |
| G3VNS2_SARHA | *Sarcophilus harrisii* | | Tasmanian devil | |
| E9CIS0_CAPO3 | *Capsaspora owczarzaki* | | strain ATCC 30864 | |
| C1K002_BOMMO | *Bombyx mori* | | Silk moth | |
| I1GH83_AMPQE | *Amphimedon queenslandica* | | Sponge | |
| F6W446_CIOIN | *Ciona intestinalis* | | Transparent sea squirt | |
| H2YQB8_CIOSA | *Ciona savignyi* | | Pacific transparent sea squirt | |
| D9I030_CREFO | *Crepidula fornicata* | | Slipper limpet | |
| H2MF89_ORYLA | *Oryzias latipes* | | Japanese rice fish | |
| F1M3F2_RAT | *Rattus norvegicus* | | Rat | |
| H0XD47_OTOGA | *Otolemur garnettii* | | Small-eared galago | |
| SMUF2_XENLA | *Xenopus laevis* | | African clawed frog | |
| H3B3I0_LATCH | *Latimeria chalumnae* | | West Indian ocean coelacanth | |
| G7NJA0_MACMU | *Macaca mulatta* | | Rhesus macaque | |
| F1NJU6_CHICK | *Gallus gallus* | | Chicken | |
| G1T424_RABIT | *Oryctolagus cuniculus* | | Rabbit | |
| F7E372_CALJA | *Callithrix jacchus* | | White-tufted-ear marmoset | |
| H2NUG8_PONAB | *Pongo abelii* | | Sumatran orangutan | |
| G1RY02_NOMLE | *Nomascus leucogenys* | | Northern white-cheeked gibbon | |
| G5BVF0_HETGA | *Heterocephalus glaber* | | Naked mole rat | |
| H0Z2D5_TAEGU | *Taeniopygia guttata* | | Zebra finch | |
| F7EF57_ORNAN | *Ornithorhynchus anatinus* | | Duckbill platypus | |
| H9G425_ANOCA | *Anolis carolinensis* | | Green anole | |
| SMUF2_DANRE | *Danio rerio* | | Zebrafish | |
| H2TDD6_TAKRU | *Takifugu rubripes* | | Japanese pufferfish | |
| G3NVD5_GASAC | *Gasterosteus aculeatus* | | Three-spined stickleback | |
| H2MJY5_ORYLA | *Oryzias latipes* | | Japanese rice fish | |
| Q4RZS8_TETNG | *Tetraodon nigroviridis* | | Spotted green pufferfish | |
| G3Q2K1_GASAC | *Gasterosteus aculeatus* | | Three-spined stickleback | |
| H2T274_TAKRU | *Takifugu rubripes* | | Japanese pufferfish | |
| E9GIQ7_DAPPU | *Daphnia pulex* | | Water flea | |
| H9KKZ0_APIME | *Apis melifera* | | Honey bee | |
| E9IQA5_SOLIN | *Solenopsis invicta* | | Red imported fire ant | |
| F4WXC9_ACREC | *Acromyrmex echinatior* | | Panamanian leafcutter ant | |
| D6WYJ4_TRICA | *Tribolium castaneum* | | Red flour beetle | |
| B4J793_DROGR | *Drosophila grimshawi* | | Fruit fly | |
| B4KN05_DROMO | *Drosophila mojavensis* | | Fruit fly | |
| B4LMV7_DROVI | *Drosophila virilis* | | Fruit fly | |
| B4MIS4_DROWI | *Drosophila willistoni* | | Fruit fly | |
| Q28Z37_DROPS | *Drosophila pseudoobscura pseudoobscura* | | Fruit fly | |
| B3MIF7_DROAN | *Drosophila ananassae* | | Fruit fly | |
| SMUF1_DROME | *Drosophila melanogaster* | | Fruit fly | |
| B4HMY0_DROSE | *Drosophila sechellia* | | Fruit fly | |
| B3NMK5_DROER | *Drosophila erecta* | | Fruit fly | |
| B4P5D7_DROYA | *Drosophila yakuba* | | Fruit fly | |
| G4TGW0_PIRID | *Piriformospora indica* | | strain DSM 11827 | |
| F4PCI9_BATDJ | *Batrachochytrium dendrobatidis* | | strain JAM81 / FGSC 10211 | |
| PUB3_SCHPO | *Schizosaccharomyces pombe* | | strain 972 / ATCC 24843 | |
| B6K793_SCHJY | *Schizosaccharomyces japonicus* | | strain yFS275 / FY16936 | |
| F4RCD2_MELLP | *Melampsora larici-populina* | | strain 98AG31 / pathotype 3-4-7 | |
| G7E4V7_MIXOS | *Mixia osmundae* | | strain CBS 9802 / IAM 14324 / JCM 22182 / KY 12970 | |
| Q6CNC7_KLULA | *Kluyveromyces lactis* | | strain ATCC 8585 / CBS 2359 / DSM 70799 / NBRC 1267 / NRRL Y-1140 / WM37 (*Candida sphaerica*) | |
| G8JXU0_ERECY | *Eremothecium cymbalariae* | | strain CBS 270.75 / DBVPG 7215 / KCTC 17166 / NRRL Y-17582 | |
| G8BSC3_TETPH | *Tetrapisispora phaffii* | | strain ATCC 24235 / CBS 4417 / NBRC 1672 / NRRL Y-8282 / UCD 70-5 (*Fabospora phaffii*) | |
| A7THX3_VANPO | *Vanderwaltozyma polyspora* | | strain ATCC 22028 / DSM 70294 | |
| Q75AI2_ASHGO | *Ashbya gossypii* | | strain ATCC 10895 / CBS 109.51 / FGSC 9923 / NRRL Y-1056 (*Eremothecium gossypii*) | |
| C5DHT2_LACTC | *Lachancea thermotolerans* | | strain ATCC 56472 / CBS 6340 / NRRL Y-8284 (*Kluyveromyces thermotolerans*) | |
| Q6FN71_CANGA | *Candida glabrata* | | strain ATCC 2001 / CBS 138 / JCM 3761 / NBRC 0622 / NRRL Y-65 (*Torulopsis glabrata*) | |
| H2AQ58_KAZAF | *Kazachstania africana* | | strain ATCC 22294 / BCRC 22015 / CBS 2517 / CECT 1963 / NBRC 1671 / NRRL Y-8276 (*Kluyveromyces africanus*) | |
| C5E186_ZYGRC | *Zygosaccharomyces rouxii* | | strain ATCC 2623 / CBS 732 / NBRC 1130 / NCYC 568 / NRRL Y-229 | |
| G8ZWB6_TORDC | *Torulaspora delbrueckii* | | strain ATCC 10662 / CBS 1146 / NBRC 0425 / NCYC 2629 / NRRL Y-866 (*Candida colliculosa*) | |
| G0W670_NAUDC | *Naumovozyma dairenensis* | | strain ATCC 10597 / BCRC 20456 / CBS 421 / NBRC 0211 / NRRL Y-12639 | |
| G0VAL2_NAUCC | *Naumovozyma castellii* | | strain ATCC 76901 / CBS 4309 / NBRC 1992 / NRRL Y-12630 (*Saccharomyces castellii*) | |
| H0GU13_9SACH | *Saccharomyces cerevisiae x Saccharomyces kudriavzevii* | | strain VIN7 | |
| RSP5_YEAST | *Saccharomyces cerevisiae* | | strain ATCC 204508 / S288c | |
| C4R0F0_PICPG | *Komagataella pastoris* | | strain GS115 / ATCC 20864 (*Pichia pastoris*) | |
| G8YBU3_PICSO | *Pichia sorbitophila* | | strain ATCC MYA-4447 / BCRC 22081 / CBS 7064 / NBRC 10061 / NRRL Y-12695 | |
| A5DYH3_LODEL | *Lodderomyces elongisporus* | | strain ATCC 11503 / CBS 2605 / JCM 1781 / NBRC 1676 / NRRL YB-4239 (*Saccharomyces elongisporus*) | |
| G8BJS4_CANPC | *Candida parapsilosis* | | strain CDC 317 / ATCC MYA-4646 (*Monilia parapsilosis*) | |
| H8X450_CANO9 | *Candida orthopsilosis* | | strain 90-125 | |
| C4Y446_CLAL4 | *Clavispora lusitaniae* | | strain ATCC 42720 (*Candida lusitaniae*) | |
| B9WCC0_CANDC | *Candida dubliniensis* | | strain CD36 / ATCC MYA-646 / CBS 7987 / NCPF 3949 / NRRL Y-17841 | |
| Q59Y21_CANAL | *Candida albicans* | | strain SC5314 / ATCC MYA-2876 | |
| A5DE55_PICGU | *Meyerozyma guilliermondii* | | strain ATCC 6260 / CBS 566 / DSM 6381 / JCM 1539 / NBRC 10279 / NRRL Y-324 (*Candida guilliermondii*) | |
| G3B1C5_CANTC | *Candida tenuis* | | strain ATCC 10573 / BCRC 21748 / CBS 615 / JCM 9827 / NBRC 10315 / NRRL Y-1498 / VKM Y-70 | |
| Q6BT41_DEBHA | *Debaryomyces hansenii* | | strain ATCC 36239 / CBS 767 / JCM 1990 / NBRC 0083 / IGC 2968 (*Torulaspora hansenii*) | |
| A3LTS6_PICST | *Scheffersomyces stipitis* | | strain ATCC 58785 / CBS 6054 / NBRC 10063 / NRRL Y-11545 (*Pichia stipitis*) | |
| G3AKG2_SPAPN | *Spathaspora passalidarum* | | strain NRRL Y-27907 / 11-Y1 | |
| Q6C5H0_YARLI | *Yarrowia lipolytica* | | strain CLIB 122 / E 150 (*Candida lipolytica*) | |
| PUB1_SCHPO | *Schizosaccharomyces pombe* | | strain 972 / ATCC 24843 | |
| B6K5J7_SCHJY | *Schizosaccharomyces japonicus* | | strain yFS275 / FY16936 | |
| I1C582_RHIO9 | *Rhizopus delemar* | | strain RA 99-880 / ATCC MYA-4621 / FGSC 9543 / NRRL 43880 (*Rhizopus arrhizus var. delemar*) | |
| A7EEG8_SCLS1 | *Sclerotinia sclerotiorum* | | strain ATCC 18683 / 1980 / Ss-1 (*Whetzelinia sclerotiorum*) | |
| G2YHG2_BOTF4 | *Botryotinia fuckeliana* | | strain T4 (*Botrytis cinerea*) | |
| F0XSL2_GROCL | *Grosmannia clavigera* | | strain kw1407 / UAMH 11150 (*Graphiocladiella clavigera*) | |
| G0S9J5_CHATD | *Chaetomium thermophilum* | | strain DSM 1495 / CBS 144.50 / IMI 039719 | |
| B2AE23_PODAN | *Podospora anserina* | | strain S / ATCC MYA-4624 / DSM 980 / FGSC 10383 | |
| G3J355_CORMM | *Cordyceps militaris* | | strain CM01 | |
| F7W637_SORMK | *Sordaria macrospora* | | strain ATCC MYA-333 / DSM 997 / K | |
| F8MXS6_NEUT8 | *Neurospora tetrasperma* | | strain FGSC 2508 / ATCC MYA-4615 / P0657 | |
| Q6M906_NEUCS | | *Neurospora crassa* | |  |
| G2QFP4_THIHA | *Thielavia heterothallica* | | strain ATCC 42464 / BCRC 31852 / DSM 1799 | |
| G2QUJ5_THITE | *Thielavia terrestris* | | strain ATCC 38088 / NRRL 8126 | |
| G0RCU2_HYPJQ | *Hypocrea jecorina* | | strain QM6a | |
| G9P537_HYPAI | *Hypocrea atroviridis* | | strain ATCC 20476 / IMI 206040 | |
| G9N9I7_HYPVG | *Hypocrea virens* | | strain Gv29-8 / FGSC 10586 (Trichoderma virens) | |
| C7YR46_NECH7 | *Nectria haematococca* | | strain 77-13-4 / ATCC MYA-4622 / FGSC 9596 / MPVI | |
| I1S0N8_GIBZE | *Gibberella zeae* | | strain PH-1 / ATCC MYA-4620 / FGSC 9075 / NRRL 31084 (*Fusarium graminearum*) | |
| G4MUH5_MAGO7 | *Magnaporthe oryzae* | | strain 70-15 / ATCC MYA-4617 / FGSC 8958 (*Pyricularia oryzae*) | |
| G2WR90_VERDV | *Verticillium dahliae* | | strain VdLs.17 / ATCC MYA-4575 / FGSC 10137 | |
| E3QVY1_COLGM | *Colletotrichum graminicola* | | strain M1.001 / M2 / FGSC 10212 (*Glomerella graminicola*) | |
| H1VJJ9_COLHI | *Colletotrichum higginsianum* | | strain IMI 349063 | |
| H6BUF2_EXODN | *Exophiala dermatitidis* | | strain ATCC 34100 / CBS 525.76 / NIH/UT8656 (*Wangiella dermatitidis*) | |
| B6Q6Z7_PENMQ | *Penicillium marneffei* | | strain ATCC 18224 / CBS 334.59 / QM 7333 | |
| B8LVE5_TALSN | *Talaromyces stipitatus* | | strain ATCC 10500 / CBS 375.48 / QM 6759 / NRRL 1006 | |
| B6H7J0_PENCW | *Penicillium chrysogenum* | | strain ATCC 28089 / DSM 1075 / Wisconsin 54-1255 | |
| RSP5_EMENI | *Emericella nidulans* | | strain FGSC A4 / ATCC 38163 / CBS 112.46 / NRRL 194 / M139 | |
| RSP5_ASPCL | *Aspergillus clavatus* | | strain ATCC 1007 / CBS 513.65 / DSM 816 / NCTC 3887 / NRRL 1 | |
| RSP5_NEOFI | *Neosartorya fischeri* | | strain ATCC 1020 / DSM 3700 / FGSC A1164 / NRRL 181 | |
| RSP5_ASPNC | *Aspergillus niger* | | strain CBS 513.88 / FGSC A1513 | |
| RSP5_ASPOR | *Aspergillus oryzae* | | strain ATCC 42149 / RIB 40 | |
| RSP5_ASPTN | *Aspergillus terreus* | | strain NIH 2624 / FGSC A1156 | |
| C5G0M2_ARTOC | *Arthroderma otae* | | strain ATCC MYA-4605 / CBS 113480 | |
| E4V6K4_ARTGP | *Arthroderma gypseum* | | strain ATCC MYA-4604 / CBS 118893 | |
| F2SLC2_TRIRC | *Trichophyton rubrum* | | strain ATCC MYA-4607 / CBS 118892 | |
| C1GYI1_PARBA | *Paracoccidioides lutzii* | | strain ATCC MYA-826 / Pb01 | |
| C5GFJ5_AJEDR | *Ajellomyces dermatitidis* | | strain ER-3 / ATCC MYA-2586 | |
| C6H2E0_AJECH | *Ajellomyces capsulatus* | | strain H143 (*Histoplasma capsulatum*) | |
| C4JTJ1_UNCRE | *Uncinocarpus reesii* | | strain UAMH 1704 | |
| C5P5X9_COCP7 | *Coccidioides posadasii* | | strain C735 | |
| E9CRR5_COCPS | *Coccidioides posadasii* | | strain RMSCC 757 / Silveira | |
| D5GP79_TUBMM | *Tuber melanosporum* | | strain Mel28 | |
| G1XQ47_ARTOA | *Arthrobotrys oligospora* | | strain ATCC 24927 / CBS 115.81 / DSM 1491 (*Didymozoophaga oligospora*) | |
| F9XPD8_MYCGM | *Zymoseptoria tritici* | | strain CBS 115943 / IPO323 *(Septoria tritici*) | |
| B2W179_PYRTR | *Pyrenophora tritici-repentis* | | strain Pt-1C-BFP (*Drechslera tritici-repentis*) | |
| Q0UT17_PHANO | *Phaeosphaeria nodorum* | | strain SN15 / ATCC MYA-4574 / FGSC 10173 (*Septoria nodorum*) | |
| Q5KC94_CRYNJ | *Cryptococcus neoformans var. neoformans serotype D* | | strain JEC21 / ATCC MYA-565 | |
| E6RA83_CRYGW | *Cryptococcus gattii serotype B* | | strain WM276 / ATCC MYA-4071 (*Cryptococcus bacillisporus*) | |
| G4TEW3_PIRID | *Piriformospora indica* | | strain DSM 11827 | |
| F8Q5V0_SERL3 | *Serpula lacrymans var. lacrymans* | | strain S7.3 | |
| D8PWI5_SCHCM | *Schizophyllum commune* | | strain H4-8 / FGSC 9210 | |
| A8NGX0_COPC7 | *Coprinopsis cinerea* | | strain Okayama-7 / 130 / ATCC MYA-4618 / FGSC 9003 | |
| B0D3E8_LACBS | *Laccaria bicolor* | | strain S238N-H82 / ATCC MYA-4686 | |
| A8PR54_MALGO | *Malassezia globosa* | | strain ATCC MYA-4612 / CBS 7966 | |
| Q4PGV0_USTMA | *Ustilago maydis* | | strain 521 / FGSC 9021 | |
| E6ZL78_SPORE | *Sporisorium reilianum* | | strain SRZ2 | |
| F8NYY5_SERL9 | *Serpula lacrymans var. lacrymans* | | strain S7.9 | |
| D8PXH3_SCHCM | *Schizophyllum commune* | | strain H4-8 / FGSC 9210 | |
| A8NRA0_COPC7 | *Coprinopsis cinerea* | | strain Okayama-7 / 130 / ATCC MYA-4618 / FGSC 9003 (*Hormographiella aspergillata*) | |
| B0CVR2_LACBS | *Laccaria bicolor* | | strain S238N-H82 / ATCC MYA-4686 (*Laccaria laccata var. bicolor*) | |
| E9BYM4_CAPO3 | *Capsaspora owczarzaki* | | strain ATCC 30864 | |
| E4XK29_OIKDI | *Oikopleura dioica* | | Tunicate | |
| E1G1V3_LOALO | *Loa loa* | | Eye worm | |
| A8Y1X0_CAEBR | | *Caenorhabditis briggsae* | |  |
| E3LXJ9_CAERE | *Caenorhabditis remanei* | | Caenorhabditis vulgaris | |
| A7RR93_NEMVE | *Nematostella vectensis* | | Starlet sea anemone | |
| T1K985_TETUR | *Tetranychus urticae* | | Two-spotted spider mite | |
| Q16Z11_AEDAE | *Aedes aegypti* | | Yellowfever mosquito | |
| Q7PQR5_ANOGA | *Anopheles gambiae* | | African malaria mosquito | |
| L7ML19_9ACAR | | *Rhipicephalus pulchellus* | |  |
| V5IJJ8_IXORI | *Ixodes ricinus* | | Common tick | |
| B7Q8Q0_IXOSC | *Ixodes scapularis* | | Black-legged tick | |
| B4J3I2_DROGR | *Drosophila grimshawi* | | Fruit fly | |
| B4KZK1_DROMO | *Drosophila mojavensis* | | Fruit fly | |
| B4LF02_DROVI | *Drosophila virilis* | | Fruit fly | |
| B4N6E6_DROWI | *Drosophila willistoni* | | Fruit fly | |
| B4HBV9_DROPE | *Drosophila persimilis* | | Fruit fly | |
| Q8IQR5_DROME | *Drosophila melanogaster* | | Fruit fly | |
| B3M4J8_DROAN | *Drosophila ananassae* | | Fruit fly | |
| B3NHT9_DROER | *Drosophila erecta* | | Fruit fly | |
| B4PJM0_DROYA | *Drosophila yakuba* | | Fruit fly | |
| B4HKU0_DROSE | *Drosophila sechellia* | | Fruit fly | |
| NEDD4_DROME | *Drosophila melanogaster* | | Fruit fly | |
| Q7QE76_ANOGA | *Anopheles gambiae* | | African malaria mosquito | |
| Q17HX3_AEDAE | *Aedes aegypti* | | Yellowfever mosquito | |
| U5EU13_9DIPT | | *Corethrella appendiculata* | |  |
| D6WQL3_TRICA | *Tribolium castaneum* | | Red flour beetle | |
| E0VJ10_PEDHC | *Pediculus humanus subsp. corporis* | | Body louse | |
| E9GKW9_DAPPU | *Daphnia pulex* | | Water flea | |
| H9KIH6_APIME | *Apis melifera* | | Honey bee | |
| E2BXY9_HARSA | *Harpegnathos saltator* | | Jerdon's jumping ant | |
| E2AAW9_CAMFO | *Camponotus floridanus* | | Florida carpenter ant | |
| F4W547_ACREC | *Acromyrmex echinatior* | | Panamanian leafcutter ant | |
| D2XNH4_SACKO | *Saccoglossus kowalevskii* | | Acorn worm | |
| F6PPP8_CIOIN | *Ciona intestinalis* | | Transparent sea squirt | |
| O42573_XENLA | *Xenopus laevis* | | African clawed frog | |
| Q6DIR6_XENTR | *Xenopus tropicalis* | | Western clawed frog | |
| H1A4F6_TAEGU | *Taeniopygia guttata* | | Zebra finch | |
| F1NBQ7_CHICK | *Gallus gallus* | | Chicken | |
| G1RA08_NOMLE | *Nomascus leucogenys* | | Northern white-cheeked gibbon | |
| F7H450_MACMU | *Macaca mulatta* | | Rhesus macaque | |
| G5AYX9_HETGA | *Heterocephalus glaber* | | Naked mole rat | |
| G3RD09_GORGO | *Gorilla gorilla gorilla* | | Western lowland gorilla | |
| G7NKS3_MACMU | *Macaca mulatta* | | Rhesus macaque | |
| G1M9J3_AILME | *Ailuropoda melanoleuca* | | Giant panda | |
| G1T7I0_RABIT | *Oryctolagus cuniculus* | | Rabbit | |
| NED4L_MOUSE | *Mus musculus* | | Mouse | |
| F7HG24_CALJA | *Callithrix jacchus* | | White-tufted-ear marmoset | |
| NED4L_PONAB | *Pongo abelii* | | Sumatran orangutan | |
| H0VCR8_CAVPO | *Cavia porcellus* | | Guinea pig | |
| F1QP93_DANRE | *Danio rerio* | | Zebrafish | |
| H2LN92_ORYLA | *Oryzias latipes* | | Japanese rice fish | |
| G3PVV5_GASAC | *Gasterosteus aculeatus* | | Three-spined stickleback | |
| H2TZN1_TAKRU | *Takifugu rubripes* | | Japanese pufferfish | |
| H2TZN7_TAKRU | *Takifugu rubripes* | | Japanese pufferfish | |
| Q4SQD5_TETNG | *Tetraodon nigroviridis* | | Spotted green pufferfish | |
| Q3KRP0_DANRE | *Danio rerio* | | Zebrafish | |
| G3PTH6_GASAC | *Gasterosteus aculeatus* | | Three-spined stickleback | |
| H3C5U3_TETNG | *Tetraodon nigroviridis* | | Spotted green pufferfish | |
| H2V9K3_TAKRU | *Takifugu rubripes* | | Japanese pufferfish | |
| H3B711_LATCH | *Latimeria chalumnae* | | West Indian ocean coelacanth | |
| F6ST62_XENTR | *Xenopus tropicalis* | | Western clawed frog | |
| G3VRS8_SARHA | *Sarcophilus harrisii* | | Tasmanian devil | |
| H0Z7N9_TAEGU | *Taeniopygia guttata* | | Zebra finch | |
| E1BWY8_CHICK | *Gallus gallus* | | Chicken | |
| G1N2I2_MELGA | *Meleagris gallopavo* | | Common turkey | |
| NEDD4_MOUSE | *Mus musculus* | | Mouse | |
| NEDD4_RAT | *Rattus norvegicus* | | Rat | |
| H0UUG7_CAVPO | *Cavia porcellus* | | Guinea pig | |
| F6RN24_HORSE | *Equus caballus* | | Horse | |
| G1Q969_MYOLU | *Myotis lucifugus* | | Little brown bat | |
| H2NNB6_PONAB | *Pongo abelii* | | Sumatran orangutan | |
| F7HIT1_MACMU | *Macaca mulatta* | | Rhesus macaque | |
| F7DB99_CALJA | *Callithrix jacchus* | | White-tufted-ear marmoset | |
